# Comparative Physiological and Metabolomic Responses of *Procambarus clarkii* and *Macrobrachium nipponense* to Combined Salinity–pH and NaHCO_3_ Alkalinity Stress

**DOI:** 10.3390/ani16172778

**Published:** 2026-09-03

**Authors:** Meili Chi, Shun Cheng, Wenping Jiang, Junzhi Luo, Shili Liu, Jianbo Zheng, Chao Zhu, Miao Peng, Fei Li

**Affiliations:** Key Laboratory of Healthy Freshwater Aquaculture, Zhejiang Institute of Freshwater Fisheries (Zhejiang Freshwater Fishery Environmental Monitoring Station), Ministry of Agriculture and Rural Affairs, Huzhou 313001, China; chimeili83404109@126.com (M.C.);

**Keywords:** *Procambarus clarkii*, *Macrobrachium nipponense*, juveniles, combined saline–pH stress, NaHCO_3_ alkalinity, oxidative stress, metabolomics

## Abstract

*Procambarus clarkii* and *Macrobrachium nipponense* are two freshwater shrimp species used in rice-farming systems. To guide their cultivation in saline–alkaline waters, two 96 h experiments were conducted on juvenilels of each species using 540 juveniles per species (initial body length: 1.51 ± 0.16 cm for *P. clarkii* and 0.88 ± 0.03 cm for *M. nipponense*): combined salinity–pH stress (0/15.0/25.0‰ at pH 7.4/9.0/10.0) and NaHCO_3_ alkalinity stress (0/10/20 mmol/L). Under salinity–pH stress, survival of *P. clarkii* was 100%, 72.23%, and 53.33%, respectively; for *M. nipponense*, it was 93.33%, 61.13%, and 23.33%. Under alkalinity stress, survival of *P. clarkii* was 92.22%, 82.22%, and 61.11%; for *M. nipponense,* it was 91.11%, 65.55%, and 46.67%. In both species, superoxide dismutase, acid phosphatase, and alkaline phosphatase decreased while malondialdehyde increased with stress intensity. Metabolomics revealed species-specific reprogramming: *P. clarkii* upregulated protective lipids (e.g., glycerophosphoethanolamines, fatty acid esters), whereas *M. nipponense* showed depletion of critical osmolytes (glycerophosphocholines) and accumulation of apoptosis-related ceramides. KEGG analysis confirmed that energy metabolism, amino acid homeostasis, and ABC transporters were primary pathways affected. In conclusion, extreme stress significantly reduced survival, suppressed immunity, and triggered metabolic reprogramming in both species, with *P. clarkii* showing substantially higher tolerance. For cultivation, *P. clarkii* is preferred in moderately high saline–alkaline waters, while *M. nipponense* requires low-alkalinity freshwater with strict monitoring. These findings support species-specific management of saline–alkaline rice–shrimp systems.

## Simple Summary

Saline–alkaline water is widely distributed but remains underutilized for aquaculture due to high salinity, pH, and alkalinity stress. This study compared the tolerance of juvenile individuals of two rice-field shrimp species—the red swamp crayfish (*Procambarus clarkii*) and the oriental river prawn (*Macrobrachium nipponense*)—to combined salinity–pH and NaHCO_3_ alkalinity stress. We measured survival rates, antioxidant/immunity enzyme activities (SOD, MDA, ACP, AKP), and hepatopancreas metabolic profiles. *P. clarkii* tolerated both stress types much better than *M. nipponense*. Under severe conditions (25.0‰/pH 10.0 or 20 mmol/L NaHCO_3_), *P. clarkii* survival (53.33% and 61.11%) significantly exceeded that of *M. nipponense* (23.33% and 46.67%). Both species exhibited oxidative damage and immune suppression, but *M. nipponense* was more severely affected. Metabolomics showed that *P. clarkii* activated protective lipid pathways, while *M. nipponense* displayed osmolyte depletion and cell death signals. For farmers, *P. clarkii* is preferable in moderately saline–alkaline waters, whereas *M. nipponense* requires low-alkalinity freshwater with careful management. This study offers practical guidance for sustainable use of saline–alkaline water resources in shrimp and crayfish farming.

## 1. Introduction

*Procambarus clarkii* (red swamp crayfish) and *Macrobrachium nipponense* (oriental river prawn) are two economically important freshwater crustacean species that are particularly well-suited for integrated rice-farming systems [1,2,3]. *P. clarkii* accounts for over 70% of global crayfish production [1], while *M. nipponense* ranks third among freshwater shrimps in China with annual production exceeding 200,000 tonnes [2,3]. Both species possess advantageous biological traits, including rapid growth rates, strong environmental adaptability, and well-established hatchery technologies, making them ideal candidates for expanding aquaculture into non-traditional areas [3,4,5]. However, commercial cultivation remains concentrated in the Yangtze River Basin, whereas vast central and western regions of China, particularly the northwestern territories, possess extensive saline–alkaline water resources that remain largely untapped [6].

Globally, saline–alkaline water bodies are characterized by high salinity, high alkalinity, elevated pH, imbalanced ion ratios, and poor buffering capacity, posing significant challenges for most freshwater species [7]. In China alone, approximately 99.13 million hectares of saline–alkaline land and 46 million hectares of saline–alkaline water are distributed across 19 provinces [8]. Among the physicochemical factors, salinity, alkalinity, and pH are the most critical parameters affecting aquatic animal health and production [9]. Salinity influences growth, osmoregulation, and ion balance [8]. Alkalinity is a measure of the water’s buffering capacity, primarily determined by bicarbonate (HCO_3_^−^) and carbonate (CO_3_^2−^) ions, and impacts tissue function and immune responses [10]. pH is a measure of hydrogen ion activity, indicating the acidity or basicity of the water, and high pH disrupts ammonia excretion and acid–base balance [11]. Although alkalinity and pH are often correlated in natural waters, they represent distinct physicochemical properties and can exert different physiological effects on aquatic organisms.

Previous studies have established that *P. clarkii* exhibits moderate tolerance to saline conditions, with strong adaptability below 6.0‰ salinity but significant declines above this threshold [11]. Li [11] reported that juveniles survived at 0–14.0‰, with optimal growth at 0–6.0‰. Tao et al. [8] reported a 96 h median lethal concentration (LC_50_) of pH 10.194 for *P. clarkii*, with high pH inducing oxidative stress and tissue damage. Metabolomic studies further revealed that carbonate alkalinity stress triggers metabolic reprogramming in crayfish larvae, with a 96 h LC_50_ of 16.03 mmol/L [7]. For *M. nipponense*, Jin et al. [12] demonstrated that alkalinity stress alters biochemical parameters and induces oxidative stress. Fan et al. [13] identified key osmoregulatory and energy metabolism pathways under salinity stress using RNA-seq. The selectively bred “Taihu No. 3” strain showed a 96 h LC_50_ salinity of 11.841‰ [3]. Bao [14] reported only 30% survival at pH 9.8. Regarding alkalinity, Jin et al. [15] reported a 96 h LC_50_ of 14.42 mmol/L and a safe concentration of 4.71 mmol/L, indicating unsuitability for high-alkalinity aquaculture [15,16].

Despite growing research on individual stressors, two critical gaps remain. First, most previous investigations have examined salinity and pH as isolated factors, leaving their combined effects—and their comparison with alkalinity alone—largely unexplored. Second, no study has directly compared the physiological and metabolic responses of *P. clarkii* and *M. nipponense* under identical experimental conditions. This direct comparative approach under standardized conditions represents the key novelty of our study. We tested the null hypothesis that there is no significant difference in tolerance to combined salinity–pH stress and NaHCO_3_ alkalinity stress between *P. clarkii* and *M. nipponense* juveniles. To address this, the present study integrated survival, enzyme activities (SOD, MDA, ACP, AKP), and metabolomic profiles to (1) compare species-specific tolerance thresholds; (2) elucidate common and distinct physiological and metabolic mechanisms; and (3) provide a scientific basis for sustainable saline–alkaline aquaculture. The findings will support species and water quality management, broadening the geographical scope of crustacean farming.

## 2. Materials and Methods

### 2.1. Experimental Animals and Acclimation

The experiments were conducted at the Zhejiang Institute of Freshwater Fisheries in Huzhou, China. Healthy juvenile *P. clarkii* (average body length: 1.51 ± 0.16 cm) and *M. nipponense* (average body length: 0.88 ± 0.03 cm) were obtained from a commercial hatchery and transported to the laboratory. Prior to the experiments, all animals were acclimated for 7 days in holding tanks under conditions identical to those of the control group to minimize handling stress and ensure stable physiological baselines. During acclimation, the animals were fed a commercial pelleted diet once daily, and feeding was stopped 24 h before the start of the experiments to avoid residual feed affecting water quality. Although the two species differed in absolute body size, all individuals were at the same developmental stage (post-larval juveniles, approximately 7 days after metamorphosis), which ensured comparability of stress responses between species. The red swamp crayfish is inherently a larger species than the oriental river prawn at all life stages, and our experiments were designed to compare responses at the same ontogenetic stage rather than at identical physical dimensions. Furthermore, our experimental design evaluated relative responses (percentage changes in enzyme activities and survival rates) rather than absolute physiological values, minimizing the confounding effects of absolute body size. After acclimation, 540 individuals of each species were randomly selected and distributed into the experimental system (3 treatments × 3 replicates × 30 individuals = 270 individuals per species per experiment, with two experiments conducted separately).

### 2.2. Experimental Design

All experiments were conducted in 100 L polypropylene tanks at a stocking density of 60 individuals/m^2^ (30 individuals per tank), with three replicate tanks per treatment. This density was chosen based on preliminary trials to avoid overcrowding stress while maintaining statistical power. No feed was provided during the exposure periods to eliminate dietary effects on the measured physiological and metabolic parameters. A 50% daily water exchange was performed using pre-adjusted water matching the respective treatment conditions to maintain stable water chemistry throughout the experiments. Water quality during the experiments was monitored daily and maintained as follows: temperature 21.0 ± 1.0 °C, ammonia-N 0.05–0.20 mg/L, nitrite 0.05–0.10 mg/L, and dissolved oxygen 6.0–7.0 mg/L (achieved by continuous aeration). All physicochemical parameters were within acceptable ranges for both species.

#### 2.2.1. Experiment 1: Combined Salinity and pH Stress

Juveniles of each species were exposed for 96 h to three treatments (0 mmol/L NaHCO_3_):

Group 1 (Control): Salinity 0‰, pH 7.4.

Group 2: Salinity 15.0‰, pH 9.0.

Group 3: Salinity 25.0‰, pH 10.0.

The selected salinity and pH levels were based on preliminary range-finding experiments and published LC_50_ data [8,9,17,18,19,20]. This combined salinity–pH design was chosen to simulate the concurrent occurrence of these stressors in natural saline–alkaline water bodies. However, it should be noted that this approach does not allow separation of the individual effects of salinity and pH.

Salinity was adjusted using commercial sea salt (Instant Ocean^®^) dissolved in dechlorinated tap water and monitored daily with a digital salinity meter (precision: ± 0.1‰), maintaining fluctuations within ±0.5‰ of the target values. The choice of 15.0‰ and 25.0‰ salinity levels was based on preliminary range-finding experiments and previous studies indicating that these concentrations represent moderate and high-stress levels for both species, respectively. The pH was adjusted using 0.01 mol/L HCl or NaOH solutions and measured with a calibrated digital pH meter, with variations kept within ± 0.1 of the target value. At the end of the exposure, survival was recorded, and hepatopancreas samples were collected for enzyme activity assays and metabolomic analysis.

#### 2.2.2. Experiment 2: NaHCO_3_ Alkalinity Stress

Juveniles of each species were exposed for 96 h to three NaHCO_3_ concentrations (salinity 0‰, pH 7.4):

Group 1 (Control): 0 mmol/L NaHCO_3_.

Group 4 (Low alkalinity): 10 mmol/L NaHCO_3_.

Group 5 (High alkalinity): 20 mmol/L NaHCO_3_.

The selection of 10 and 20 mmol/L NaHCO_3_ was based on previous studies on alkalinity tolerance in related crustacean species and preliminary tests, which indicated that these concentrations represent sub-lethal and near-lethal levels for *M. nipponense*, respectively, while remaining within the tolerable range for *P. clarkii*.

Test water was prepared using dechlorinated tap water that had been treated with quicklime (CaO) for disinfection, followed by activated carbon filtration, ultraviolet sterilization, and vigorous aeration for 48 h to remove residual chlorine and stabilize dissolved oxygen levels. This treatment resulted in negligible background alkalinity (<0.5 mmol/L). Alkaline water of the desired concentrations was prepared by dissolving analytical-grade NaHCO_3_ (Tianjin Beichen Fangzheng Reagent Factory, purity ≥ 99.5%) in the treated water. The alkalinity of each treatment was verified by acid–base titration and stabilized for two consecutive days before introducing the animals to ensure consistent exposure conditions. At the end of the exposure, survival was recorded, and hepatopancreas samples were collected for subsequent analyses.

### 2.3. Sample Collection and Ethical Statement

At the end of each experiment, all individuals were euthanized by rapid cooling on ice followed by severance of the rostral region, in accordance with approved animal ethics guidelines. Hepatopancreas tissues were dissected using sterile instruments, immediately frozen in liquid nitrogen to halt metabolic activity, and stored at −80°C for subsequent biochemical and metabolomic analyses. Care was taken to complete dissections within 5 min per individual to minimize post-mortem metabolic changes.

This study was approved by the Ethics Committee of the Laboratory Animal Center at the Zhejiang Institute of Freshwater Fisheries (Approval No. ZIFF-2025-015). All procedures were performed in accordance with the ARRIVE guidelines (Animal Research: Reporting of In Vivo Experiments). Specifically, the following ARRIVE Essential 10 items were addressed in this manuscript: study design (experimental groups, replicates, and control conditions are detailed in Section 2.2); sample size (number of animals per group and total number used are specified in Section 2.1 and Section 2.2); inclusion and exclusion criteria (no animals were excluded; all criteria are described in Section 2.1 and Section 2.2); randomisation (animals were randomly assigned to treatments as described in Section 2.1); blinding (group allocation and outcome assessments are described in Section 2.3); outcome measures (defined in Section 2.4 and Section 2.5); statistical methods (detailed in Section 2.6); animal care and monitoring (acclimation and housing conditions are described in Section 2.1); and interpretation of results (discussed in Section 4).

### 2.4. Enzyme Activity Assays

Hepatopancreas tissue was homogenized in ice-cold physiological saline (0.86% NaCl, *w/v*) at 1:9 (*w/v*) using a motor-driven grinder, and centrifuged at 3000 rpm for 10 min at 4 °C. The supernatant was collected and kept on ice for immediate analysis. Protein concentration was determined using the Bradford method with bovine serum albumin as a standard.

Superoxide dismutase (SOD), acid phosphatase (ACP), and alkaline phosphatase (AKP), as well as malondialdehyde (MDA) content, were measured using commercial kits (Nanjing Jiancheng Bioengineering Institute, Nanjing, China). SOD activity was determined by the xanthine oxidase method, with one unit defined as the amount of enzyme inhibiting nitroblue tetrazolium reduction by 50% at 25 °C. ACP and AKP activities were measured using p-nitrophenyl phosphate as substrate at pH 4.6 and pH 10.1, respectively, and expressed as U/g protein. MDA content was determined by the thiobarbituric acid reactive substances (TBARS) method and expressed as nmol/mg protein. All procedures followed the manufacturer’s protocols. Hepatopancreas tissues from 3 randomly selected individuals per replicate tank (3 replicates per treatment) were pooled to form one biological replicate, yielding 3 biological replicates per treatment group (total of 9 individuals per treatment). Three technical replicates were performed for each biological replicate. This pooling strategy was necessary to obtain sufficient tissue mass for the multi-parameter assays. All kits were from the same production batch.

### 2.5. Metabolite Extraction and Profiling

For metabolomic analysis, hepatopancreas tissues from 3 randomly selected individuals per replicate tank (3 replicates per treatment) were pooled to form one biological replicate, yielding 3 biological replicates per treatment group (total of 9 individuals per treatment). Hepatopancreas tissues pooled from 3 individuals per biological replicate (total wet weight approximately 10–30 mg per pool, depending on species and treatment) were subjected to metabolite extraction using a cold methanol–acetonitrile–water (2:2:1, *v/v/v*) system. The extraction volume was scaled proportionally to tissue weight to maintain consistent solvent-to-tissue ratios. Extraction involved grinding with ceramic beads using a high-throughput homogenizer, ultrasonication in an ice-water bath (15 min), and protein precipitation at −20 °C for 1 h. After centrifugation (14,000 rpm, 15 min, 4 °C), the supernatant was vacuum-dried.

The dried extracts were reconstituted in 100 µL of acetonitrile–water (1:1, *v/v*) and analyzed using a Waters Acquity I-Class PLUS ultra-high-performance liquid chromatography system coupled with a Waters Xevo G2-XS quadrupole time-of-flight (QTOF) mass spectrometer. Chromatographic separation was performed on a Waters Acquity UPLC HSS T3 column (2.1 × 100 mm, 1.8 µm) at 40°C with a flow rate of 0.3 mL/min. The mobile phase consisted of (A) 0.1% formic acid in water and (B) 0.1% formic acid in acetonitrile, with a gradient elution program. Mass spectrometric data were acquired in both positive and negative electrospray ionization modes over a mass range of m/z 50–1500.

Raw data were acquired with MassLynx V4.2 and processed using Progenesis QI (version 2.4) for peak picking, alignment, and normalization. Metabolite identification was performed by matching accurate mass (error < 5 ppm) and MS/MS fragmentation against the METLIN database, HMDB, and an in-house aquatic metabolite database. QC samples were injected every 10 injections to monitor instrument performance.

### 2.6. Statistical Analysis

All data are presented as mean ± standard deviation (SD). Survival percentages were arcsine-transformed prior to analysis to meet normality assumptions (Shapiro–Wilk test). Homogeneity of variances was verified using Levene’s test. Differences among treatment groups were assessed using one-way ANOVA followed by Tukey’s HSD post hoc test. For metabolomic data, fold change (FC) was calculated as the ratio of mean normalized peak intensities between the stress group (Group 3 or Group 5) and the control group (Group 1), followed by log_2_ transformation (log_2_FC). Differential metabolites were identified with VIP > 1.0, *p* < 0.05, and FDR < 0.05 (Benjamini–Hochberg). Biological replicates were *n* = 3 for both enzyme activity assays and metabolomic analysis (each pool consisting of hepatopancreas from 3 individuals). For enzyme activity assays, three technical replicates per biological sample were used; for metabolomics, QC samples were injected every 10 injections to monitor instrument performance. Statistical significance was set at *p* < 0.05. All analyses were performed using SPSS 26.0 and R 4.3.1 (MetaboAnalystR package for metabolomics).

## 3. Results

### 3.1. Experiment 1: Effects of Combined Salinity and pH Stress

#### 3.1.1. Survival Rate Under Combined Salinity-pH Stress

**Figure 1:**
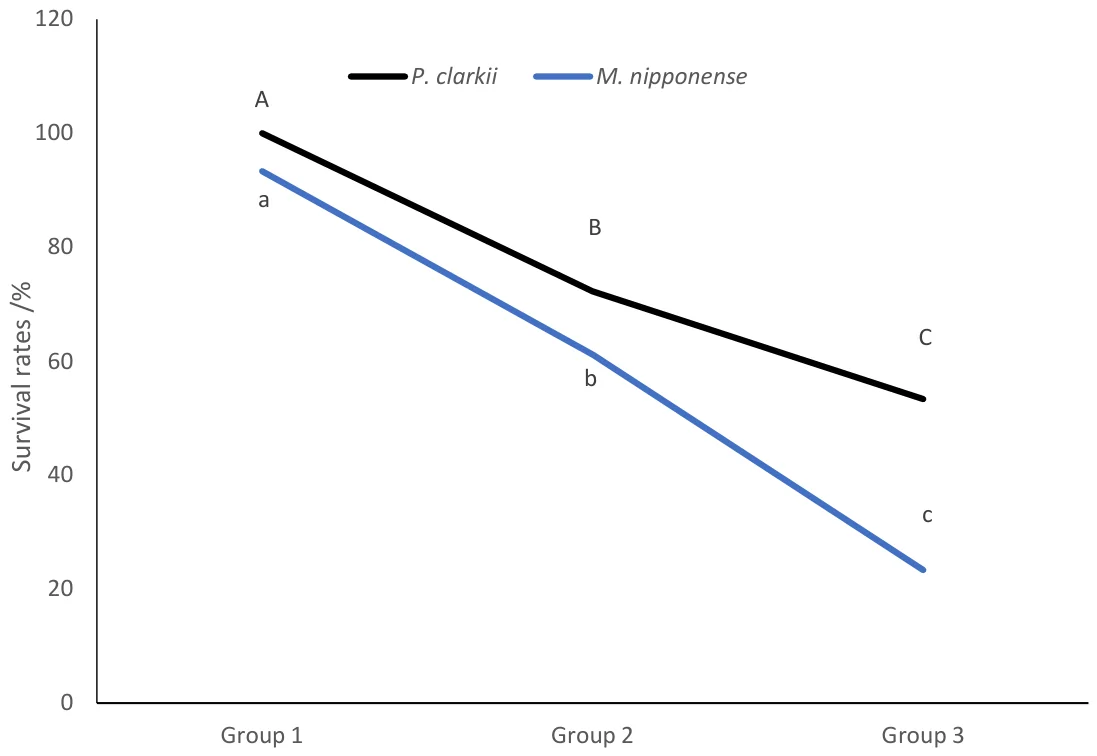
Survival rates of *P. clarkii* and *M. nipponense* exposed to combined salinity and pH stress for 96 h. Data are presented as mean ± SD (*n* = 3). Different uppercase (A, B, C) and lowercase (a, b, c) letters indicate significant differences among groups within the same species (*p* < 0.05).

After 96 h of exposure to combined salinity and pH stress, survival rates of *P. clarkii* in Group 1 (control: 0‰, pH 7.4), Group 2 (15.0‰, pH 9.0), and Group 3 (25.0‰, pH 10.0) were 100.00%, 72.23%, and 53.33%, respectively. For *M. nipponense*, the corresponding survival rates were 93.33%, 61.13%, and 23.33% (Figure 1). In both species, survival followed a dose-dependent decline, with Group 3 significantly lower than Groups 1 and 2 (*p* < 0.05), and Group 2 was significantly lower than Group 1 (*p* < 0.05). *P. clarkii* consistently outperformed *M. nipponense* across all stress levels, with the largest interspecific difference in Group 3 (53.33% vs. 23.33%).

#### 3.1.2. Antioxidant and Metabolic Enzyme Activities

**Figure 2:**
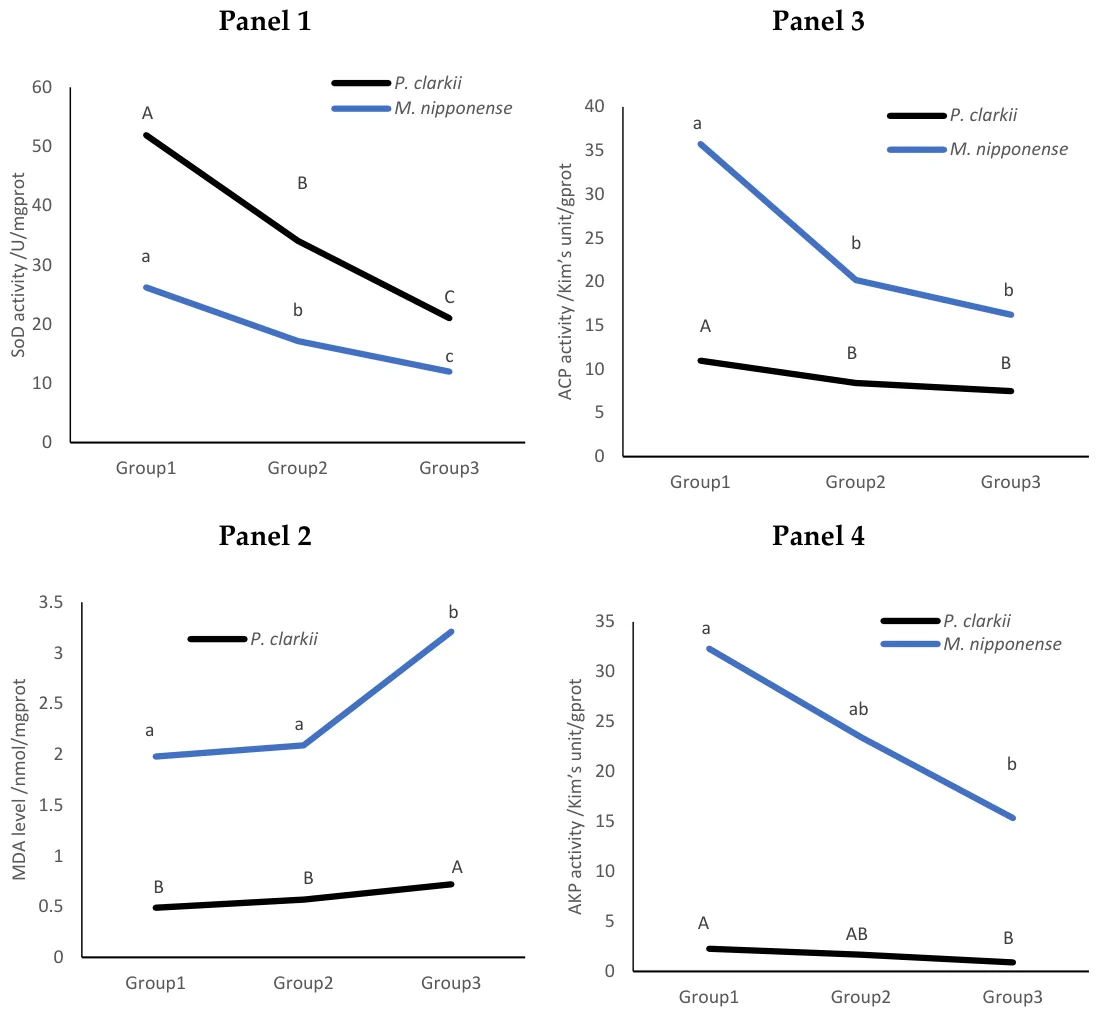
Effects of combined salinity and pH stress on (**Panel 1**) SOD activity, (**Panel 2**) MDA content, (**Panel 3**) ACP activity, and (**Panel 4**) AKP activity in the hepatopancreas of *P. clarkii* and *M. nipponense* after 96 h exposure. Data are presented as mean ± SD (*n* = 3). Different uppercase (A, B, C) letters indicate significant differences among groups within *P. clarkii,* and lowercase (a, b, c) letters indicate significant differences among groups within *M. nipponense* (*p* < 0.05).

In both species, SOD activity decreased while MDA content increased with increasing stress intensity (Figure 2, Panels 1 and 2). *P. clarkii* showed significantly higher SOD activity in Group 1 than in Groups 2 and 3 (*p* < 0.05), with Group 3 exhibiting the lowest activity. Similar trends were observed in *M. nipponense*, with Group 1 showing the highest SOD activity and Group 3 the lowest (*p* < 0.05). MDA levels were significantly elevated in Group 3 relative to Groups 1 and 2 in both species (*p* < 0.05).

ACP and AKP activities declined with increasing stress in both species (Figure 2-Panel 3 and 4). Both in *P. clarkii* and *M. nipponense*, ACP activity was significantly higher in Group 1 than in Groups 2 and 3 (*p* < 0.05); AKP activity in Group 1 was significantly higher than in Group 3 (*p* < 0.05).

Overall, Group 3 exhibited the lowest SOD, ACP, and AKP activities and the highest MDA level in both species, indicating severe oxidative stress and immune suppression.

### 3.2. Experiment 2: Effects of NaHCO_3_ Alkalinity Stress

#### 3.2.1. Survival Rate Under NaHCO_3_ Alkalinity Stress

**Figure 3:**
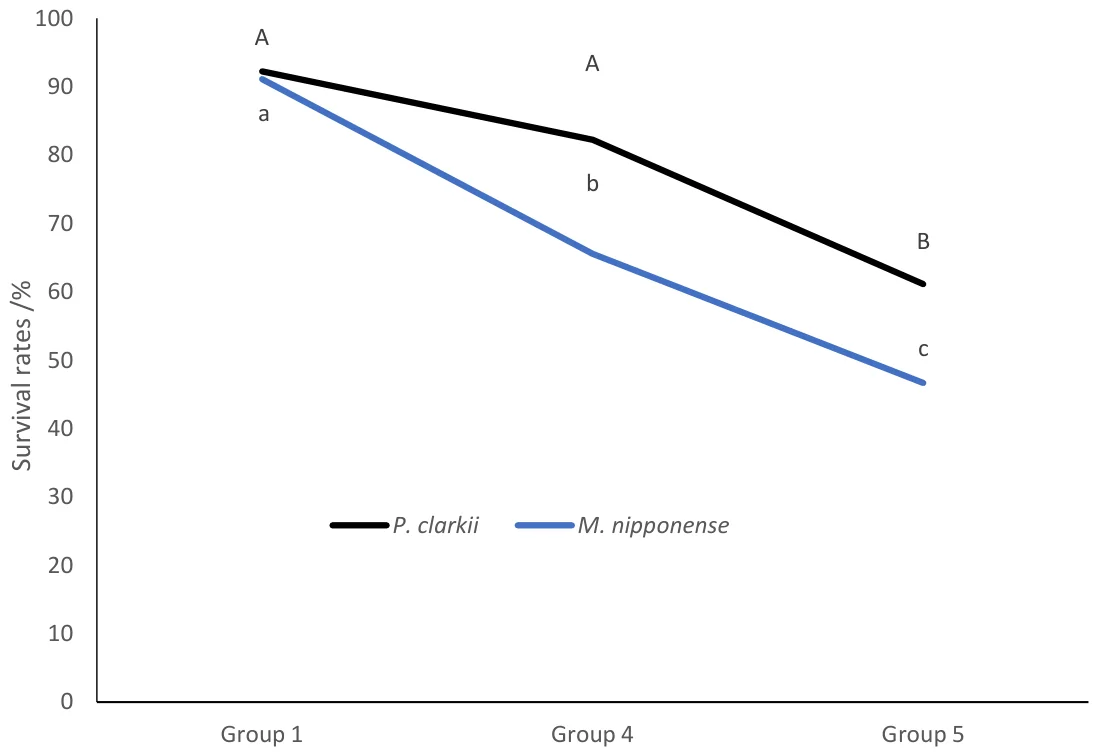
Survival rates of *P. clarkii* and *M. nipponense* exposed to NaHCO_3_ alkalinity stress for 96 h. Data are presented as mean ± SD (*n* = 3). Different uppercase (A, B, C) and lowercase (a, b, c) letters indicate significant differences among groups within the same species (*p* < 0.05).

After 96 h of exposure to NaHCO_3_ alkalinity stress, the survival rates of *P. clarkii* in Group 1 (control: 0 mmol/L), Group 4 (10 mmol/L), and Group 5 (20 mmol/L) were 92.22%, 82.22%, and 61.11%, respectively. For *M. nipponense*, the corresponding survival rates were 91.11%, 65.55%, and 46.67% (Figure 3). In *P. clarkii*, no significant differences were observed among groups (*p* > 0.05), indicating that the tested alkalinity levels did not cause significant mortality in this species. In contrast, for *M. nipponense*, survival in Group 1 was significantly higher than in Groups 4 and 5 (*p* < 0.05), and Group 5 exhibited significantly lower survival than Group 4 (*p* < 0.05).

#### 3.2.2. Antioxidant and Immune-Related Enzyme Activities

**Figure 4:**
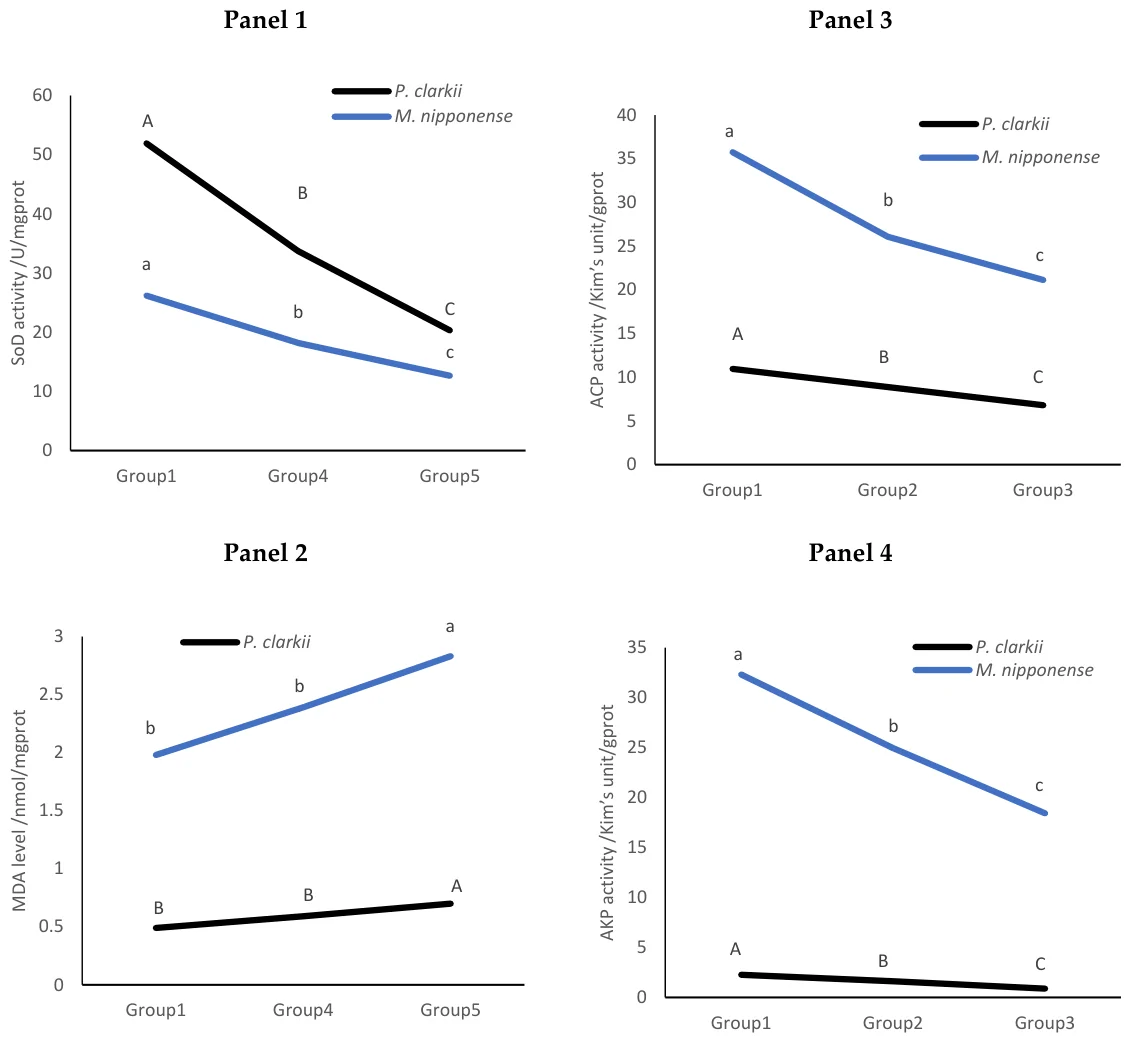
Effects of NaHCO_3_ alkalinity stress on (**Panel 1**) SOD activity, (**Panel 2**) MDA content, (**Panel 3**) ACP activity, and (**Panel 4**) AKP activity in the hepatopancreas of *P. clarkii* and *M. nipponense* after 96 h exposure. Data are presented as mean ± SD (*n =* 3). Different uppercase (A, B, C) letters indicate significant differences among groups within *P. clarkii,* and lowercase (a, b, c) letters indicate significant differences among groups within *M. nipponense* (*p* < 0.05).

In both species, SOD activity decreased while MDA content increased with rising alkalinity (Figure 4, Panels 1 and 2). *P. clarkii* exhibited significantly higher SOD activity in Group 1 than in Groups 4 and 5 (*p* < 0.05), with Group 5 showing the lowest activity. In *M. nipponense*, SOD activity decreased dose-dependently, with Group 1 significantly higher than Groups 4 and 5 (*p* < 0.05), and Group 5 significantly lower than Group 4 (*p* < 0.05). MDA levels were significantly elevated in Group 5 relative to Groups 1 and 4 in *P. clarkii* (*p* < 0.05). In *M. nipponense*, Group 5 exhibited significantly higher MDA than both Group 1 and Group 4 (*p* < 0.05).

ACP and AKP activities also declined with increasing alkalinity (Figure 4, Panels 3 and 4): in *P. clarkii*, both ACP and AKP activities were significantly higher in Group 1 than in Groups 4 and 5 (*p* < 0.05), with Group 5 showing significantly lower activities than Group 4 (*p* < 0.05). Similar trends were observed in *M. nipponense*, with Group 1 showing significantly higher activities than the treated groups (*p* < 0.05), and Group 5 exhibiting the lowest activities (*p* < 0.05).

Overall, Group 5 displayed the lowest SOD, ACP, and AKP activities and the highest MDA level in both species.

### 3.3. Metabolomic Analysis

Metabolomic profiling was performed on hepatopancreas samples from the control group (Group 1) and the high-stress groups (Group 3 from Experiment 1 and Group 5 from Experiment 2) for both species.

#### 3.3.1. Data Quality Assessment and Multivariate Analysis

**Figure 5:**
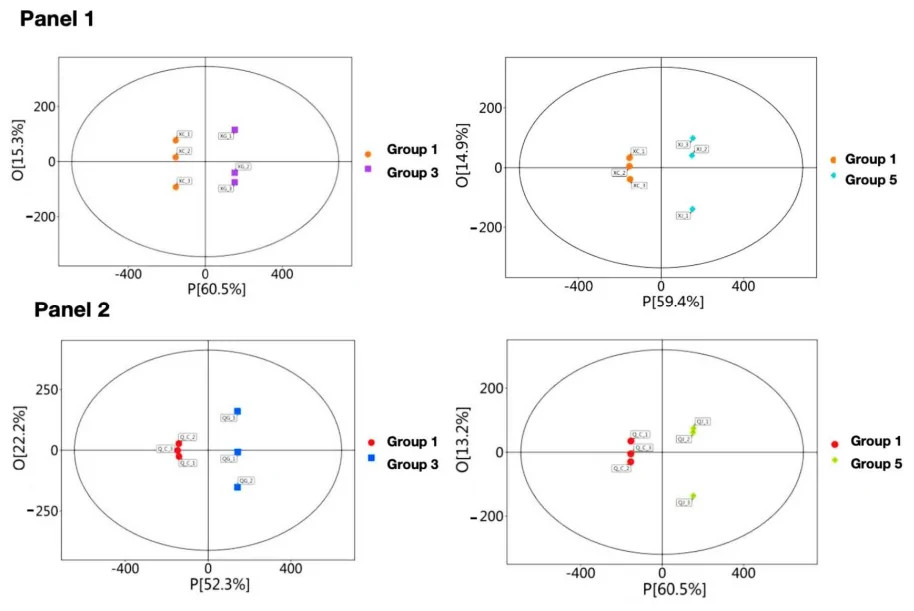
Principal component analysis (PCA) score plots of hepatopancreas metabolomic profiles from *P. clarkii* and *M. nipponense*. (**Panel 1**) Group 1 (control) vs. Group 3 or 5 in the hepatopancreas of *P. clarkii*; (**Panel 2**) Group 1 (control) vs. Group 3 or 5 in the hepatopancreas of *M. nipponense*.

Base peak chromatograms (BPC) from quality control (QC) samples showed excellent overlap with minimal variation in retention time and peak intensity. Principal component analysis (PCA) score plots revealed clear separation between control and stress-exposed samples for both species (Figure 5).

Clear separation between control and stress-exposed samples indicates substantial metabolic reprogramming induced by both stress types.

#### 3.3.2. Differential Metabolites Under Combined Salinity–pH Stress (Group 1 vs. Group 3)

**Figure 6:**
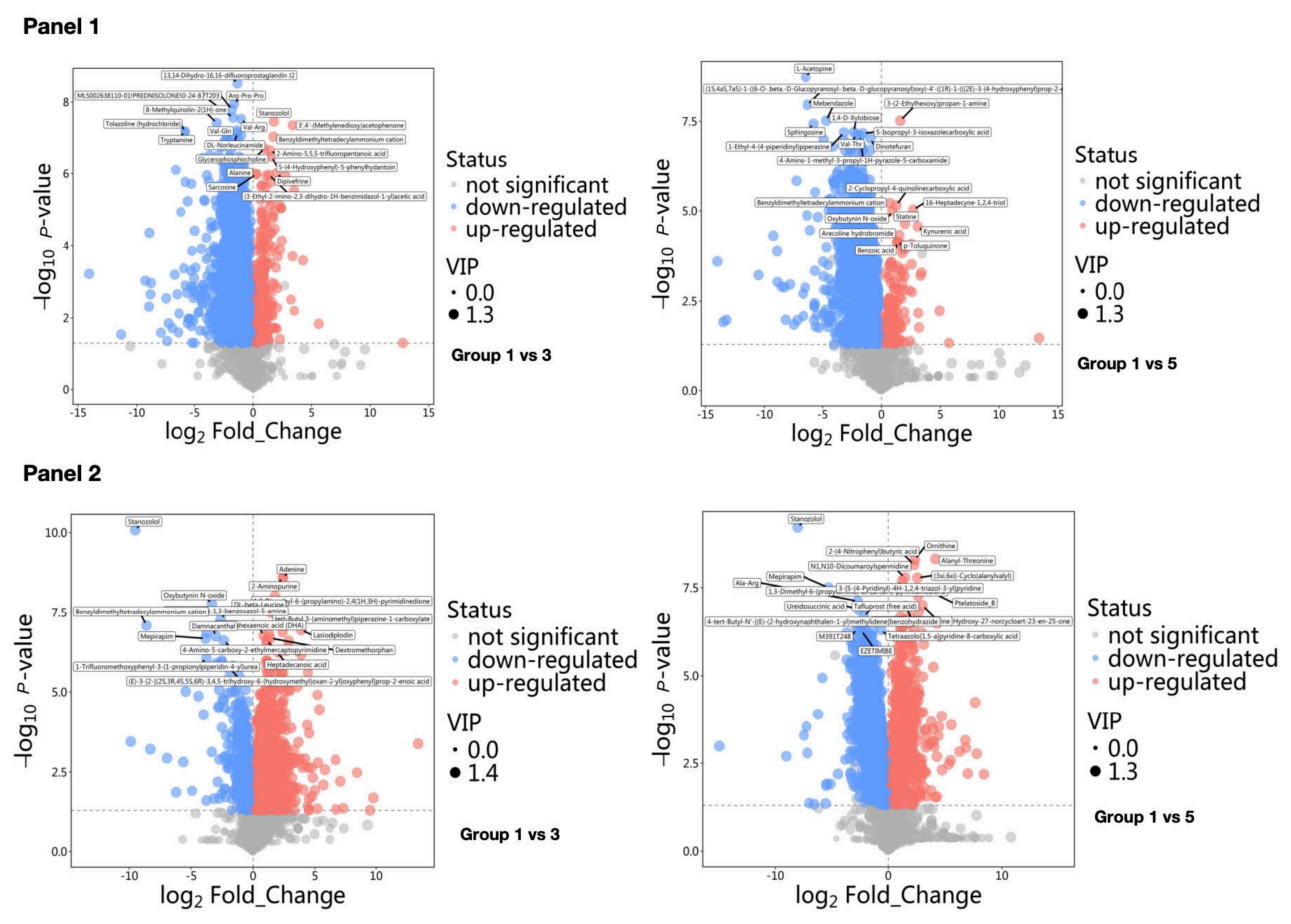
Volcano plots and differential metabolite profiles of hepatopancreas from *P. clarkii* and *M. nipponense*. (**Panel 1**) *P. clarkii*. (**Panel 2**) *M. nipponense*. Red dots represent significantly upregulated metabolites; blue dots represent significantly downregulated metabolites (VIP > 1.0, *p* < 0.05, FDR < 0.05). Key differential metabolites are labeled.

For *P. clarkii*, a total of 23,913 features were detected, of which 3919 were upregulated and 19,994 downregulated in Group 3 relative to Group 1, yielding 1778 significantly differential metabolites (230 upregulated, 1548 downregulated). The most significantly upregulated and downregulated metabolites (log_2_FC > 1.0 or < −1.0) are summarized in Table 1, with upregulated metabolites including glycerophosphoethanolamines, 1-benzopyrans, nitrofurans, nitrophenols, fatty acid esters, beta-lactams, and sulfanilides. Downregulated metabolites included terpene glycosides, eicosanoids, indoles, benzylisoquinolines, yohimbine alkaloids, O-methylated isoflavonoids, and benzoxazines (Figure 6-Panel 1; Table 1).

It should be noted that extremely high log_2_FC values indicate that the metabolite was detected at measurable levels in one group while being near or below the detection limit in the comparator group. Such extreme values reflect qualitative (presence/absence) rather than strictly quantitative differences, and their magnitudes should not be over-interpreted as representing proportional changes in concentration.

For *M. nipponense*, 18,268 features were detected, of which 1342 were significant differential metabolites (1023 upregulated, 319 downregulated). The major differential metabolites are presented in Table 2, including upregulated metabolites such as isoindolines, tropane alkaloids, phenols, stilbenes, terpene glycosides, fatty acid esters, and kavalactones. Downregulated metabolites included glycerophosphocholines, purine ribonucleotides, anthraquinones, eicosanoids, cytochalasans, diterpenoids, and triterpenoids (Figure 6-Panel 2; Table 2).

#### 3.3.3. Differential Metabolites Under NaHCO_3_ Alkalinity Stress (Group 1 vs. Group 5)

For *P. clarkii*, 22,908 features were detected, of which 3024 were upregulated and 19,884 downregulated in Group 5, yielding 1674 significant differential metabolites (153 upregulated, 1521 downregulated). The predominant upregulated and downregulated metabolites are listed in Table 2, with upregulated metabolites including glycerophosphoethanolamines, sulfanilides, fatty alcohols, benzophenones, fatty acid esters, flavones, and glycerophosphoserines. Downregulated metabolites included terpene glycosides, eicosanoids, alpha-methyldeoxybenzoin flavonoids, indoles, steroidal glycosides, phenylacetylindoles, and anthraquinones (Figure 6-Panel 1; Table 3).

For *M. nipponense*, 23,818 features were detected, of which 11,963 were upregulated and 11,755 downregulated in Group 5, resulting in 1613 significant differential metabolites (765 upregulated, 848 downregulated). Key differential metabolites are summarized in Table 4. Upregulated metabolites included glycerophosphoserines, pyrroloindoles, naphthalenes, fatty acid esters, sulfinic acids, flavonoid glycosides, and ceramides. Downregulated metabolites included triterpenoids, sesquiterpenoids, steroidal glycosides, benzoxazines, depsipeptides, O-methylated flavonoids, and diterpenoids (Figure 6-Panel 2; Table 4).

#### 3.3.4. KEGG Pathway Enrichment Analysis

**Figure 7:**
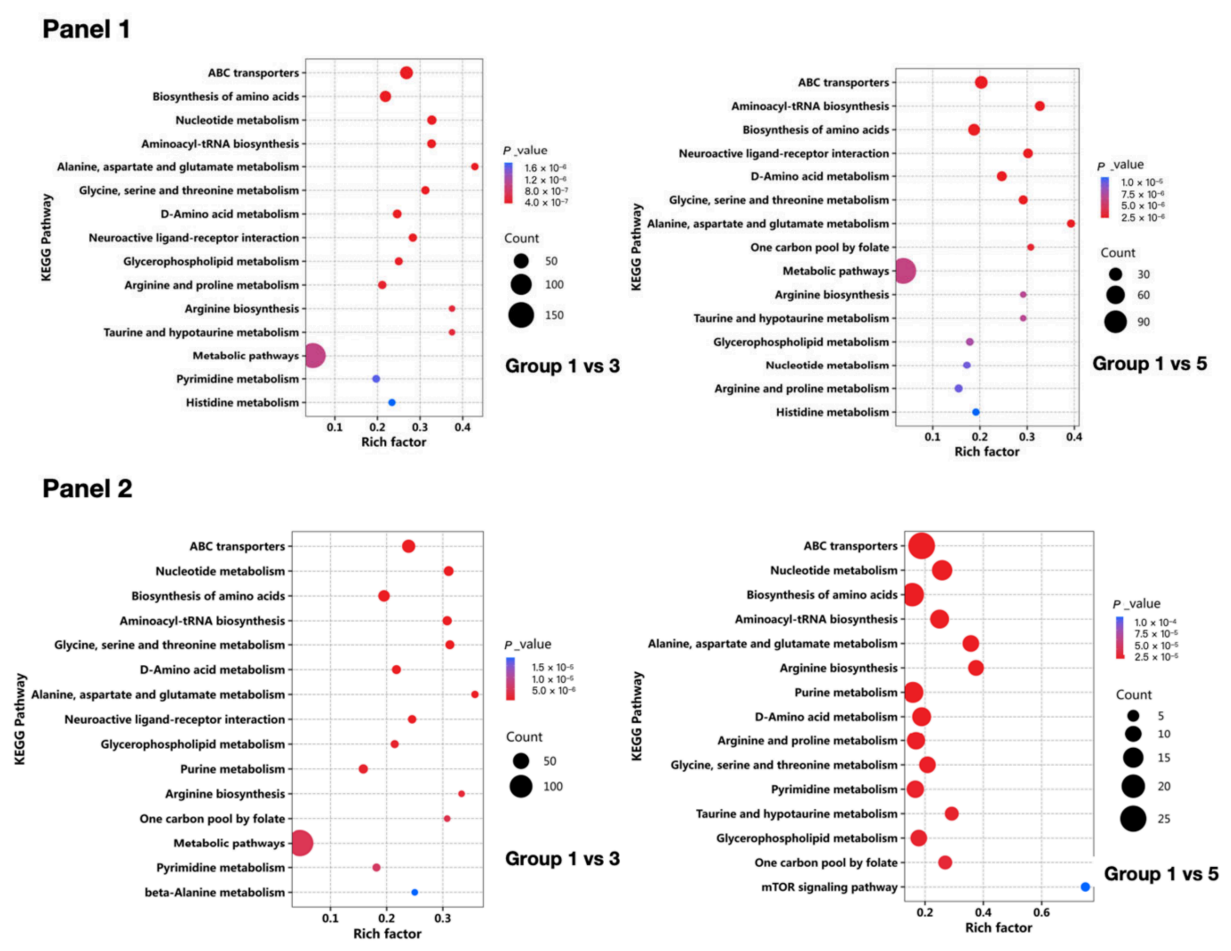
KEGG pathway enrichment analysis of differential metabolites in the hepatopancreas of *P. clarkii* and *M. nipponense*. (**Panel 1**) *P. clarkii*. (**Panel 2**) *M. nipponense*. The size of each bubble represents the number of metabolites enriched in the pathway; the color gradient represents the significance level (−log_10_ *p*-value). Pathways with *p* < 0.05 were considered significantly enriched.

Under combined salinity–pH stress (Group 1 vs. Group 3), both species showed enrichment in similar pathways: metabolic pathways, ABC transporters, biosynthesis of amino acids, nucleotide metabolism, aminoacyl-tRNA biosynthesis, metabolism of alanine, aspartate, glutamate, glycine, serine, threonine, and D-amino acids (Figure 7). For *P. clarkii*, additional enrichment in D-amino acid metabolism and alanine/aspartate/glutamate metabolism was noted (Figure 7-Panel 1: Group 1 vs. 3). For *M. nipponense*, purine and pyrimidine metabolism were also significantly affected (Figure 7-Panel 2: Group 1 vs. 3).

Under NaHCO_3_ stress (Group 1 vs. Group 5), *P. clarkii* exhibited enrichment in metabolic pathways, ABC transporters, aminoacyl-tRNA biosynthesis, biosynthesis of amino acids, neuroactive ligand–receptor interaction, D-amino acid metabolism, and glycine/serine/threonine metabolism (Figure 7—Panel 1: Group 1 vs. 5). *M. nipponense* showed a broader range of enriched pathways, including ABC transporters, nucleotide metabolism, amino acid biosynthesis and metabolism, purine/pyrimidine metabolism, taurine and hypotaurine metabolism, and glycerophospholipid metabolism (Figure 7—Panel 2: Group 1 vs. 5).

## 4. Discussion

### 4.1. Survival Responses to Combined Salinity–pH Stress and NaHCO_3_ Alkalinity Stress

This study compared the survival responses of juvenile *P. clarkii* and juvenile *M. nipponense* under combined salinity–pH stress and NaHCO_3_ alkalinity stress, and aimed to identify the physiological and metabolic mechanisms underlying any such differences. Our results clearly reject the null hypothesis: *P. clarkii* exhibited consistently higher survival than *M. nipponense* across all stress conditions, with the largest interspecific difference under combined salinity–pH stress (53.33% vs. 23.33%). Salinity, pH, and alkalinity are critical parameters influencing aquatic animal survival in saline–alkaline environments [17]. Our finding that combined high salinity (25.0‰) and high pH (10.0) reduced *P. clarkii* survival to 53.33% aligns with Tao et al. [8], who reported a 96 h LC_50_ of pH 10.194 for this species. The higher tolerance of *P. clarkii* may reflect its evolutionary history as an invasive species capable of colonizing brackish waters [7], with well-developed osmoregulatory mechanisms. In contrast, *M. nipponense* is primarily a freshwater species with limited osmoregulatory capacity; studies on the selectively bred “Taihu No. 3” strain showed a 96 h LC_50_ salinity of 11.841‰ under isolated salinity stress [3]. The combined effect with high pH likely imposes synergistic challenges that exceed its adaptive capacity. Simultaneous elevation of salinity and pH creates a dual burden: hyperosmotic stress demands increased ion transport, while alkaline pH disrupts ammonia excretion and acid–base regulation [8].

Regarding alkalinity stress, *P. clarkii* tolerated up to 20 mmol/L NaHCO_3_ with 61.11% survival, whereas *M. nipponense* survival dropped to 46.67%. The 96 h LC_50_ for carbonate alkalinity in *P. clarkii* juveniles has been reported as 16.03 mmol/L [7]. For *M. nipponense,* Jin et al. [16] reported a 96 h LC_50_ of 14.42 mmol/L and a safe concentration of 4.71 mmol/L. This species difference likely stems from distinct branchial ion transport mechanisms and acid–base regulation capacities [7,18]. For *P. clarkii*, combined salinity–pH stress caused greater mortality than NaHCO_3_ alkalinity alone, suggesting that the concurrent elevation of salinity and pH imposes a greater physiological burden than alkalinity alone. High salinity imposes osmotic challenges requiring substantial energy for ion regulation [19,20], while high pH shifts ammonia toward toxic NH_3_. However, it should be noted that our experimental design (simultaneous manipulation of salinity and pH) does not allow rigorous demonstration of synergy; a full factorial design would be required to formally test for synergistic interactions. For *M. nipponense*, both stress types caused severe mortality at high intensities, suggesting that even isolated alkalinity stress at 20 mmol/L exceeds its physiological limits.

From an aquaculture perspective, *P. clarkii* is a more suitable candidate for moderately high saline–alkaline waters, with a recommended threshold of salinity ≤ 15.0‰, pH ≤ 9.0, and NaHCO_3_ alkalinity ≤ 10 mmol/L. In contrast, *M. nipponense* should be restricted to low-alkalinity freshwater (alkalinity < 5 mmol/L) with rigorous monitoring.

### 4.2. Physiological and Biochemical Responses

The marked differences in survival between the two species prompted us to investigate the underlying physiological mechanisms. We therefore examined antioxidant and immune-related enzyme activities to determine whether differential oxidative stress responses and immune competence could explain the observed survival patterns.

#### 4.2.1. Antioxidant Responses (SOD and MDA)

Both species exhibited similar patterns: SOD activity decreased while MDA content increased with stress intensity. In *P. clarkii*, SOD activity was significantly higher in controls than in stress groups; in *M. nipponense*, the same trends were observed, but the magnitude of change was greater.

SOD is the primary antioxidant enzyme that converts superoxide radicals to H_2_O_2_ and O_2_ [21], while MDA indicates membrane lipid peroxidation [8]. The inverse relationship between SOD and MDA indicates that combined salinity–pH stress and NaHCO3 alkalinity stress overwhelm the antioxidant system. Our results are consistent with previous studies [3,8,22]. The more pronounced oxidative damage in *M. nipponense* aligns with its lower survival and suggests less robust antioxidant capacity.

#### 4.2.2. Immune-Related Enzyme Responses (ACP and AKP)

ACP and AKP are lysosomal enzymes critical for crustacean immunity, participating in intracellular digestion and pathogen degradation [22,23]. In both species, ACP and AKP activities declined significantly under severe stress, indicating immunosuppression. For *P. clarkii*, ACP and AKP activities were significantly higher in controls than in stress groups. For *M. nipponense*, both enzymes showed dose-dependent decreases, with Group 5 exhibiting the lowest activities. This suppression suggests energy reallocation from immune maintenance to immediate survival functions (osmoregulation, acid–base balance) under severe stress [23,24]. The greater immune suppression in *M. nipponense* likely contributes to its higher mortality.

#### 4.2.3. Integrated Interpretation

Under moderate stress, both species mounted compensatory responses. Under severe stress, however, antioxidant and immune systems were overwhelmed. The similarity in physiological response patterns suggests common stress mechanisms: enhanced ROS production from disrupted mitochondrial electron transport [21,25], leading to lipid peroxidation and immune suppression. *P. clarkii* consistently exhibited less severe disruption, reinforcing its greater tolerance.

### 4.3. Metabolomic Responses

While enzyme activities provided valuable insights into physiological stress responses, they represent only one level of biological organization. To gain a more comprehensive understanding of species-specific stress adaptation, we next examined the hepatopancreas metabolome, which captures the integrated outcome of multiple regulatory pathways and provides direct molecular evidence of metabolic reprogramming. Metabolomic profiling revealed species-specific and stress-specific metabolic reprogramming. For *P. clarkii*, combined salinity–pH stress upregulated glycerophosphoethanolamines, fatty acid esters, and beta-lactams, and downregulated terpene glycosides, eicosanoids, and indoles. Under NaHCO_3_ stress, upregulation included flavones and glycerophosphoserines, with downregulation of terpene glycosides and steroidal glycosides. For *M. nipponense*, combined salinity–pH stress upregulated isoindolines, tropane alkaloids, phenols, stilbenes, terpene glycosides, fatty acid esters, and kavalactones, and downregulated glycerophosphocholines, purine ribonucleotides, anthraquinones, eicosanoids, cytochalasans, diterpenoids, and triterpenoids. Under NaHCO_3_ stress, upregulation occurred for glycerophosphoserines, pyrroloindoles, naphthalenes, fatty acid esters, sulfinic acids, flavonoid glycosides, and ceramides, while triterpenoids, sesquiterpenoids, steroidal glycosides, benzoxazines, depsipeptides, O-methylated flavonoids, and diterpenoids were downregulated.

#### 4.3.1. Common Metabolic Responses

Both species shared upregulation of fatty acid esters under both stress types. Fatty acid esters (particularly FAHFAs) possess anti-inflammatory and antioxidant properties [26,27], suggesting an adaptive response to mitigate oxidative damage. Downregulation of eicosanoids and terpene glycosides was also common. Eicosanoids mediate inflammation and immunity [28,29]; their suppression aligns with reduced ACP/AKP activities, indicating coordinated immune downregulation. Terpene glycosides, involved in detoxification [30], were reduced, suggesting impaired glycosylation capacity.

#### 4.3.2. Species-Specific Metabolic Signatures

*P. clarkii* uniquely upregulated beta-lactams under salinity–pH stress, potentially reflecting stress-induced alterations in the microbiome or endogenous defense [31,32], and downregulated yohimbine alkaloids and O-methylated isoflavonoids [33,34], indicating suppression of protective secondary metabolism.

*M. nipponense* uniquely upregulated tropane alkaloids and pyrroloindoles. Tropane alkaloids are neuromodulators with anticholinergic activity [35]; Pyrroloindoles have neuroprotective and antimicrobial activities [36,37], potentially defending against alkalinity-induced damage. Notably, glycerophosphocholine (GPC) depletion occurred specifically in *M. nipponense* under combined stress. GPC is a critical organic osmolyte protecting cells against hypertonicity [38]. Its depletion likely contributes to osmoregulatory failure.

#### 4.3.3. Pathway Enrichment and Mechanistic Implications

KEGG analysis revealed enrichment in metabolic pathways, ABC transporters, amino acid biosynthesis, and aminoacyl-tRNA biosynthesis in both species, indicating profound effects on energy metabolism, amino acid homeostasis, and protein synthesis. ABC transporters mediate transmembrane transport of ions and xenobiotics, likely playing key roles in osmotic adaptation.

ABC transporters facilitate transmembrane transport of ions, amino acids, and xenobiotics; their upregulation may alleviate osmotic and ionic burdens by excreting excess ions and toxic metabolites under combined salinity–pH stress and NaHCO_3_ alkalinity stress [39]. Concurrent enrichment of amino acid metabolism—particularly alanine, glutamate, and glycine—suggests these amino acids serve as energy sources or osmoprotectants to maintain balance and redox homeostasis [40]. In *M. nipponense*, the extensive metabolic reprogramming aligns with transcriptomic findings in fatty acid biosynthesis, insulin signaling, and AMPK signaling [41], confirming that energy regulation and membrane remodeling are central to saline–alkaline acclimation. Additionally, several compound classes identified (cytochalasans, pyrroloindoles, tropane alkaloids) are typically of microbial origin [42,43], and their presence may reflect gut microbiota shifts in response to host stress, as alkalinity exposure significantly alters intestinal microbiota composition in *M. nipponense* [31].

### 4.4. Comparative Summary and Aquaculture Implications

*P. clarkii* exhibits significantly higher tolerance to both combined salinity–pH stress and NaHCO_3_ alkalinity stress than *M. nipponense*, as evidenced by higher survival, less severe oxidative damage and immune suppression, and more targeted metabolomic reprogramming. For practical cultivation, *P. clarkii* can be considered for saline–alkaline waters with salinity up to 15.0‰ and pH up to 9.0, and NaHCO_3_ alkalinity up to 10 mmol/L (with gradual acclimation). In contrast, *M. nipponense* should be cultured only in waters with alkalinity below 5 mmol/L (safe concentration 4.71 mmol/L [15]) and salinity below 15.0‰, with rigorous monitoring. The metabolomic biomarkers identified—particularly GPC depletion and ceramide accumulation in *M. nipponense*, and glycerophosphoethanolamine upregulation in *P. clarkii*—offer potential tools for non-invasive stress monitoring. Future research should include chronic exposure studies, factorial designs, transcriptomic/proteomic analyses, and field validation.

### 4.5. Limitations and Future Perspectives

Several limitations should be acknowledged. First, the 96 h exposure represents acute stress and may not reflect long-term adaptation; chronic studies are needed to evaluate effects on growth and reproduction. Second, commercial sea salt may not fully replicate natural saline–alkaline ionic composition, and the combined salinity–pH design does not allow separation of individual factor effects or formal testing of synergy; full factorial designs would be required to disentangle these interactions. Third, metabolomic profiling was performed only on hepatopancreas, and findings were not validated at the transcript or protein level; future studies should include gill tissue and qPCR or Western blot to confirm the regulatory mechanisms. Fourth, the untargeted metabolomics approach is inherently semi-quantitative. Extremely high log_2_FC values (e.g., >10) indicate that a metabolite was detectable in one group but near or below the detection limit in the comparator group, representing qualitative rather than strictly quantitative differences. Targeted quantification using authentic standards would be needed for precise measurements. Fifth, pooling tissues from three individuals per biological replicate was necessary for sufficient sample mass but may obscure inter-individual variation. Sixth, the non-linear gradient spacing (0, 15, 25‰; pH 7.4, 9.0, 10.0) limits threshold identification; finer gradients would provide more detailed dose–response relationships. Finally, the two species differed in absolute body size due to inherent growth characteristics; although individuals were at the same developmental stage, size differences may still influence certain physiological parameters. Despite these limitations, this study provides the first comparative physiological and metabolomic foundation for species selection in saline–alkaline aquaculture.

## 5. Conclusions

This study provides a comparative assessment of combined salinity–pH stress and NaHCO_3_ alkalinity stress responses in juvenile *P. clarkii* and juvenile *M. nipponense* through integrated analyses of survival, enzyme activities, and metabolomics. The main conclusions are:

1. *P. clarkii* exhibited substantially higher tolerance than *M. nipponense* under both combined salinity–pH stress (25.0‰/pH 10.0: 53.33% vs. 23.33%) and NaHCO_3_ alkalinity stress (20 mmol/L: 61.11% vs. 46.67%).
2. Severe stress induced oxidative damage (decreased SOD, increased MDA) and immunosuppression (decreased ACP, AKP) in both species, with more pronounced effects in *M. nipponense*.
3. Metabolomics revealed species-specific reprogramming: *P. clarkii* upregulated protective lipids (glycerophosphoethanolamines, fatty acid esters, flavones), whereas *M. nipponense* showed depletion of glycerophosphocholines and accumulation of ceramides—novel molecular signatures of vulnerability.
4. KEGG analysis confirmed energy metabolism, amino acid homeostasis, ABC transporters, and protein synthesis as primary affected pathways, with broader disruption in *M. nipponense*.
5. For aquaculture, *P. clarkii* is recommended for moderately high saline–alkaline waters (salinity ≤ 15.0‰, pH ≤ 9.0, alkalinity ≤ 10 mmol/L), while *M. nipponense* should be restricted to low-alkalinity freshwater (<5 mmol/L) with rigorous monitoring.
6. The identified metabolite biomarkers offer potential targets for selective breeding and non-invasive stress monitoring. However, chronic and field validation studies are needed to confirm long-term applicability.

## Figures and Tables

**Table 1:** Major differential metabolites in the hepatopancreas of *P. clarkii* under stress (Group 1 vs. Group 3).

Upregulated Metabolites	Log Fold Change	Downregulated Metabolites	Log Fold Change
Glycerophosphoethanolamines (GPE)	12.80	Terpene glycosides	−7.39
1-benzopyrans	4.27	Eicosanoids	−6.19
Nitrofurans	2.10	Indoles	−5.31
Nitrophenols	2.08	Benzylisoquinolines	−5.22
Fatty acid esters	1.57	Yohimbine alkaloids	−5.21
Beta-lactams	1.47	O-methylated isoflavonoids	−4.52
Sulfanilides	1.31	Benzoxazines	−3.98

**Table 2:** Major differential metabolites in the hepatopancreas of *M. nipponense* under stress (Group 1 vs. Group 3).

Upregulated Metabolites	Log Fold Change	Downregulated Metabolites	Log Fold Change
Isoindolines	9.77	Glycerophosphocholines	−5.64
Tropane alkaloids	5.68	Purine ribonucleotides	−3.15
Phenols	5.18	Anthraquinones	−3.01
Stilbenes	4.99	Eicosanoids	−2.36
Terpene glycosides	4.16	Cytochalasans	−1.65
Fatty acid esters	2.92	Diterpenoids	−1.44
Kavalactones	0.38	Triterpenoids	−1.29

**Table 3:** Major differential metabolites in the hepatopancreas of *P. clarkii* under stress (Group 1 vs. Group 5).

Upregulated Metabolites	Log Fold Change	Downregulated Metabolites	Log Fold Change
Glycerophosphoethanolamines	13.38	Terpene glycosides	−8.88
Sulfanilides	3.19	Eicosanoids	−8.53
Fatty alcohols	2.70	Alpha-methyldeoxybenzoin flavonoids	−8.30
Benzophenones	1.70	Indoles	−7.27
Fatty acid esters	1.54	Steroidal glycosides	−4.84
Flavones	1.36	Phenylacetylindoles	−4.73
Glycerophosphoserines	1.26	Anthraquinones	−3.78

**Table 4:** Major differential metabolites in the hepatopancreas of *M. nipponense* under stress (Group 1 vs. Group 5).

Upregulated Metabolites	Log Fold Change	Downregulated Metabolites	Log Fold Change
Glycerophosphoserines	8.42	Triterpenoids	−7.15
Pyrroloindoles	7.78	Sesquiterpenoids	−5.51
Naphthalenes	3.97	Steroidal glycosides	−3.79
Fatty acid esters	3.22	Benzoxazines	−3.65
Sulfinic acids	2.97	Depsipeptides	−3.61
Flavonoid glycosides	2.97	O-methylated flavonoids	−3.30
Ceramides	2.43	Diterpenoids	−3.21

## Data Availability

The processed LC-MS metabolomics data supporting the findings of this study are available within the article and its Supplementary Materials. The raw LC-MS files are available from the corresponding author upon reasonable request.

## Supplementary Materials

The following supporting information can be downloaded at: https://www.mdpi.com/article/10.3390/ani16172778/s1.

## References

[1] Mu F., Cheng Y.X., Wu X.G. (2007). Distribution and industrial development of crayfish in the world. J. Shanghai Ocean Univ..

[2] Chen P.C., Shih C.H., Chu T.J., Lee Y.C., Tzeng T.D. (2017). Phylogeography and genetic structure of the oriental river prawn *Macrobrachium nipponense* (Crustacea: Decapoda: Palaemonidae) in East Asia. PLoS ONE.

[3] Zhou R., Xu M.J., Zhang W.Y., Xiong Y.W., Fu H.T., Qiao H., Jiang S.F., Jin S.B. (2025). Effect of salinity exposure on the antioxidant system of “Taihu No. 3” *Macrobrachium nipponense*. Sci. Rep..

[4] Huang Y.Q., Gao P.C., Yu D.D., Sun Z., Yang X., Lai Q.F., Chi H. (2025). A comparative analysis on the biochemical composition and nutrition evaluation of crayfish (*Procambarus clarkii*) cultivated in saline-alkali and fresh water. Foods.

[5] Wang P.P., Bakari J.S., Han Y.Q., Hu H.H., Liu Z.L., Zhang Y.W., Chen Z.G., Huang C.G., Wang M.M., Chen H.G. (2026). Effects of thermal stress on growth and reproduction of *Procambarus clarkii* and aquaculture best practices. Animals.

[6] Colin J.B., Richard J.G., Jonathan M.W. (2012). Extreme environments: Hypersaline, alkaline, and ion-poor waters. Fish Physiology.

[7] Guo K., Zhao Z.G., Zhang R., Luo L., Wang S.H., Xu W. (2025). Integrated untargeted metabolomics and physiological analysis reveal the response mechanisms of *Procambarus clarkii* larvae to carbonate alkalinity exposure. Comparative Biochemistry and Physiology. Toxicology & pharmacology: CBP.

[8] Tao Y.F., Qiang J., Wang H., Xu P., Ma X.Y., Zhao W.Q. (2016). Acute toxicity of high pH stress and its effect on enzymes activity and histological structure of gill and hepatopancreas in *Procambarus clarkii*. J. Fish. China.

[9] Yu H., Zou S.B., Yuan H.W., Liu M., Ni M., Yuan J.L. (2025). Physiology and molecular response mechanisms in the gills of *Macrobrachium rosenbergii* under acute NaHCO_3_ alkaline stress. Antioxidants.

[10] González-Vera C., Brown J.H. (2017). Effects of alkalinity and total hardness on growth and survival of postlarvae freshwater prawns, *Macrobrachium rosenbergii* (De Man 1879). Aquaculture.

[11] Li Y.M., Ma J.L., Ye Y.C., Yao Z.L., Gao P.C., Zhou K., Zhao Y.L., Lai Q.F. (2025). Acute alkaline stress activates glucose metabolism for energy supply and induces the immune response in the oriental river prawn, *Macrobrachium nipponense*. Mar. Biotechnol..

[12] Jin S., Xu M., Gao X., Jiang S., Xiong Y., Zhang W., Qiao H., Wu Y., Fu H. (2024). Effects of alkalinity exposure on antioxidant status, metabolic function, and immune response in the hepatopancreas of *Macrobrachium nipponense*. Antioxidants.

[13] Fan Y., Feng J., Xie N., Ling F., Wang Z., Ma K., Hua X., Li J. (2022). RNA-seq provides novel insights into response to acute salinity stress in oriental river prawn *Macrobrachium nipponense*. Mar. Biotechnol..

[14] Bao L. (2000). The Effects of pH, Salinity, and Dietary Phosphorus on the Growth of *Macrobrachium nipponense*. Master’s Thesis.

[15] Jin S.B., Zhou R., Gao X.B., Xiong Y.W., Zhang W.Y., Qiao H., Wu Y., Jiang S.F., Fu H.T. (2024). Identification of the effects of alkalinity exposure on the gills of oriental river prawns, *Macrobrachium nipponense*. BMC Genom..

[16] Jin S.B., Zhang Y.F., Fu H.T., Zhang W.Y., Qiao H., Xiong Y.W., Jiang S.F. (2025). Transcriptome profiling analysis reveals changes in the antioxidant defense system, morphology, and gene expression in the gills of *Macrobrachium nipponense* caused by alkalinity exposure. Int. J. Mol. Sci..

[17] Ding S., Wang F., Guo B., Li X.S. (2008). Effects of salinity fluctuation on the molt, growth, and energy budget of juvenile *Fenneropenaeus chinensis*. Chin. J. Appl. Ecol..

[18] Chen Z.Y., Zhu S.H., Feng B.B., Zhang M., Gong J.H., Chen H.G., Munganga B.P., Tao X.J., Feng J.B. (2024). Temporal transcriptomic profiling reveals dynamic changes in gene expression of giant freshwater prawn upon acute saline-alkaline stresses. Mar. Biotechnol..

[19] Austin C.M. (1995). Effect of temperature and salinity on the survival and growth of juvenile redclaw (*Cherax quadricarinatus*). Freshw. Crayfish.

[20] Rida R., ZeinEddine R., Kreydiyyeh S., Garza D.Y.A., Saoud I.P. (2020). Influence of salinity on survival, growth, hemolymph osmolality, gill sodium potassium ATPase activity, and sodium potassium chloride co-transporter expression in the redclaw crayfish *Cherax quadricarinatus*. J. World Aquac. Soc..

[21] Qiao Y.B., Li S.S., Cui W.B., Zuo R.T., Chang Y.Q. (2020). Effects of acute salinity on antioxidation and non-specific immunity of *Procambarus clarkia*. Chin. Fish. Qual. Stand..

[22] Che C.X., Li Y.T., Qin K.X., Fan Z.W., Li W.J., Gao S., Yang P., Wang C.L., Mu C.K.G., Wang H. (2024). Effects of bicarbonate on antioxidant, immune, osmolytic and metabolic capabilities of *Scylla paramamosain*. Aquaculture.

[23] Liu S.Q., Jiang X.L., Mou H.J., Wang H.M., Guan H.S. (1999). Effects of immunopoiysaccharide on LSZ, ALP, ACP and POD activities of *Penaeus chinensis* serum. Oceanol. Limnol. Sin..

[24] Zhao Y.C., Wang R.J., Shen M., Cui Y.T., Wang S.S., Li Y.Q., Fu R.J., Zhang S.L. (2019). Effects of high-salt stress on daily weight gain, osmoregulation and immune related enzyme activities in *Litopenaeus vannamei* postlarvae. J. Fish. China.

[25] Lu Y.P., Qian K., Wang L., Zhang X.X., Wang D.M., Li J.T., Xian J.A., Wang A.L. (2019). Effect of salinities on activities of antioxidant enzymes and immune-ralated enzymes of the white shrimp *Litopenaeus vannamei*. Hebei Fish..

[26] Li L., Wang P., Jiao X., Qin S., Liu Z., Ye Y., Song Y., Hou H. (2024). Fatty acid esters of hydroxy fatty acids: A potential treatment for obesity-related diseases. Obes. Rev..

[27] Olajide T.M., Cao W.M. (2022). Exploring foods as natural sources of FAHFAs—A review of occurrence, extraction, analytical techniques and emerging bioactive potential. Trends Food Sci. Technol..

[28] Dennis E.A., Norris P.C. (2015). Eicosanoid storm in infection and inflammation. Nat. Rev. Immunol..

[29] Heckmann L.H., Sibly R.M., Timmermans M.J., Callaghan A. (2008). Outlining eicosanoid biosynthesis in the crustacean Daphnia. Front. Zool..

[30] Kurze E., Wüst M., Liao J., Mcgraphery K., Hoffmann T., Song C., Schwab W. (2022). Structure–function relationship of terpenoid glycosyltransferases from plants. Nat. Prod. Rep..

[31] Kryukova D., Sokolov A., Maksimov M. (2020). Pharmacology of inhibitor-protected beta-lactam agents. Novejshie Zarubezhnye I Otechestvennye Lek. Prep. Farmakoter. Farmakodinamika Farmakokinet..

[32] Theyagarajan K., Sruthi V.P., Mohanapriya D., Thenmozhi K., Senthilkumar S. (2024). Biosensor platform for testing active pharmaceutical ingredients. Health and Environmental Applications of Biosensing Technologies.

[33] Hall L.W., Trim C.M. (2016). Principles of sedation, anticholinergic agents, and principles of premedication. Veterinary Anaesthesia.

[34] Polturak G., Misra R.C., El Demerdash A., Owen C., Steed A., McDonald H.P., Wang J.J., Saalbach G., Martins C., Chartrain L. (2023). Discovery of isoflavone phytoalexins in wheat reveals an alternative route to isoflavonoid biosynthesis. Nat. Commun..

[35] Kyu H.S., Min J.K., Niti S., Seong S.A.A. (2022). Beauty of the beast: Anticholinergic tropane alkaloids in therapeutics. Nat. Prod. Bioprospecting.

[36] Sun C.H., Tian W.Y., Lin Z., Qu X.D. (2022). Biosynthesis of pyrroloindoline-containing natural products. Nat. Prod. Rep..

[37] Tian W.Y., Sun C.H., Zheng M., Harmer J.R., Yu M.J., Zhang Y.N., Peng H.D., Zhu D.Q., Deng Z.X., Chen S.L. (2018). Efficient biosynthesis of heterodimeric C^3^-aryl pyrroloindoline alkaloids. Nat. Commun..

[38] Rosas R.J.A., Valenzuela S.E.M. (2010). Enzymes involved in osmolyte synthesis: How does oxidative stress affect osmoregulation in renal cells?. Life Sci..

[39] Li Y.L., He Y.Y., Wang Q., Guan C.H., Zhou Y.J., Li Z.X. (2024). Analysis of expression patterns of *PcABCG5* Gene in *Penaeus chinensis* under saline-alkali stress. Prog. Fish. Sci..

[40] Duan Y.F., Xing Y.F., Zhu X.Y., Li H., Wang Y., Nan Y.X. (2023). Integration of transcriptomic and metabolomic reveals carbonate alkalinity stress responses in the hepatopancreas of *Litopenaeus vannamei*. Aquat. Toxicol..

[41] Jin S., Fu H., Xiong Y., Qiao H., Zhang W., Jiang S. (2025). Identification of the changes of immune response, morphology, and gene expressions in hepatopancreas of *Macrobrachium nipponense* under the treatment of alkalinity exposure. Comp. Biochem. Physiol. Part D. Genom. Proteom..

[42] Lambert C., Schmidt K., Karger M., Stadler M., Stradal T.E.B., Rottner K. (2025). Cytochalasans and their impact on actin filament remodeling. Biomolecules.

[43] Long X.W., Wu H., Ding Y.M., Qu C.L., Deng J. (2021). Biosynthetically inspired divergent syntheses of merocytochalasans. Chem.

