# Supplementary material for: Comparative Physiological and Metabolomic Responses of Procambarus clarkii and Macrobrachium nipponense to Combined Salinity–pH and NaHCO3 Alkalinity Stress
Source: Animals (Basel). 2026 Sep 3;16(17):2778. doi: 10.3390/ani16172778 (PMC13565080; doi:10.3390/ani16172778)
Supplement: Supplementary file 1 [file animals-16-02778-s001.zip › Statistical Analysis/Q_CvsQG/OPLS-DA permutation histogram.pdf]

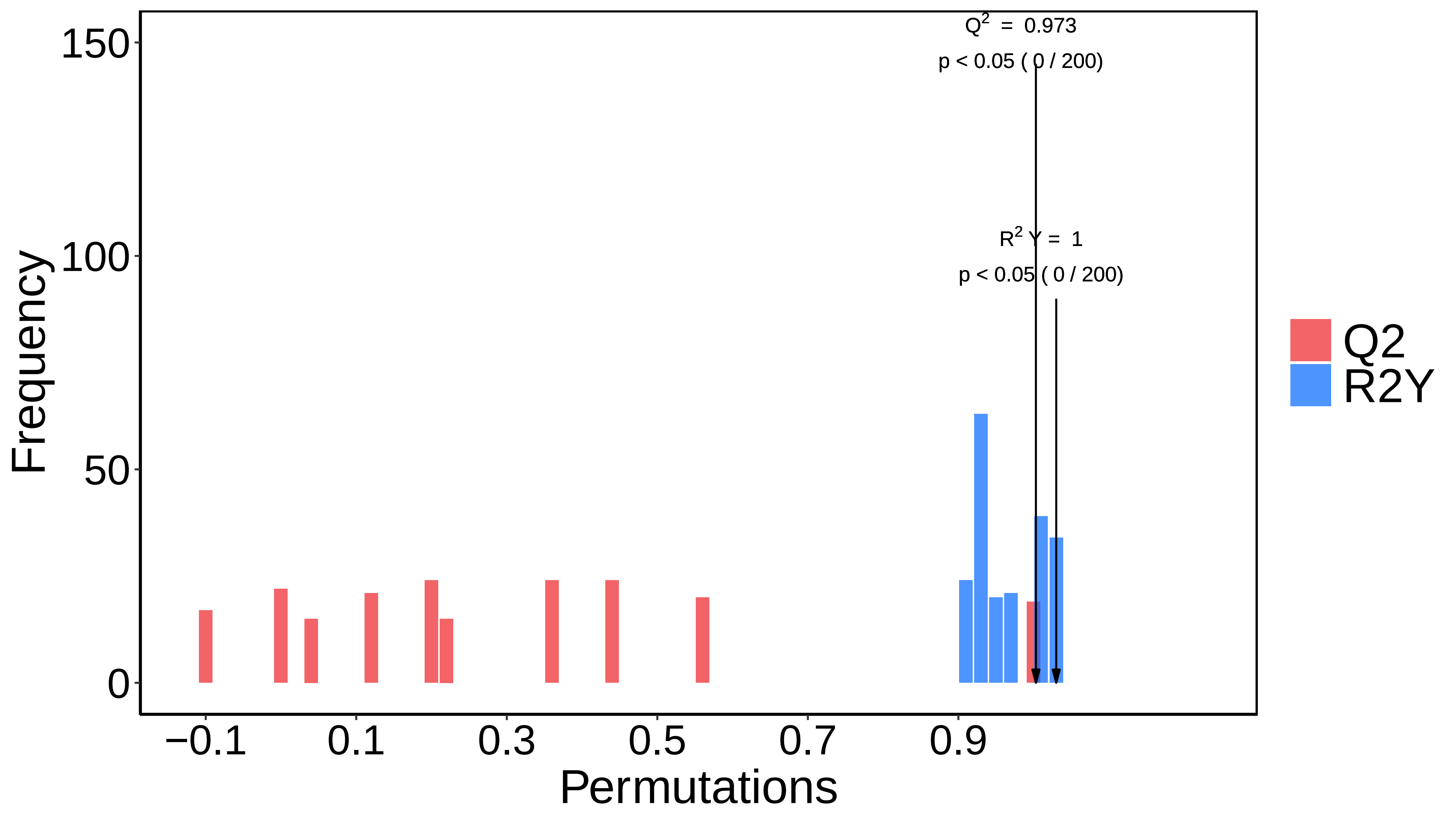

Q2 = 0.973
p < 0.05 (0 / 200)
R2Y = 1
p < 0.05 (0 / 200)
150
100
50
0
Frequency
−0.1
0.1
0.3
0.5
0.7
0.9
Permutations
Q2
R2Y
