# Supplementary material for: Comparative Physiological and Metabolomic Responses of Procambarus clarkii and Macrobrachium nipponense to Combined Salinity–pH and NaHCO3 Alkalinity Stress
Source: Animals (Basel). 2026 Sep 3;16(17):2778. doi: 10.3390/ani16172778 (PMC13565080; doi:10.3390/ani16172778)
Supplement: Supplementary file 1 [file animals-16-02778-s001.zip › Statistical Analysis/Q_CvsQG/PCA score plot 3D.pdf]

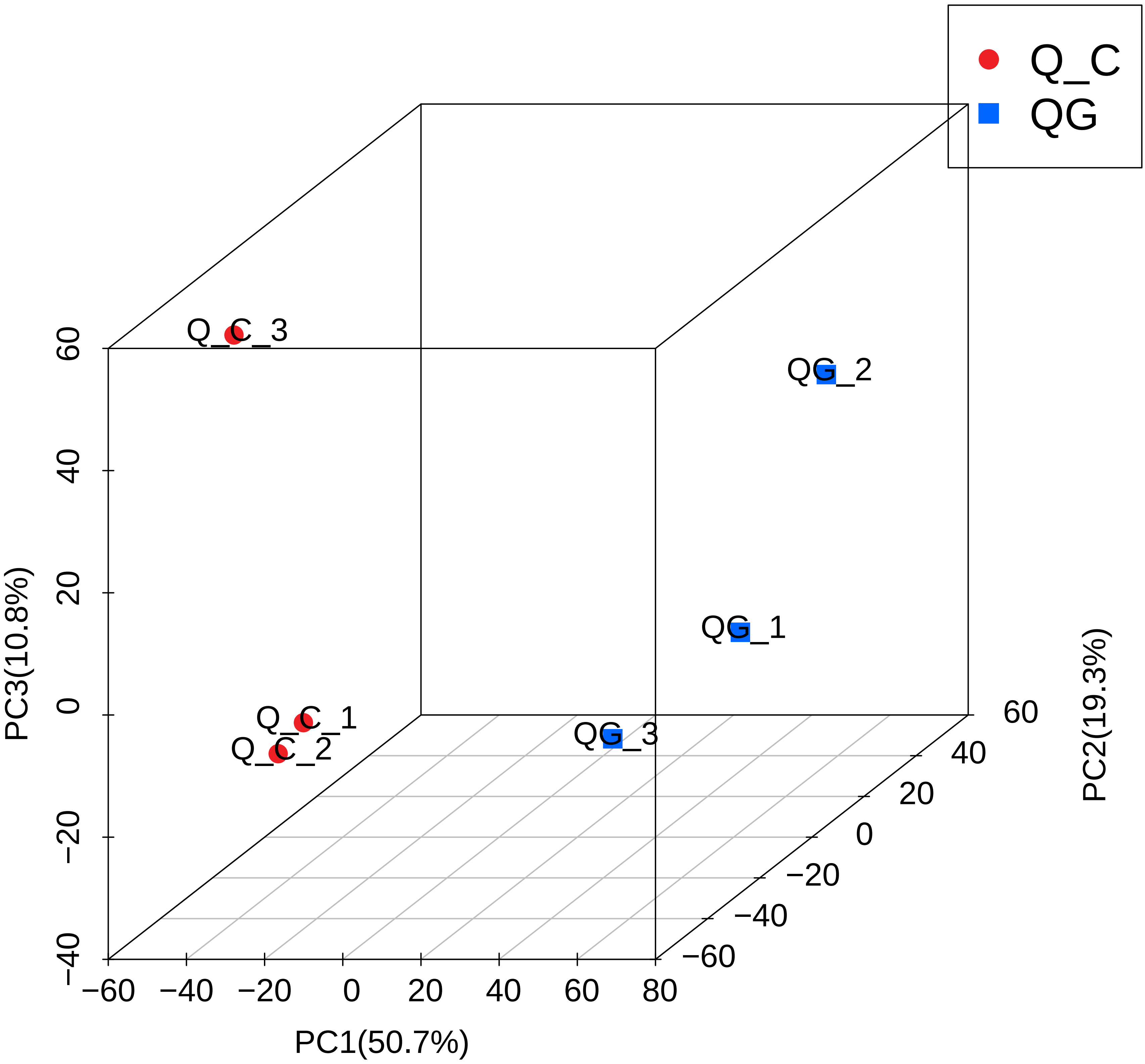

Q_C
QG
Q_C_3
QG_2
QG_1
Q_C_1
Q_C_2
QG_3
60
40
20
0
−20
−40
PC3(10.8%)
−60
−40
−20
0
20
40
60
80
PC1(50.7%)
−60
−40
−20
0
20
40
60
PC2(19.3%)
