# Supplementary material for: Comparative Physiological and Metabolomic Responses of Procambarus clarkii and Macrobrachium nipponense to Combined Salinity–pH and NaHCO3 Alkalinity Stress
Source: Animals (Basel). 2026 Sep 3;16(17):2778. doi: 10.3390/ani16172778 (PMC13565080; doi:10.3390/ani16172778)
Supplement: Supplementary file 1 [file animals-16-02778-s001.zip › Statistical Analysis/Q_CvsQG/PCA score plot.pdf]

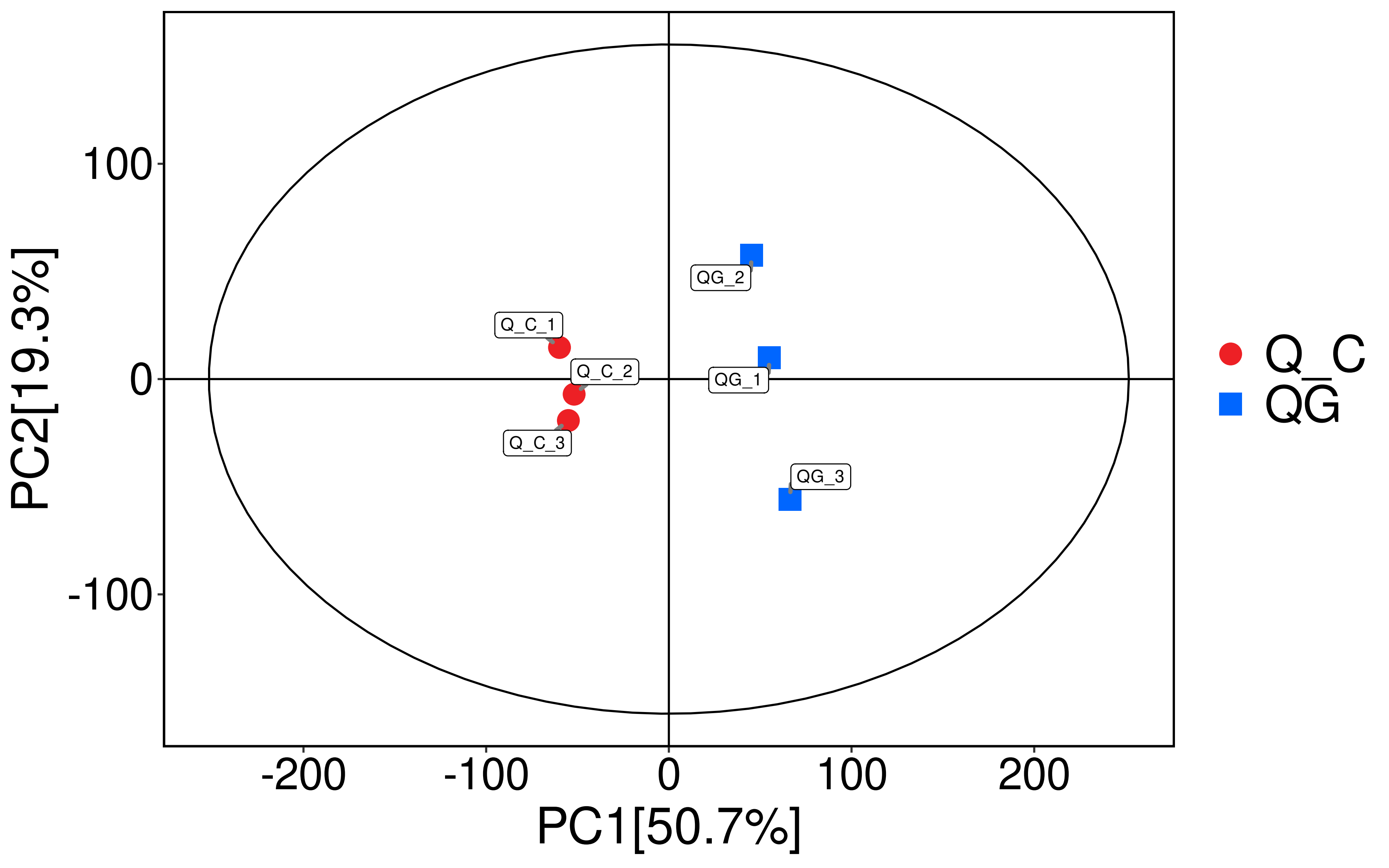

100
0
-100
PC2[19.3%]
-200
-100
0
100
200
PC1[50.7%]
Q_C_1
Q_C_2
Q_C_3
QG_1
QG_2
QG_3
Q_C
QG
