# Supplementary material for: Comparative Physiological and Metabolomic Responses of Procambarus clarkii and Macrobrachium nipponense to Combined Salinity–pH and NaHCO3 Alkalinity Stress
Source: Animals (Basel). 2026 Sep 3;16(17):2778. doi: 10.3390/ani16172778 (PMC13565080; doi:10.3390/ani16172778)
Supplement: Supplementary file 1 [file animals-16-02778-s001.zip › Statistical Analysis/Q_CvsQG/volcano plot with label.pdf]

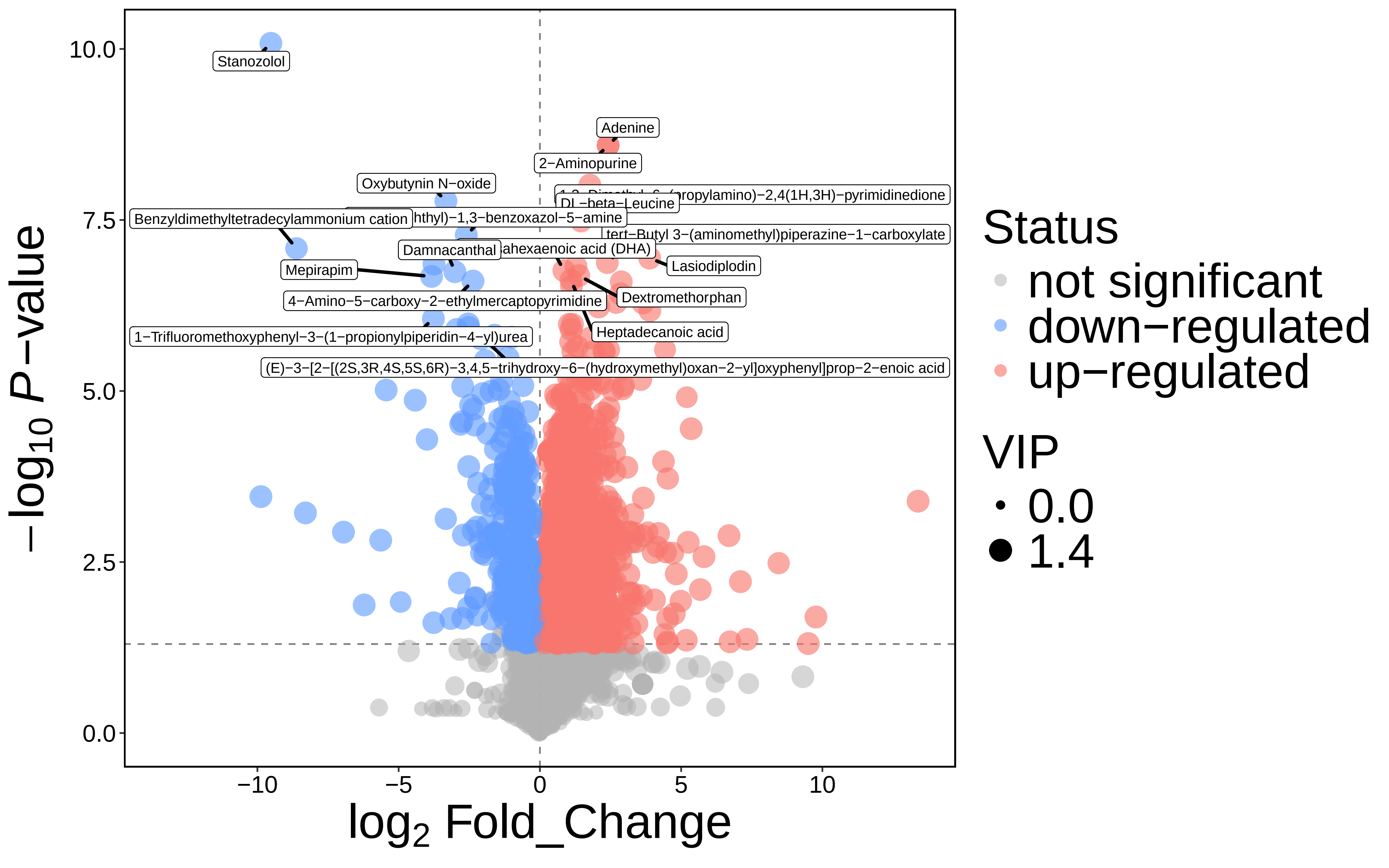
Stanozolol
Adenine
2−Aminopurine
Oxybutynin N−oxide
DL−beta−Leucine
Benzyldimethyltetradecylammonium cation
hthyl)−1,3−benzoxazol−5−amine
tert−Butyl 3−(aminomethyl)piperazine−1−carboxylate
Damnacanthal
ahexaenoic acid (DHA)
Lasiodiplodin
Mepirapim
Dextromethorphan
4−Amino−5−carboxy−2−ethylmercaptopyrimidine
Heptadecanoic acid
1−Trifluoromethoxyphenyl−3−(1−propionylpiperidin−4−yl)urea
(E)−3−[2−[(2S,3R,4S,5S,6R)−3,4,5−trihydroxy−6−(hydroxymethyl)oxan−2−yl]oxyphenyl]prop−2−enoic acid
−log10 P−value
log2 Fold_Change
10.0
7.5
5.0
2.5
0.0
−10
−5
0
5
10
Status
not significant
down−regulated
up−regulated
VIP
0.0
1.4
