# Supplementary material for: Comparative Physiological and Metabolomic Responses of Procambarus clarkii and Macrobrachium nipponense to Combined Salinity–pH and NaHCO3 Alkalinity Stress
Source: Animals (Basel). 2026 Sep 3;16(17):2778. doi: 10.3390/ani16172778 (PMC13565080; doi:10.3390/ani16172778)
Supplement: Supplementary file 1 [file animals-16-02778-s001.zip › Statistical Analysis/Q_CvsQG/volcano plot.pdf]

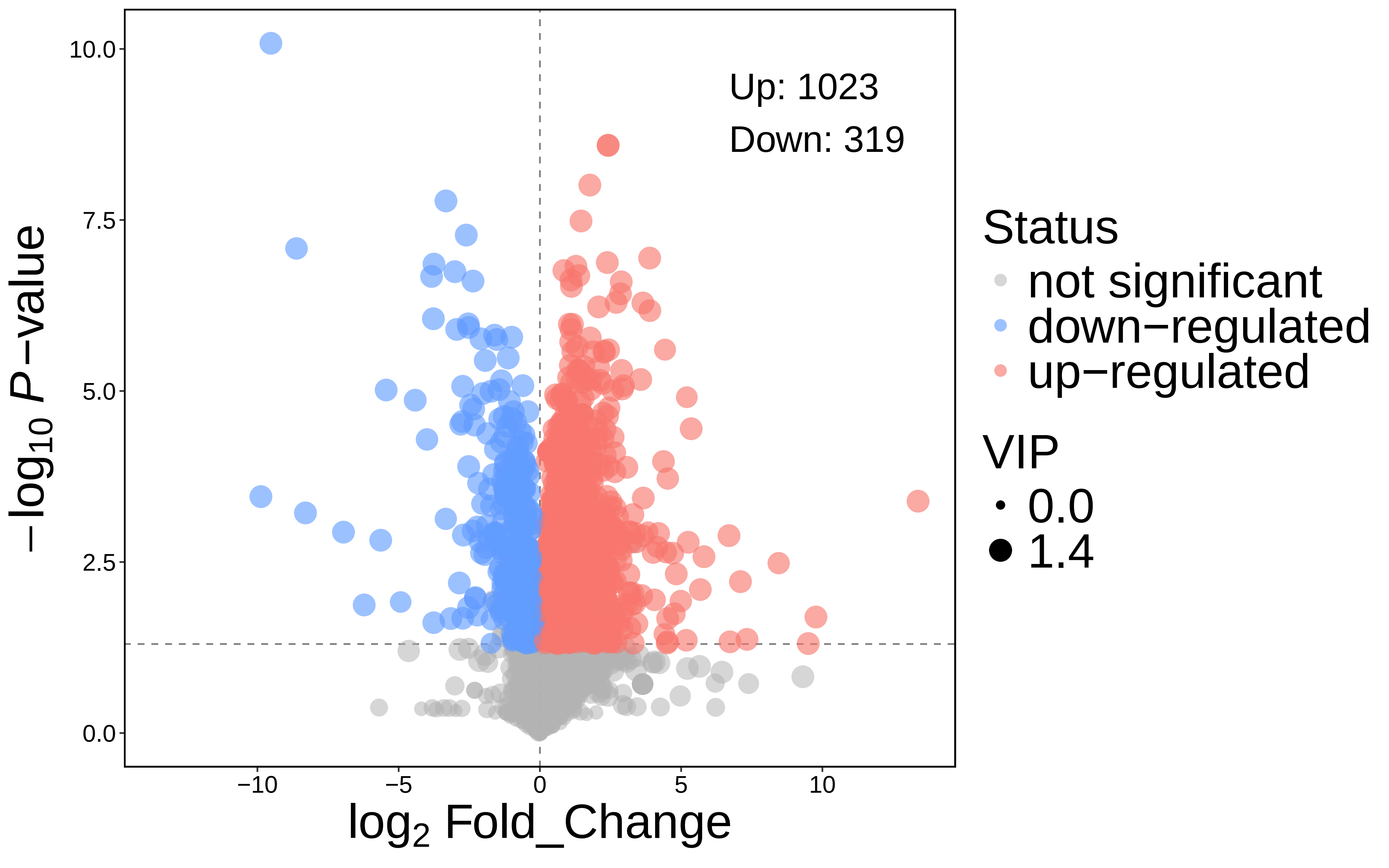
Up: 1023
Down: 319
−log10 P−value
log2 Fold_Change
10.0
7.5
5.0
2.5
0.0
−10
−5
0
5
10
Status
not significant
down−regulated
up−regulated
VIP
0.0
1.4
