# Supplementary material for: Comparative Physiological and Metabolomic Responses of Procambarus clarkii and Macrobrachium nipponense to Combined Salinity–pH and NaHCO3 Alkalinity Stress
Source: Animals (Basel). 2026 Sep 3;16(17):2778. doi: 10.3390/ani16172778 (PMC13565080; doi:10.3390/ani16172778)
Supplement: Supplementary file 1 [file animals-16-02778-s001.zip › Statistical Analysis/Q_CvsQG/Z-Score Plot.pdf]

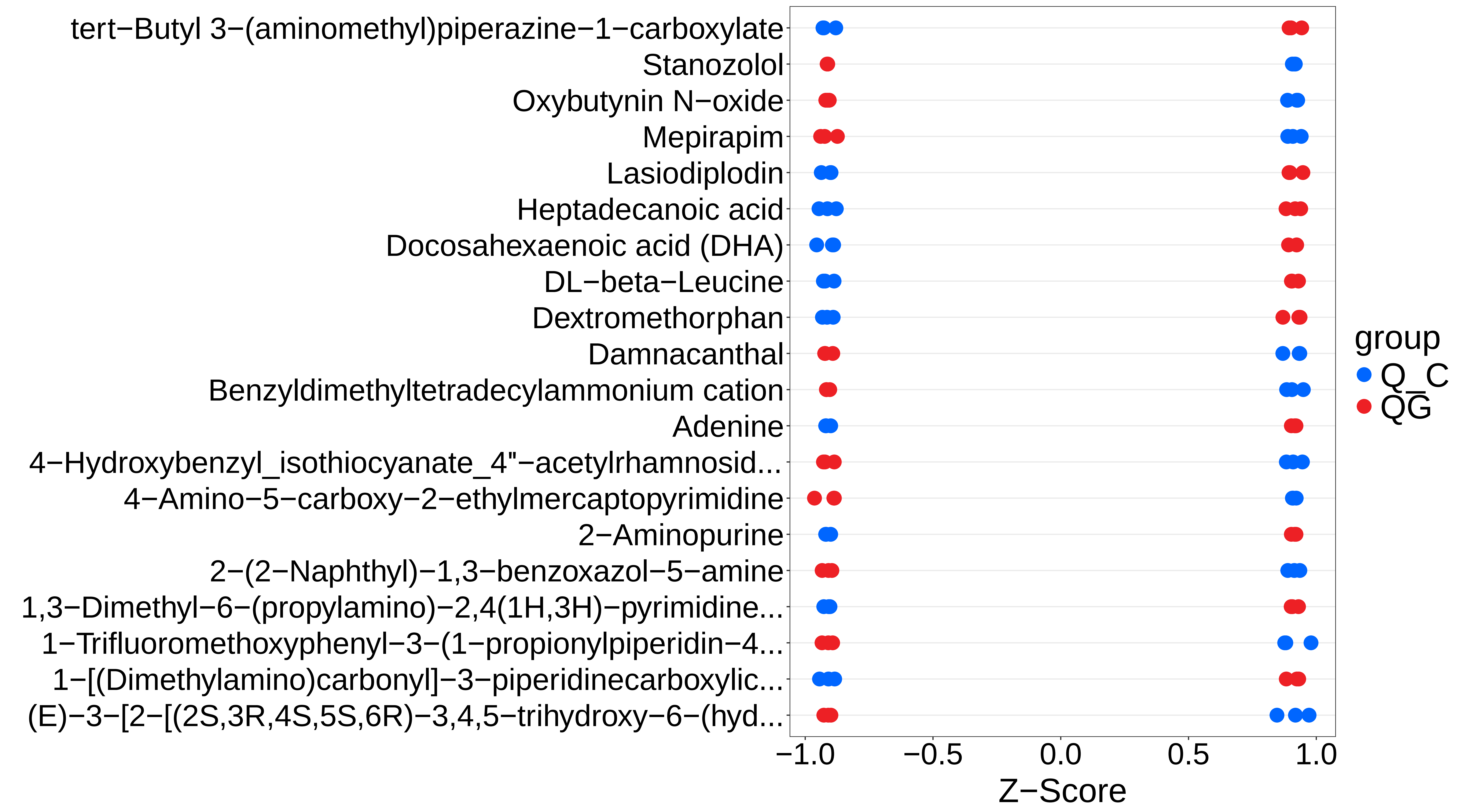

tert−Butyl 3−(aminomethyl)piperazine−1−carboxylate
Stanozolol
Oxybutynin N−oxide
Mepirapim
Lasiodiplodin
Heptadecanoic acid
Docosahexaenoic acid (DHA)
DL−beta−Leucine
Dextromethorphan
Damnacanthal
Benzyldimethyltetradecylammonium cation
Adenine
4−Hydroxybenzyl_isothiocyanate_4"−acetylrhamnosid...
4−Amino−5−carboxy−2−ethylmercaptopyrimidine
2−Aminopurine
2−(2−Naphthyl)−1,3−benzoxazol−5−amine
1,3−Dimethyl−6−(propylamino)−2,4(1H,3H)−pyrimidine...
1−Trifluoromethoxyphenyl−3−(1−propionylpiperidin−4...
1−[(Dimethylamino)carbonyl]−3−piperidinecarboxylic...
(E)−3−[2−[(2S,3R,4S,5S,6R)−3,4,5−trihydroxy−6−(hyd...
−1.0
−0.5
0.0
0.5
1.0
Z−Score
group
Q_C
QG
