# Supplementary material for: Comparative Physiological and Metabolomic Responses of Procambarus clarkii and Macrobrachium nipponense to Combined Salinity–pH and NaHCO3 Alkalinity Stress
Source: Animals (Basel). 2026 Sep 3;16(17):2778. doi: 10.3390/ani16172778 (PMC13565080; doi:10.3390/ani16172778)
Supplement: Supplementary file 1 [file animals-16-02778-s001.zip › Statistical Analysis/Q_CvsQJ/PCA score plot 3D.pdf]

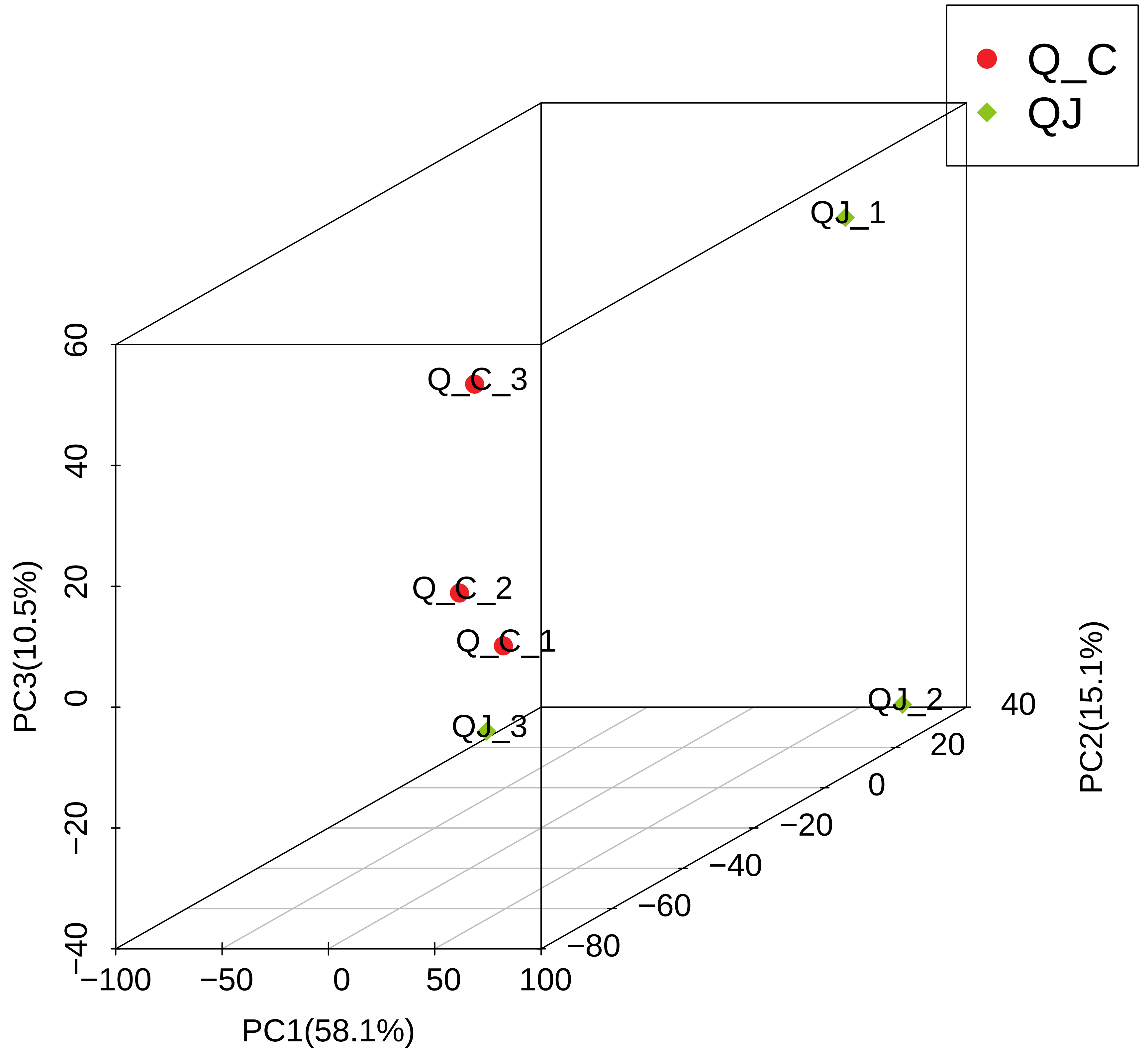
Q_C
QJ
QJ_1
Q_C_3
Q_C_2
Q_C_1
QJ_2
QJ_3
60
40
20
0
−20
−40
PC3(10.5%)
−100
−50
0
50
100
PC1(58.1%)
40
20
0
−20
−40
−60
−80
PC2(15.1%)
