# Supplementary material for: Comparative Physiological and Metabolomic Responses of Procambarus clarkii and Macrobrachium nipponense to Combined Salinity–pH and NaHCO3 Alkalinity Stress
Source: Animals (Basel). 2026 Sep 3;16(17):2778. doi: 10.3390/ani16172778 (PMC13565080; doi:10.3390/ani16172778)
Supplement: Supplementary file 1 [file animals-16-02778-s001.zip › Statistical Analysis/Q_CvsQJ/volcano plot with label.pdf]

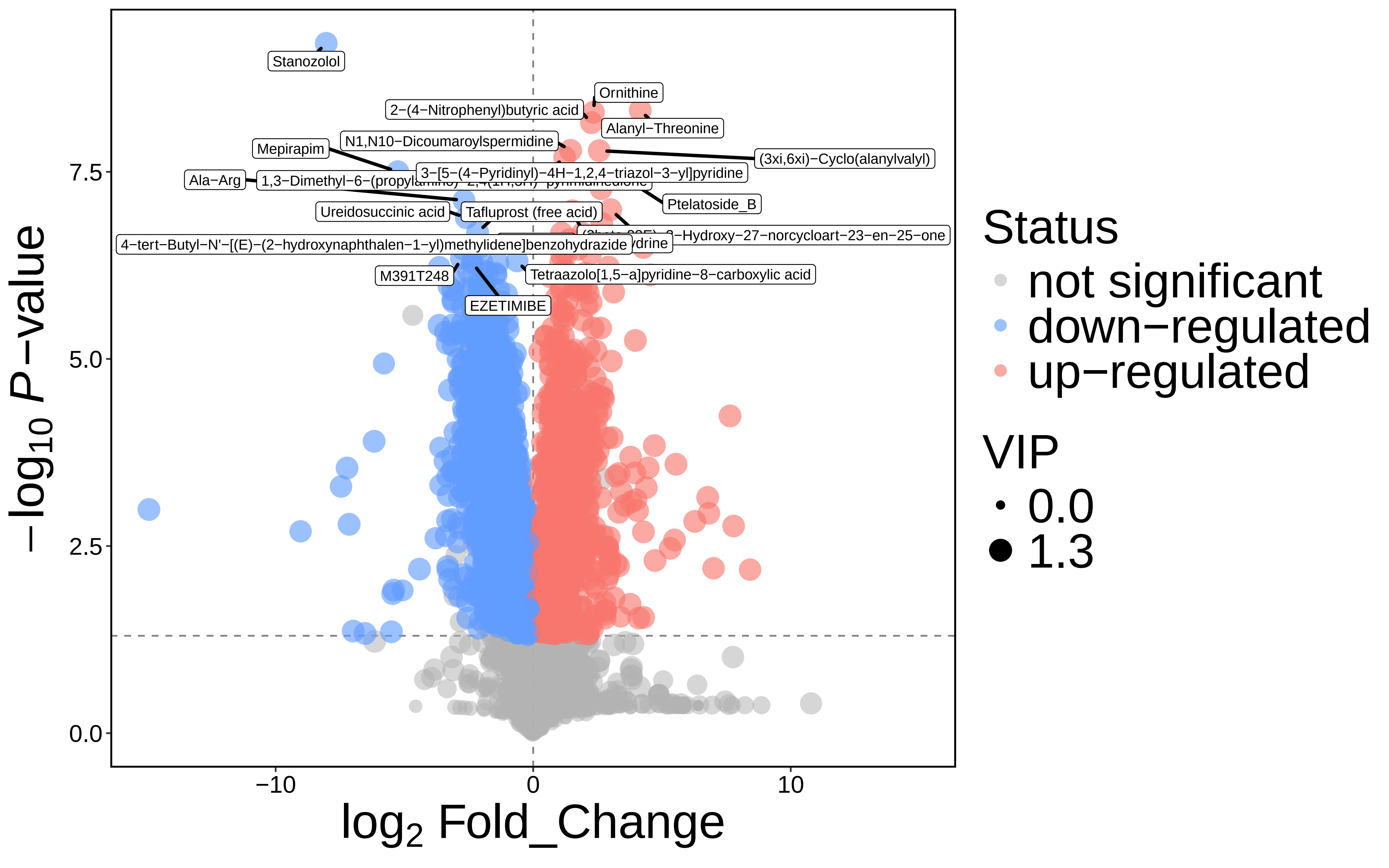
Stanozolol
Ornithine
2−(4−Nitrophenyl)butyric acid
Alanyl−Threonine
N1,N10−Dicoumaroylspermidine
Mepirapim
(3xi,6xi)−Cyclo(alanylvalyl)
3−[5−(4−Pyridinyl)−4H−1,2,4−triazol−3−yl]pyridine
Ala−Arg
1,3−Dimethyl−6−(propyl
Ptelatoside_B
Ureidosuccinic acid
Tafluprost (free acid)
−Hydroxy−27−norcycloart−23−en−25−one
4−tert−Butyl−N'−[(E)−(2−hydroxynaphthalen−1−yl)methylidene]benzohydrazide
M391T248
Tetraazolo[1,5−a]pyridine−8−carboxylic acid
EZETIMIBE
7.5
5.0
2.5
0.0
−10
0
10
log2 Fold_Change
−log10 P−value
Status
not significant
down−regulated
up−regulated
VIP
0.0
1.3
