# Supplementary material for: Comparative Physiological and Metabolomic Responses of Procambarus clarkii and Macrobrachium nipponense to Combined Salinity–pH and NaHCO3 Alkalinity Stress
Source: Animals (Basel). 2026 Sep 3;16(17):2778. doi: 10.3390/ani16172778 (PMC13565080; doi:10.3390/ani16172778)
Supplement: Supplementary file 1 [file animals-16-02778-s001.zip › Statistical Analysis/Q_CvsQJ/Z-Score Plot.pdf]

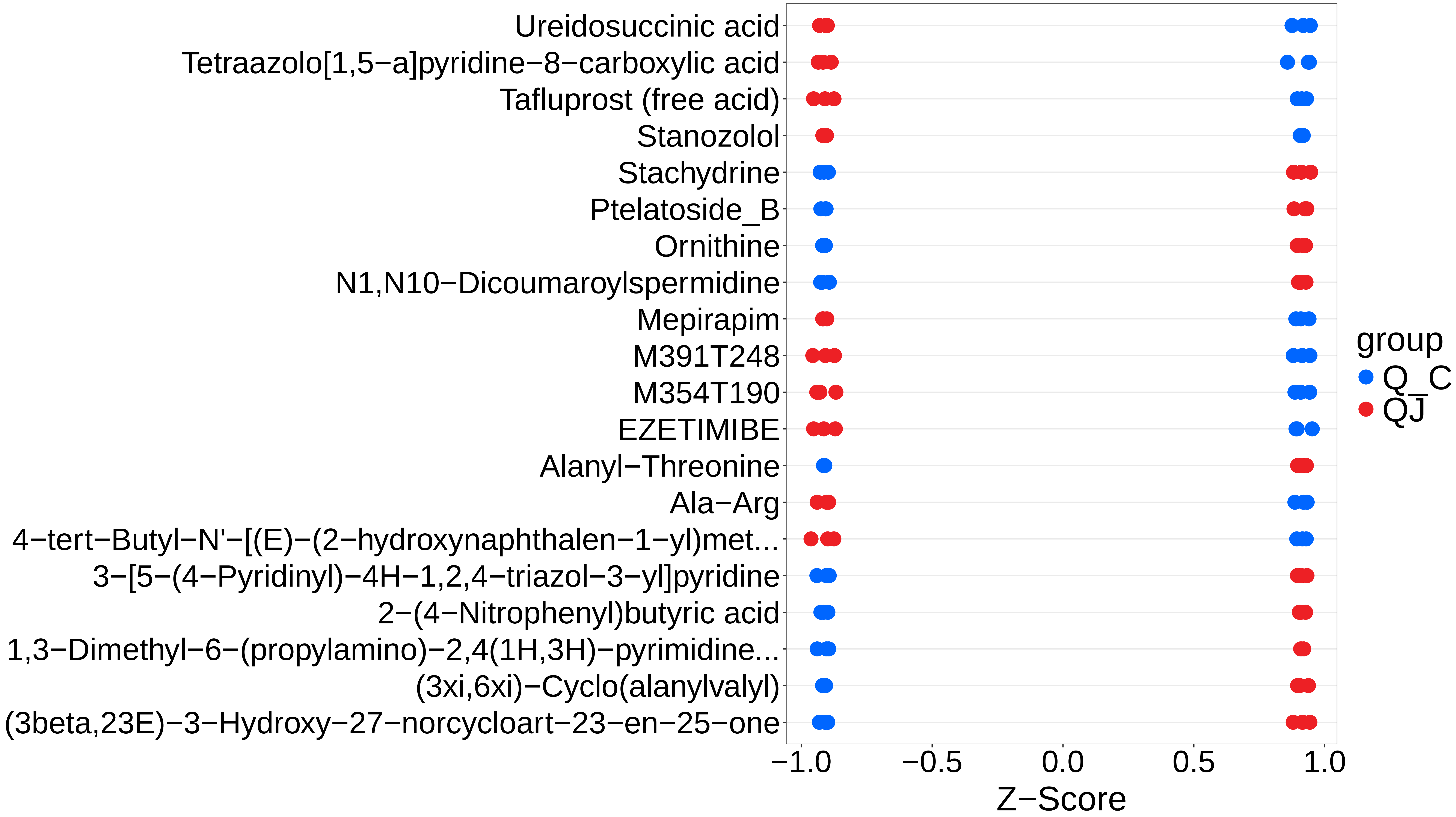

Ureidosuccinic acid
Tetraazolo[1,5−a]pyridine−8−carboxylic acid
Tafluprost (free acid)
Stanozolol
Stachydrine
Ptelatoside_B
Ornithine
N1,N10−Dicoumaroylspermidine
Mepirapim
M391T248
M354T190
EZETIMIBE
Alanyl−Threonine
Ala−Arg
4−tert−Butyl−N'−[(E)−(2−hydroxynaphthalen−1−yl)met...
3−[5−(4−Pyridinyl)−4H−1,2,4−triazol−3−yl]pyridine
2−(4−Nitrophenyl)butyric acid
1,3−Dimethyl−6−(propylamino)−2,4(1H,3H)−pyrimidine...
(3xi,6xi)−Cyclo(alanylvalyl)
(3beta,23E)−3−Hydroxy−27−norcycloart−23−en−25−one
−1.0
−0.5
0.0
0.5
1.0
Z−Score
group
Q_C
QJ
