# Supplementary material for: Comparative Physiological and Metabolomic Responses of Procambarus clarkii and Macrobrachium nipponense to Combined Salinity–pH and NaHCO3 Alkalinity Stress
Source: Animals (Basel). 2026 Sep 3;16(17):2778. doi: 10.3390/ani16172778 (PMC13565080; doi:10.3390/ani16172778)
Supplement: Supplementary file 1 [file animals-16-02778-s001.zip › Statistical Analysis/TOTAL/PCA score plot 3D.pdf]

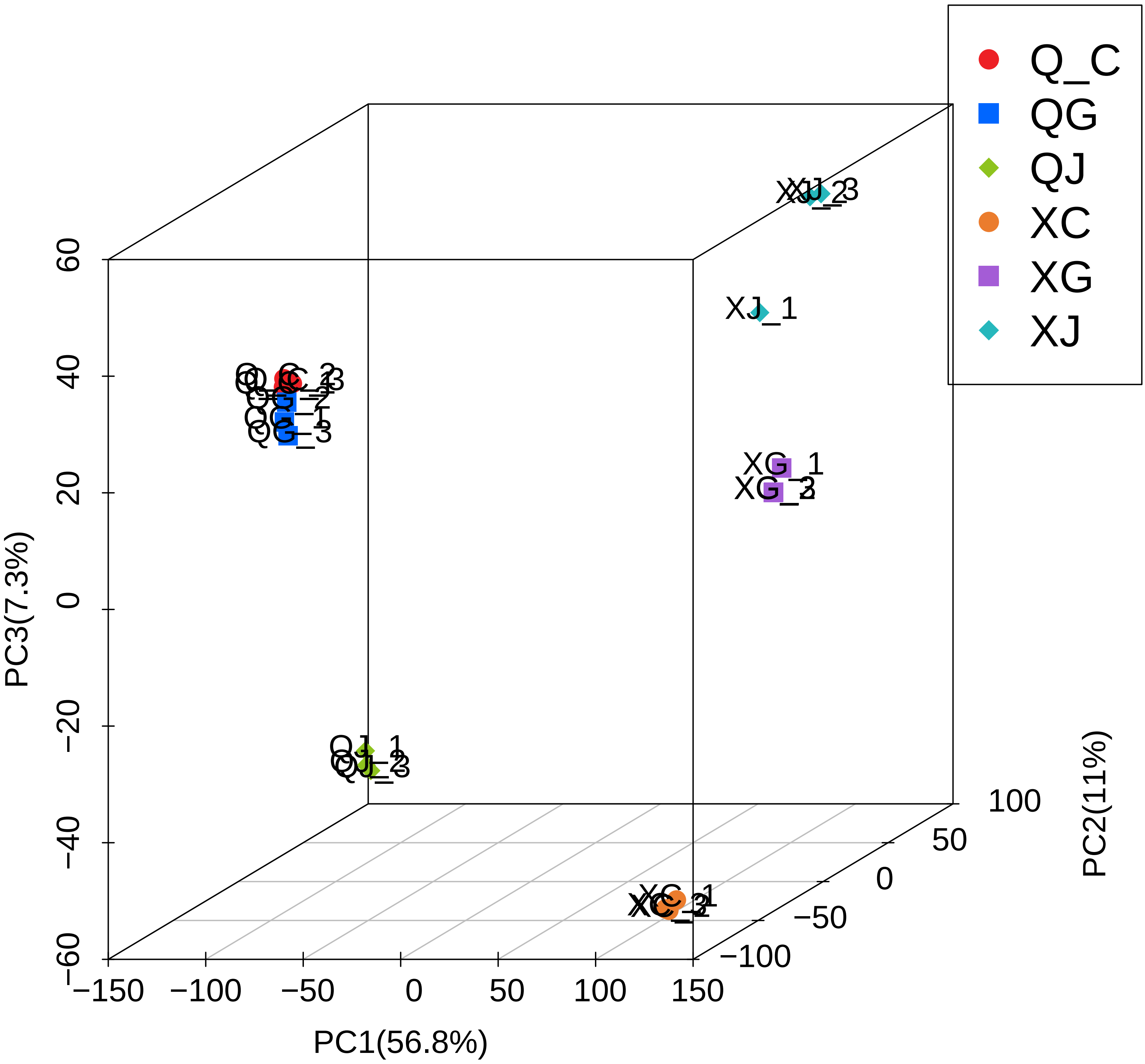

Q_C
QG
QJ
XC
XG
XJ
XJ_1
XG_1
QG_3
QJ_1
XC_1
PC1(56.8%)
PC2(11%)
PC3(7.3%)
−150
−100
−50
0
50
100
150
60
40
20
0
−20
−40
−60
