# Supplementary material for: Comparative Physiological and Metabolomic Responses of Procambarus clarkii and Macrobrachium nipponense to Combined Salinity–pH and NaHCO3 Alkalinity Stress
Source: Animals (Basel). 2026 Sep 3;16(17):2778. doi: 10.3390/ani16172778 (PMC13565080; doi:10.3390/ani16172778)
Supplement: Supplementary file 1 [file animals-16-02778-s001.zip › Statistical Analysis/TOTAL with QC/PCA score plot 3D.pdf]

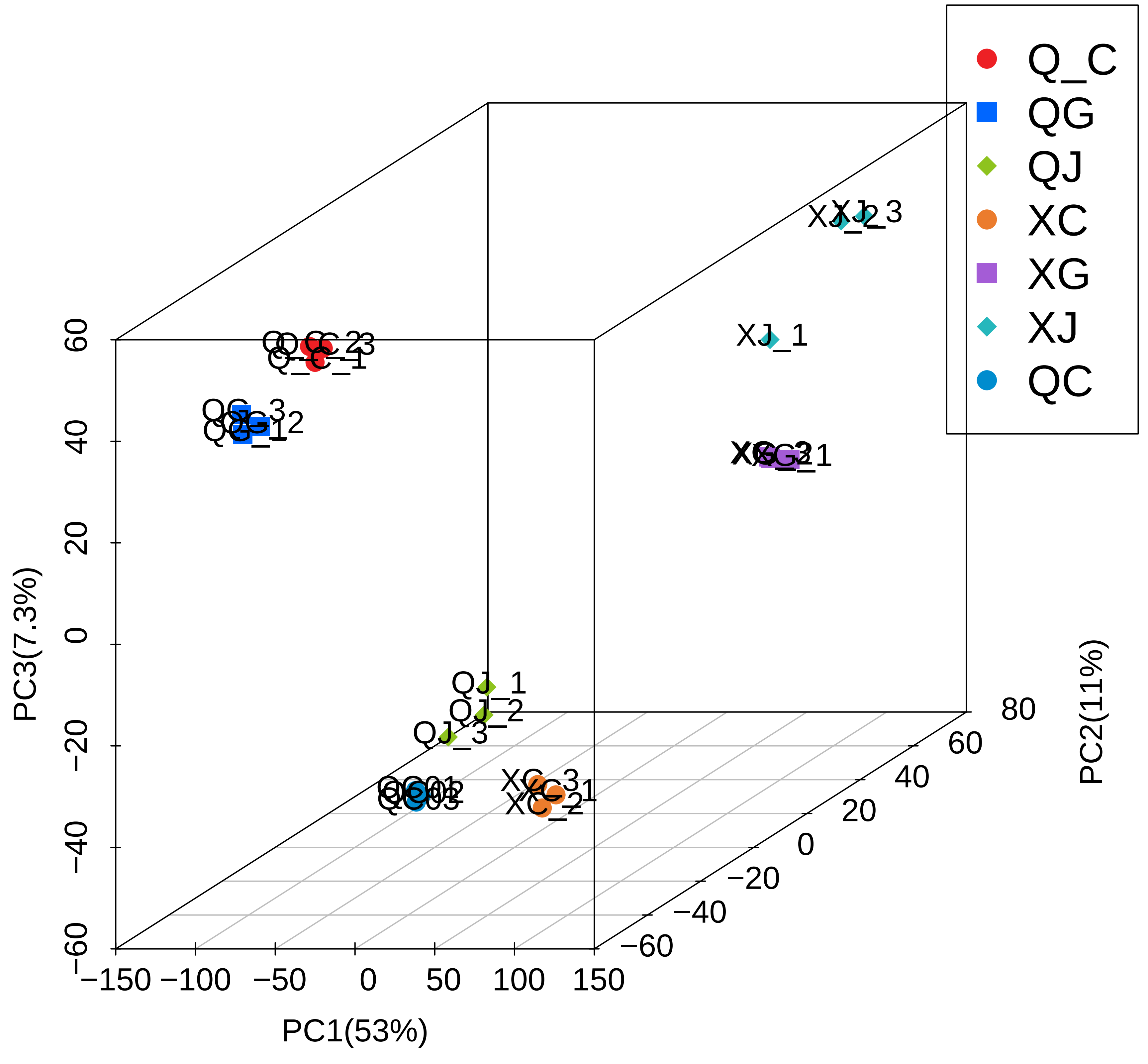
Q_C
QG
QJ
XC
XG
XJ
QC
PC1(53%)
PC2(11%)
PC3(7.3%)
−150
−100
−50
0
50
100
150
−60
−40
−20
0
20
40
60
80
XJ_1
QJ_1
QJ_2
QJ_3
XC_2
