# Supplementary material for: Comparative Physiological and Metabolomic Responses of Procambarus clarkii and Macrobrachium nipponense to Combined Salinity–pH and NaHCO3 Alkalinity Stress
Source: Animals (Basel). 2026 Sep 3;16(17):2778. doi: 10.3390/ani16172778 (PMC13565080; doi:10.3390/ani16172778)
Supplement: Supplementary file 1 [file animals-16-02778-s001.zip › Statistical Analysis/TOTAL with QC/PCA score plot.pdf]

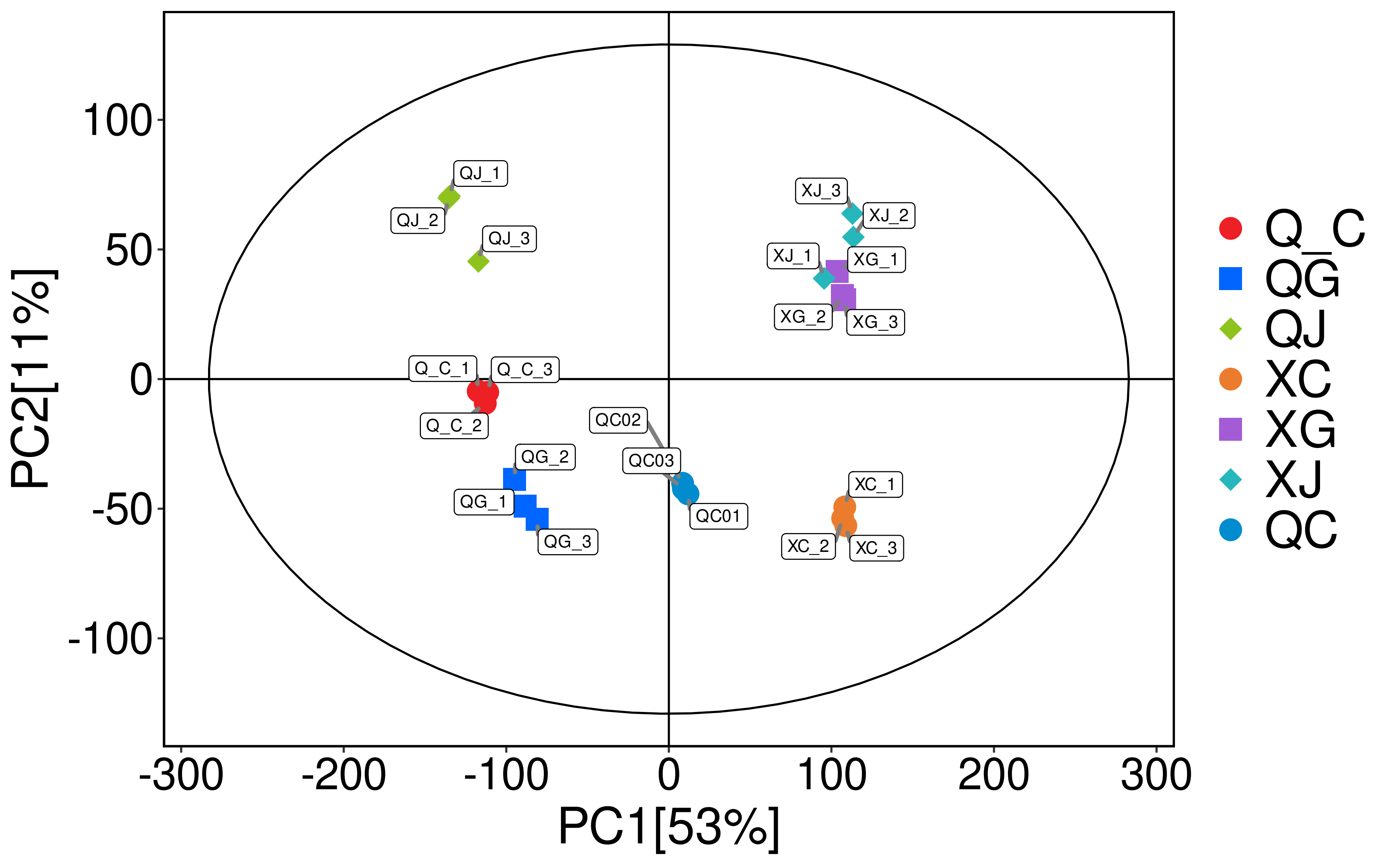

QJ_1
QJ_2
QJ_3
XJ_3
XJ_2
XJ_1
XG_1
XG_2
XG_3
Q_C_1
Q_C_3
Q_C_2
QC02
QC03
QC01
QG_2
QG_1
QG_3
XC_1
XC_2
XC_3
100
50
0
-50
-100
-300
-200
-100
0
100
200
300
PC1[53%]
PC2[11%]
Q_C
QG
QJ
XC
XG
XJ
QC
