# Supplementary material for: Comparative Physiological and Metabolomic Responses of Procambarus clarkii and Macrobrachium nipponense to Combined Salinity–pH and NaHCO3 Alkalinity Stress
Source: Animals (Basel). 2026 Sep 3;16(17):2778. doi: 10.3390/ani16172778 (PMC13565080; doi:10.3390/ani16172778)
Supplement: Supplementary file 1 [file animals-16-02778-s001.zip › Statistical Analysis/XCvsXG/OPLS-DA permutation plot.pdf]

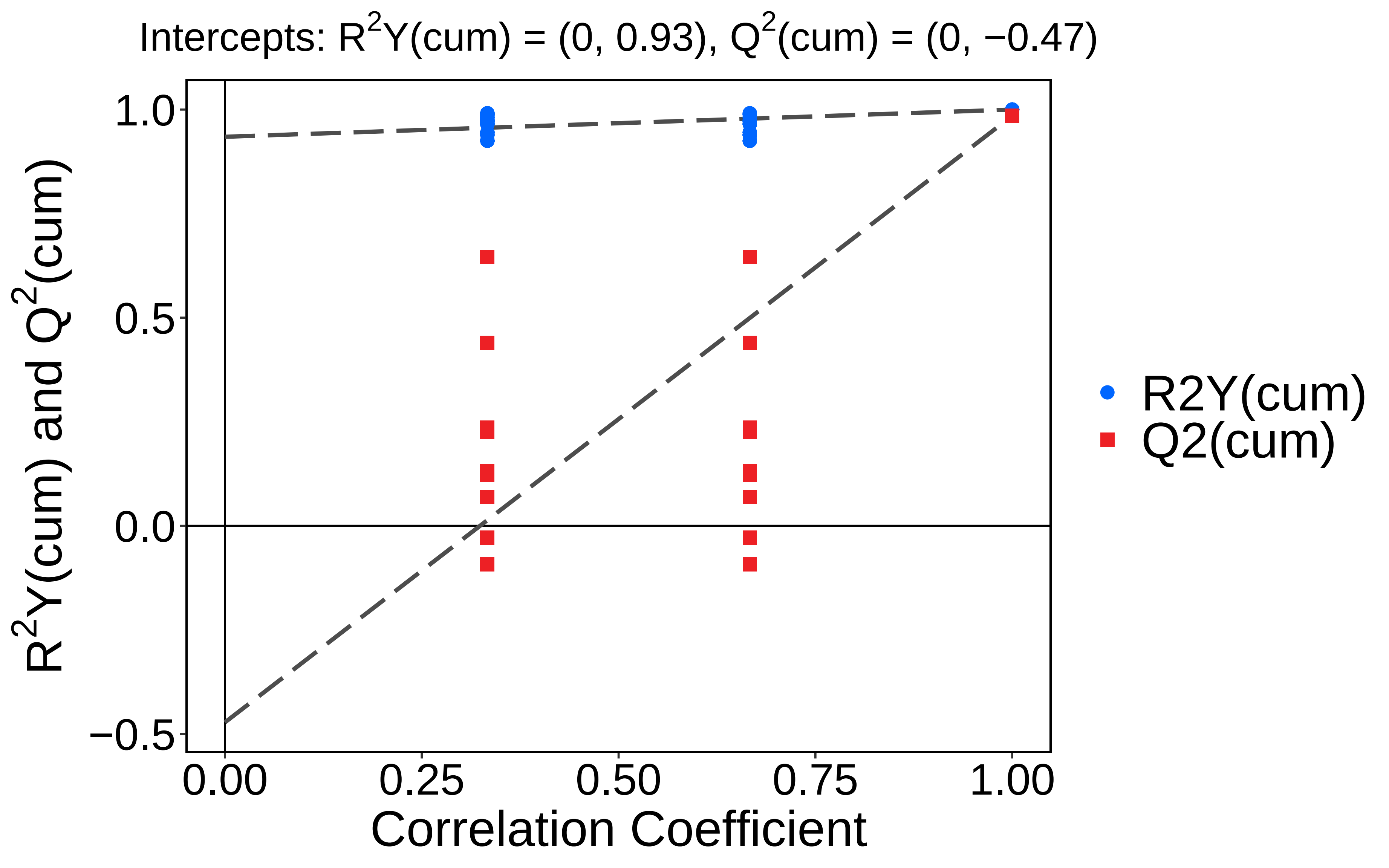
Intercepts: R2Y(cum) = (0, 0.93), Q2(cum) = (0, −0.47)
1.0
0.5
0.0
−0.5
0.00
0.25
0.50
0.75
1.00
Correlation Coefficient
R2Y(cum) and Q2(cum)
R2Y(cum)
Q2(cum)
