# Supplementary material for: Comparative Physiological and Metabolomic Responses of Procambarus clarkii and Macrobrachium nipponense to Combined Salinity–pH and NaHCO3 Alkalinity Stress
Source: Animals (Basel). 2026 Sep 3;16(17):2778. doi: 10.3390/ani16172778 (PMC13565080; doi:10.3390/ani16172778)
Supplement: Supplementary file 1 [file animals-16-02778-s001.zip › Statistical Analysis/XCvsXG/PCA score plot 3D.pdf]

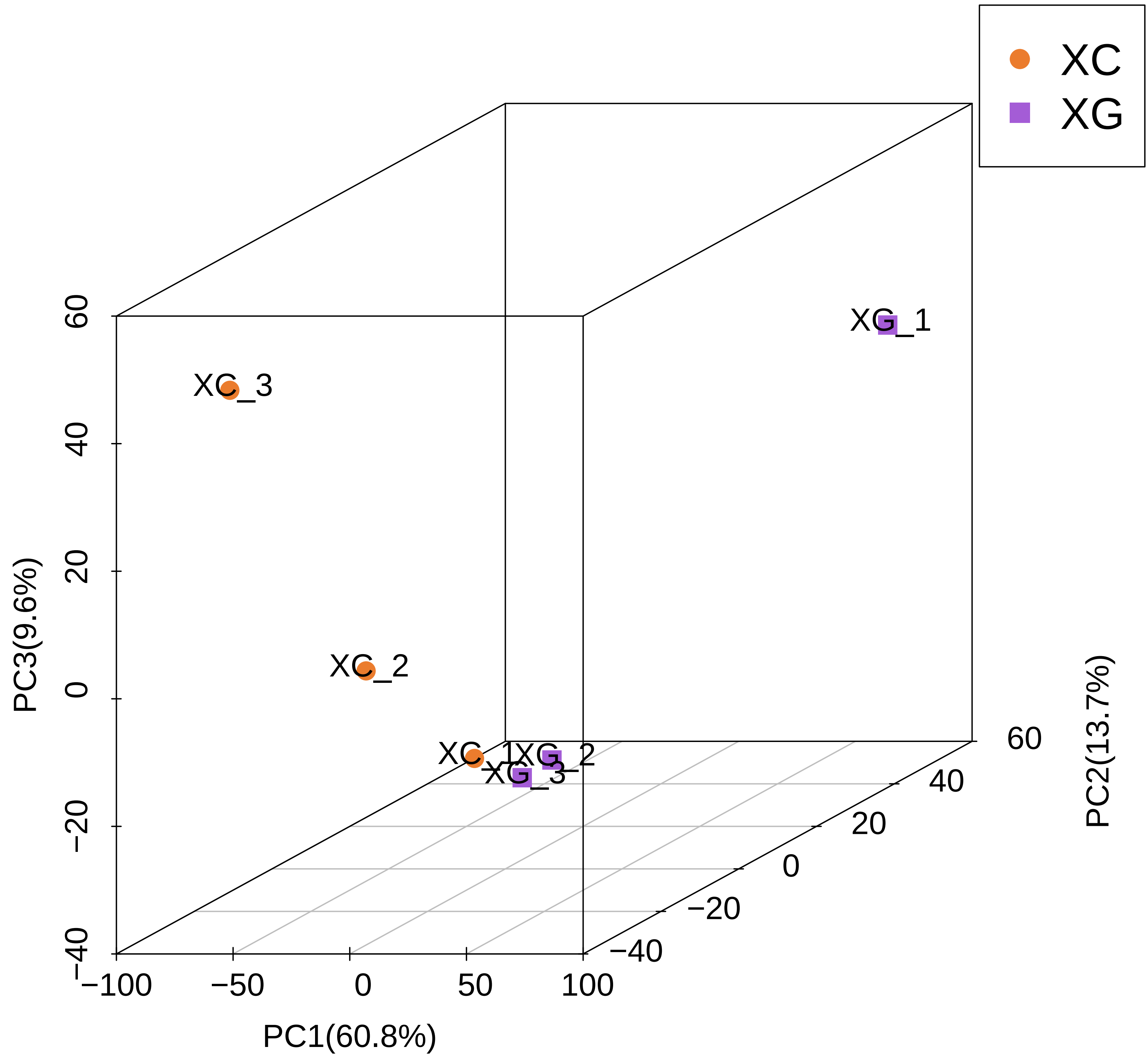
XC
XG
XG_1
XC_3
XC_2
XC_1
XG_2
XG_3
60
40
20
0
−20
−40
PC3(9.6%)
−100
−50
0
50
100
PC1(60.8%)
−40
−20
0
20
40
60
PC2(13.7%)
