# Supplementary material for: Comparative Physiological and Metabolomic Responses of Procambarus clarkii and Macrobrachium nipponense to Combined Salinity–pH and NaHCO3 Alkalinity Stress
Source: Animals (Basel). 2026 Sep 3;16(17):2778. doi: 10.3390/ani16172778 (PMC13565080; doi:10.3390/ani16172778)
Supplement: Supplementary file 1 [file animals-16-02778-s001.zip › Statistical Analysis/XCvsXG/PCA score plot.pdf]

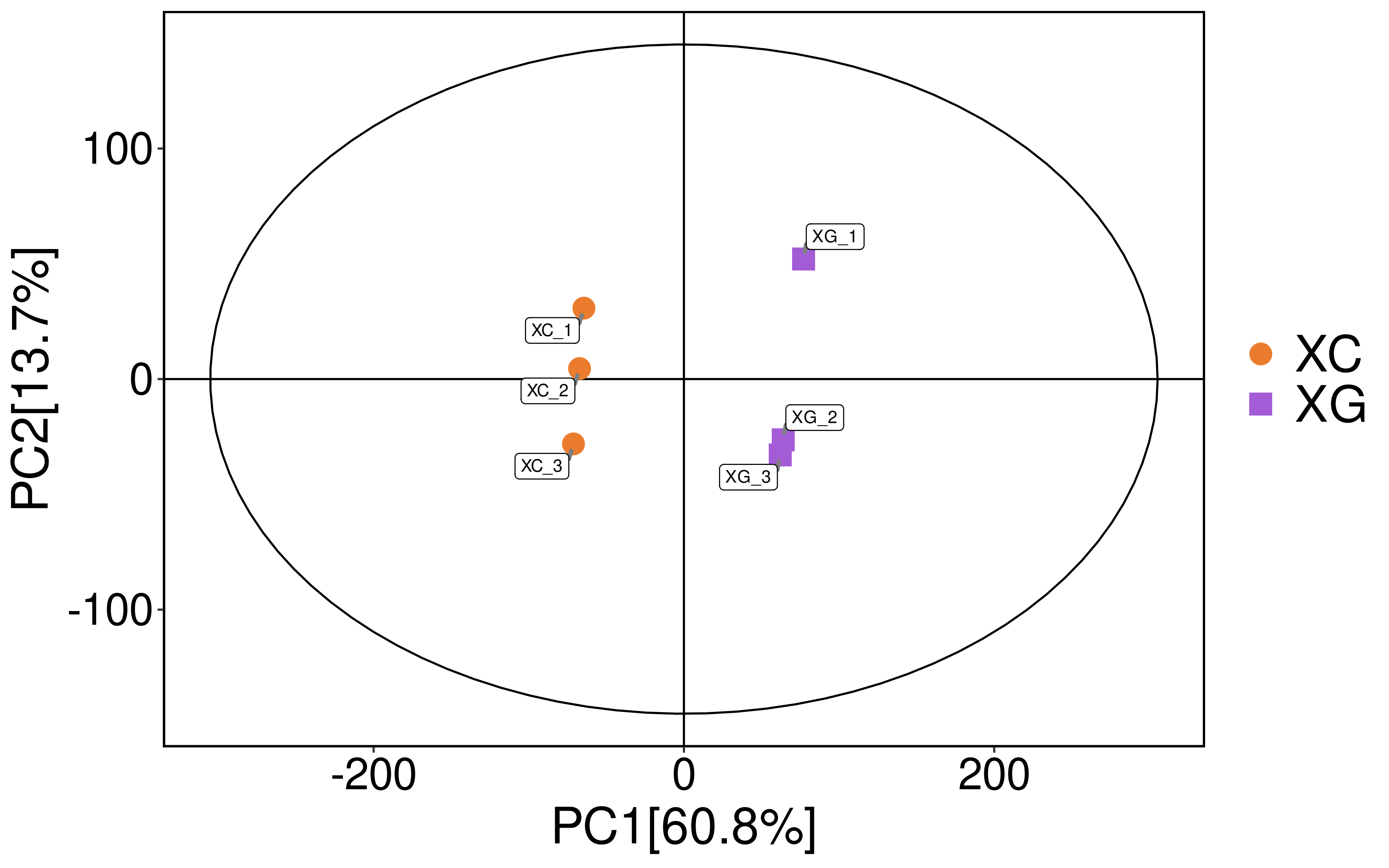
100
0
-100
-200
0
200
PC1[60.8%]
PC2[13.7%]
XC_1
XC_2
XC_3
XG_1
XG_2
XG_3
XC
XG
