# Supplementary material for: Comparative Physiological and Metabolomic Responses of Procambarus clarkii and Macrobrachium nipponense to Combined Salinity–pH and NaHCO3 Alkalinity Stress
Source: Animals (Basel). 2026 Sep 3;16(17):2778. doi: 10.3390/ani16172778 (PMC13565080; doi:10.3390/ani16172778)
Supplement: Supplementary file 1 [file animals-16-02778-s001.zip › Statistical Analysis/XCvsXG/volcano plot with label.pdf]

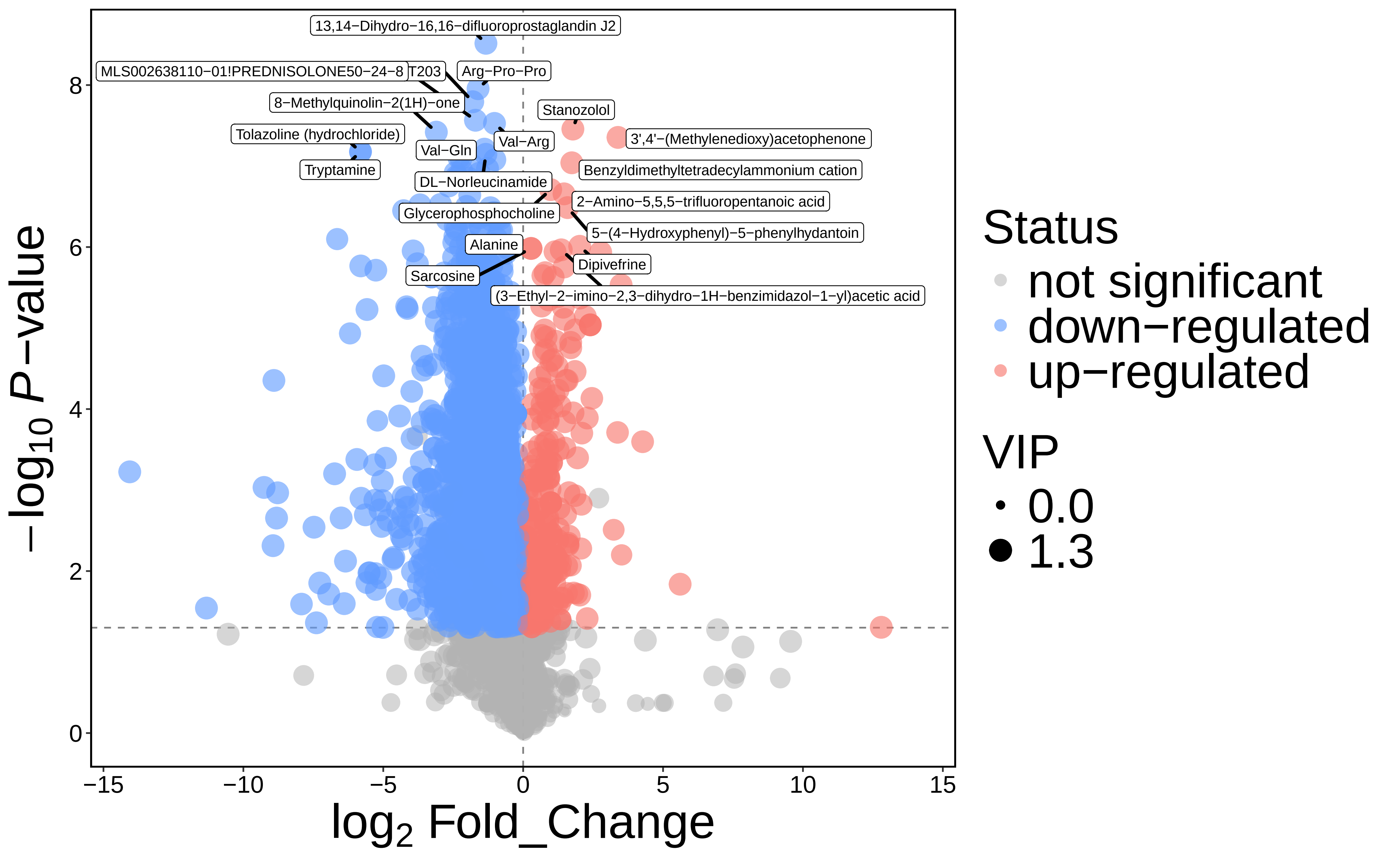

13,14−Dihydro−16,16−difluoroprostaglandin J2
MLS002638110−01!PREDNISOLONE50−24−8
T203
Arg−Pro−Pro
8−Methylquinolin−2(1H)−one
Stanozolol
Tolazoline (hydrochloride)
Val−Arg
3',4'−(Methylenedioxy)acetophenone
Val−Gln
Tryptamine
Benzyldimethyltetradecylammonium cation
DL−Norleucinamide
2−Amino−5,5,5−trifluoropentanoic acid
Glycerophosphocholine
5−(4−Hydroxyphenyl)−5−phenylhydantoin
Alanine
Dipivefrine
Sarcosine
(3−Ethyl−2−imino−2,3−dihydro−1H−benzimidazol−1−yl)acetic acid
−log10 P−value
log2 Fold_Change
Status
not significant
down−regulated
up−regulated
VIP
0.0
1.3
