# Supplementary material for: Comparative Physiological and Metabolomic Responses of Procambarus clarkii and Macrobrachium nipponense to Combined Salinity–pH and NaHCO3 Alkalinity Stress
Source: Animals (Basel). 2026 Sep 3;16(17):2778. doi: 10.3390/ani16172778 (PMC13565080; doi:10.3390/ani16172778)
Supplement: Supplementary file 1 [file animals-16-02778-s001.zip › Statistical Analysis/XCvsXG/volcano plot.pdf]

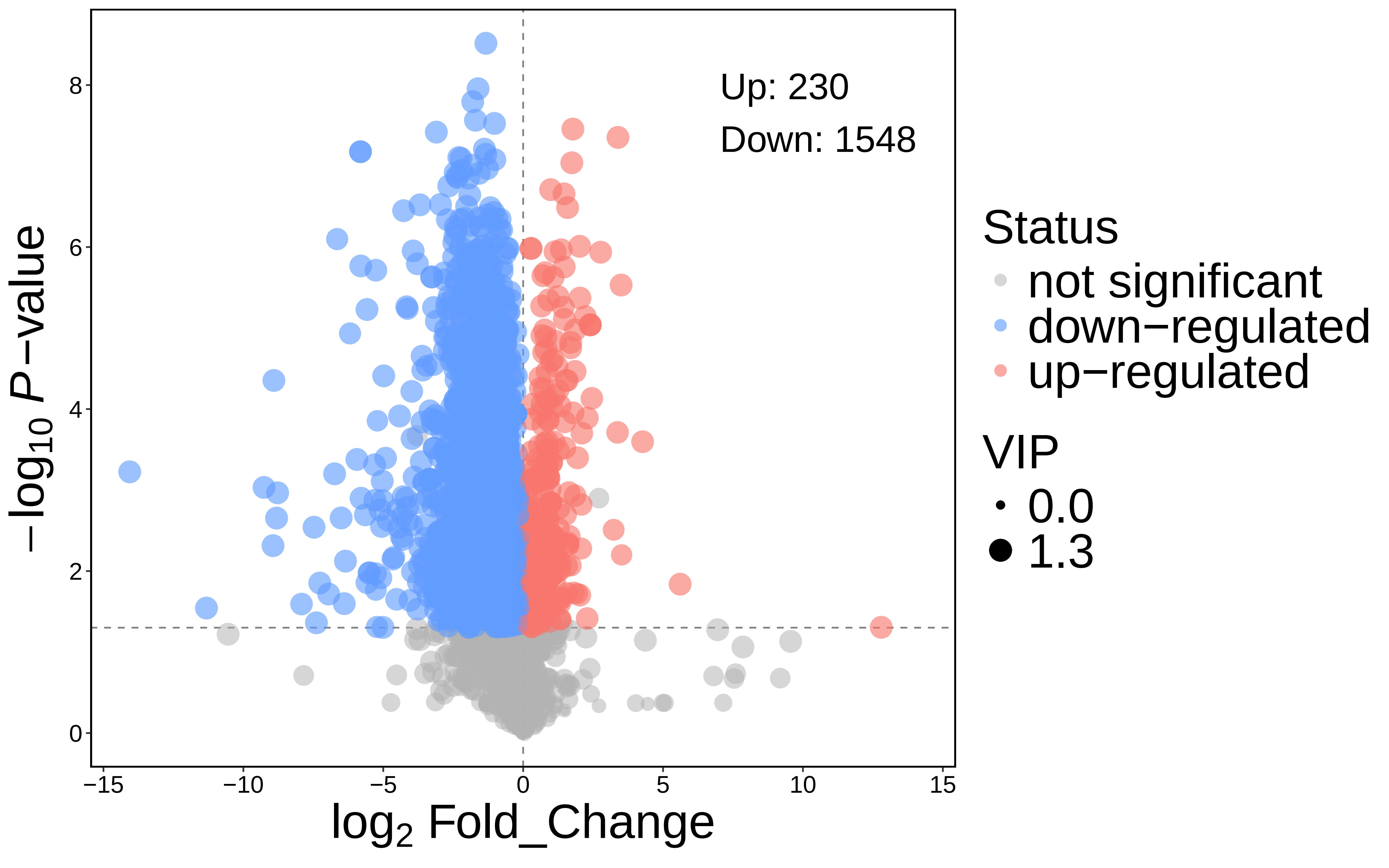
Up: 230
Down: 1548
Status
not significant
down−regulated
up−regulated
VIP
0.0
1.3
−log10 P−value
log2 Fold_Change
8
6
4
2
0
−15
−10
−5
0
5
10
15
