# Supplementary material for: Comparative Physiological and Metabolomic Responses of Procambarus clarkii and Macrobrachium nipponense to Combined Salinity–pH and NaHCO3 Alkalinity Stress
Source: Animals (Basel). 2026 Sep 3;16(17):2778. doi: 10.3390/ani16172778 (PMC13565080; doi:10.3390/ani16172778)
Supplement: Supplementary file 1 [file animals-16-02778-s001.zip › Statistical Analysis/XCvsXG/Z-Score Plot.pdf]

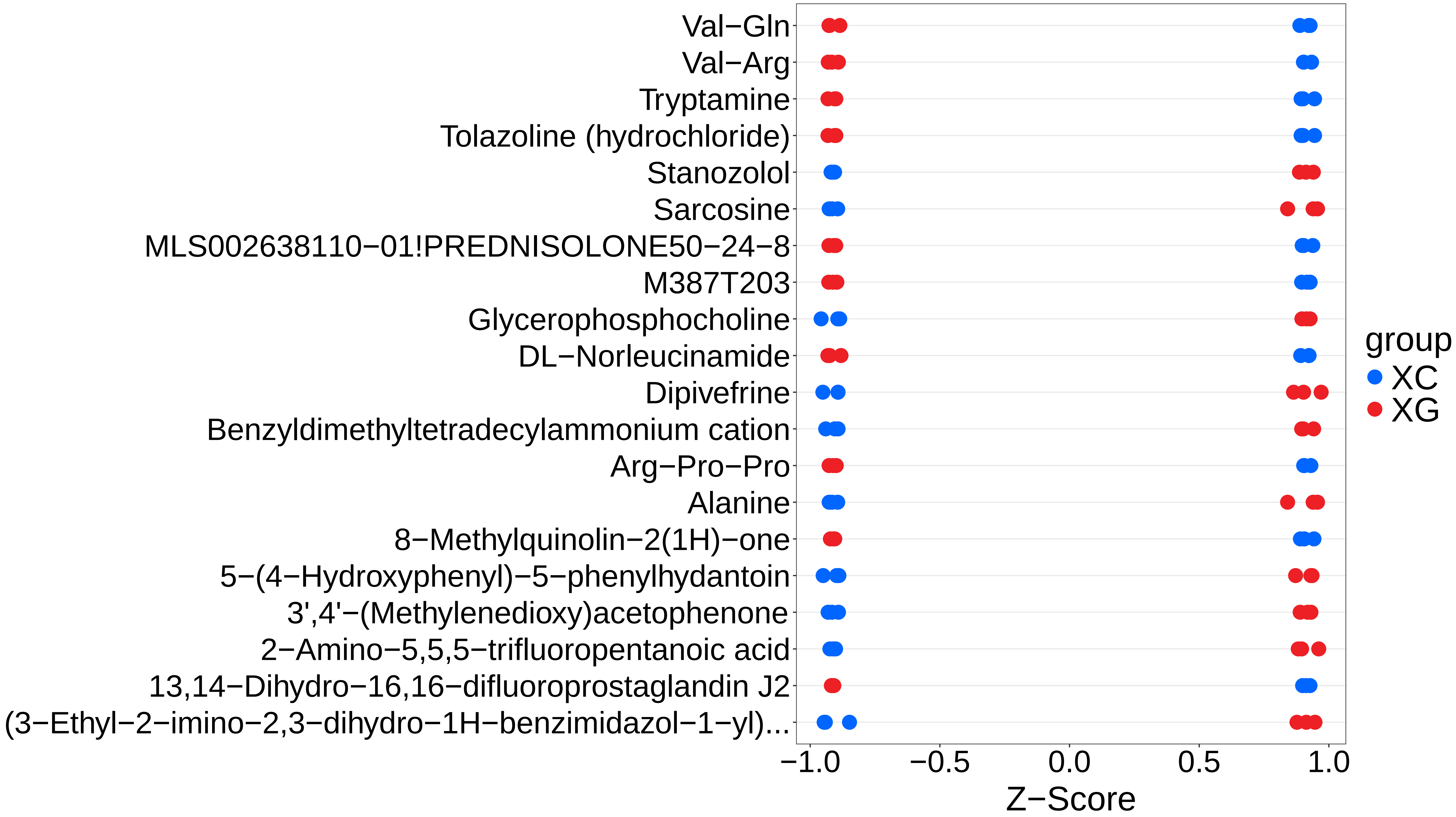
Val−Gln
Val−Arg
Tryptamine
Tolazoline (hydrochloride)
Stanozolol
Sarcosine
MLS002638110−01!PREDNISOLONE50−24−8
M387T203
Glycerophosphocholine
DL−Norleucinamide
Dipivefrine
Benzyldimethyltetradecylammonium cation
Arg−Pro−Pro
Alanine
8−Methylquinolin−2(1H)−one
5−(4−Hydroxyphenyl)−5−phenylhydantoin
3',4'−(Methylenedioxy)acetophenone
2−Amino−5,5,5−trifluoropentanoic acid
13,14−Dihydro−16,16−difluoroprostaglandin J2
(3−Ethyl−2−imino−2,3−dihydro−1H−benzimidazol−1−yl)...
−1.0
−0.5
0.0
0.5
1.0
Z−Score
group
XC
XG
