# Supplementary material for: Comparative Physiological and Metabolomic Responses of Procambarus clarkii and Macrobrachium nipponense to Combined Salinity–pH and NaHCO3 Alkalinity Stress
Source: Animals (Basel). 2026 Sep 3;16(17):2778. doi: 10.3390/ani16172778 (PMC13565080; doi:10.3390/ani16172778)
Supplement: Supplementary file 1 [file animals-16-02778-s001.zip › Statistical Analysis/XCvsXJ/OPLS-DA score plot.pdf]

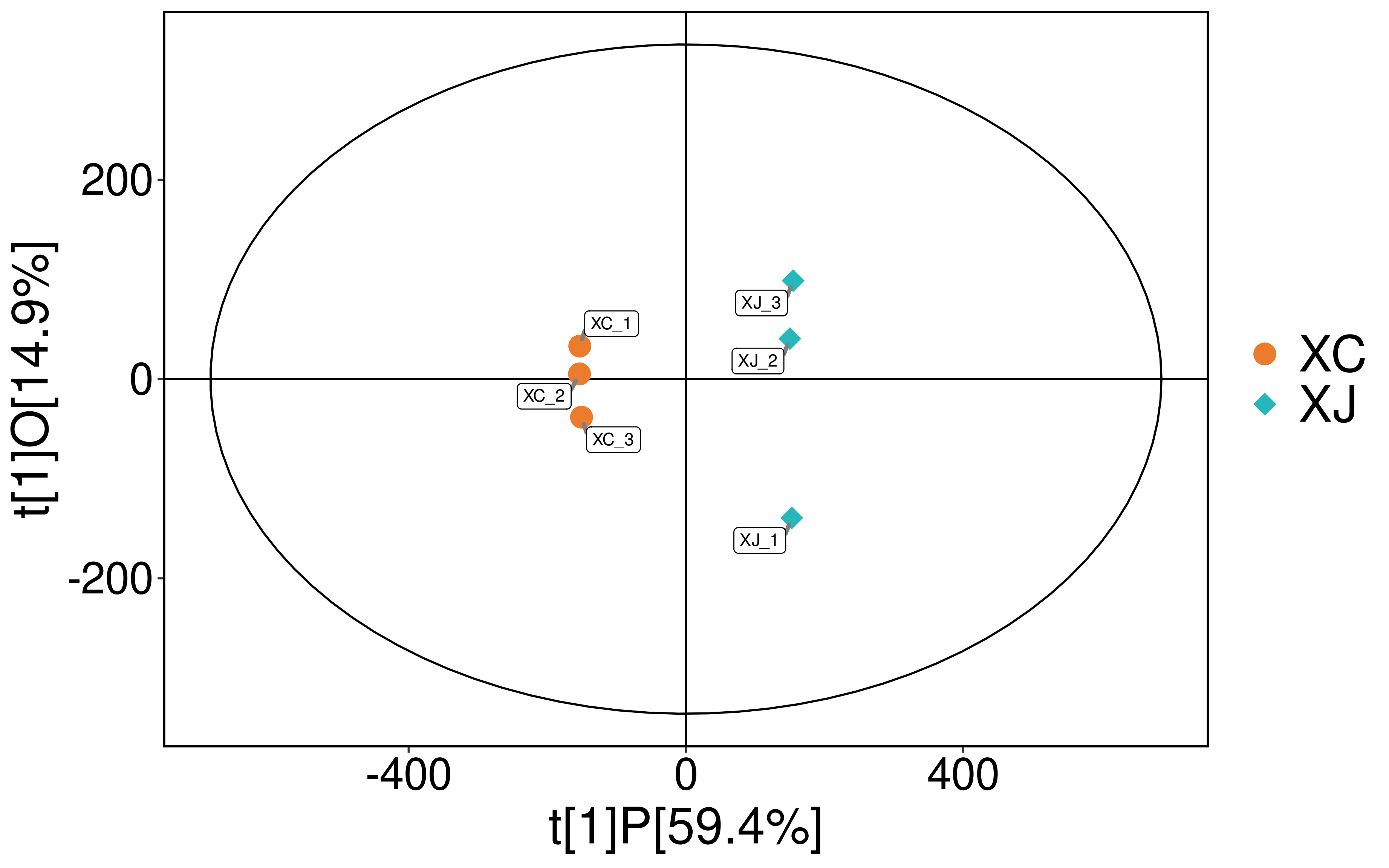

200
0
-200
-400
0
400
t[1]O[14.9%]
t[1]P[59.4%]
XC_1
XC_2
XC_3
XJ_1
XJ_2
XJ_3
XC
XJ
