# Supplementary material for: Comparative Physiological and Metabolomic Responses of Procambarus clarkii and Macrobrachium nipponense to Combined Salinity–pH and NaHCO3 Alkalinity Stress
Source: Animals (Basel). 2026 Sep 3;16(17):2778. doi: 10.3390/ani16172778 (PMC13565080; doi:10.3390/ani16172778)
Supplement: Supplementary file 1 [file animals-16-02778-s001.zip › Statistical Analysis/XCvsXJ/volcano plot with label.pdf]

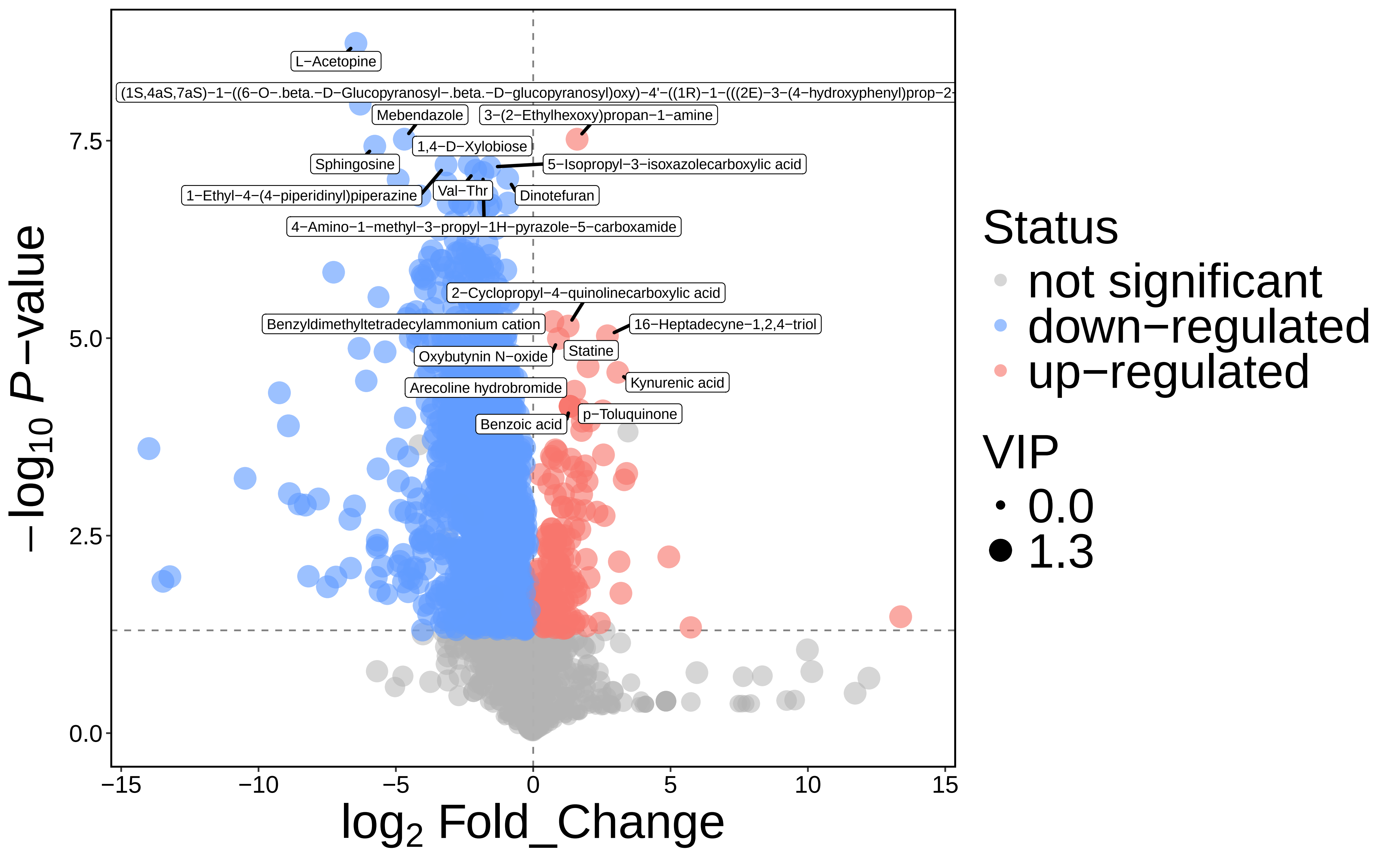
L−Acetopine
(1S,4aS,7aS)−1−((6−O−.beta.−D−Glucopyranosyl−.beta.−D−glucopyranosyl)oxy)−4'−((1R)−1−(((2E)−3−(4−hydroxyphenyl)prop−2−
Mebendazole
3−(2−Ethylhexoxy)propan−1−amine
1,4−D−Xylobiose
Sphingosine
5−Isopropyl−3−isoxazolecarboxylic acid
1−Ethyl−4−(4−piperidinyl)piperazine
Val−Thr
Dinotefuran
4−Amino−1−methyl−3−propyl−1H−pyrazole−5−carboxamide
2−Cyclopropyl−4−quinolinecarboxylic acid
Benzyldimethyltetradecylammonium cation
16−Heptadecyne−1,2,4−triol
Oxybutynin N−oxide
Statine
Kynurenic acid
Arecoline hydrobromide
p−Toluquinone
Benzoic acid
−log10 P−value
7.5
5.0
2.5
0.0
−15
−10
−5
0
5
10
15
log2 Fold_Change
Status
not significant
down−regulated
up−regulated
VIP
0.0
1.3
