# Supplementary material for: Comparative Physiological and Metabolomic Responses of Procambarus clarkii and Macrobrachium nipponense to Combined Salinity–pH and NaHCO3 Alkalinity Stress
Source: Animals (Basel). 2026 Sep 3;16(17):2778. doi: 10.3390/ani16172778 (PMC13565080; doi:10.3390/ani16172778)
Supplement: Supplementary file 1 [file animals-16-02778-s001.zip › Statistical Analysis/XCvsXJ/Z-Score Plot.pdf]

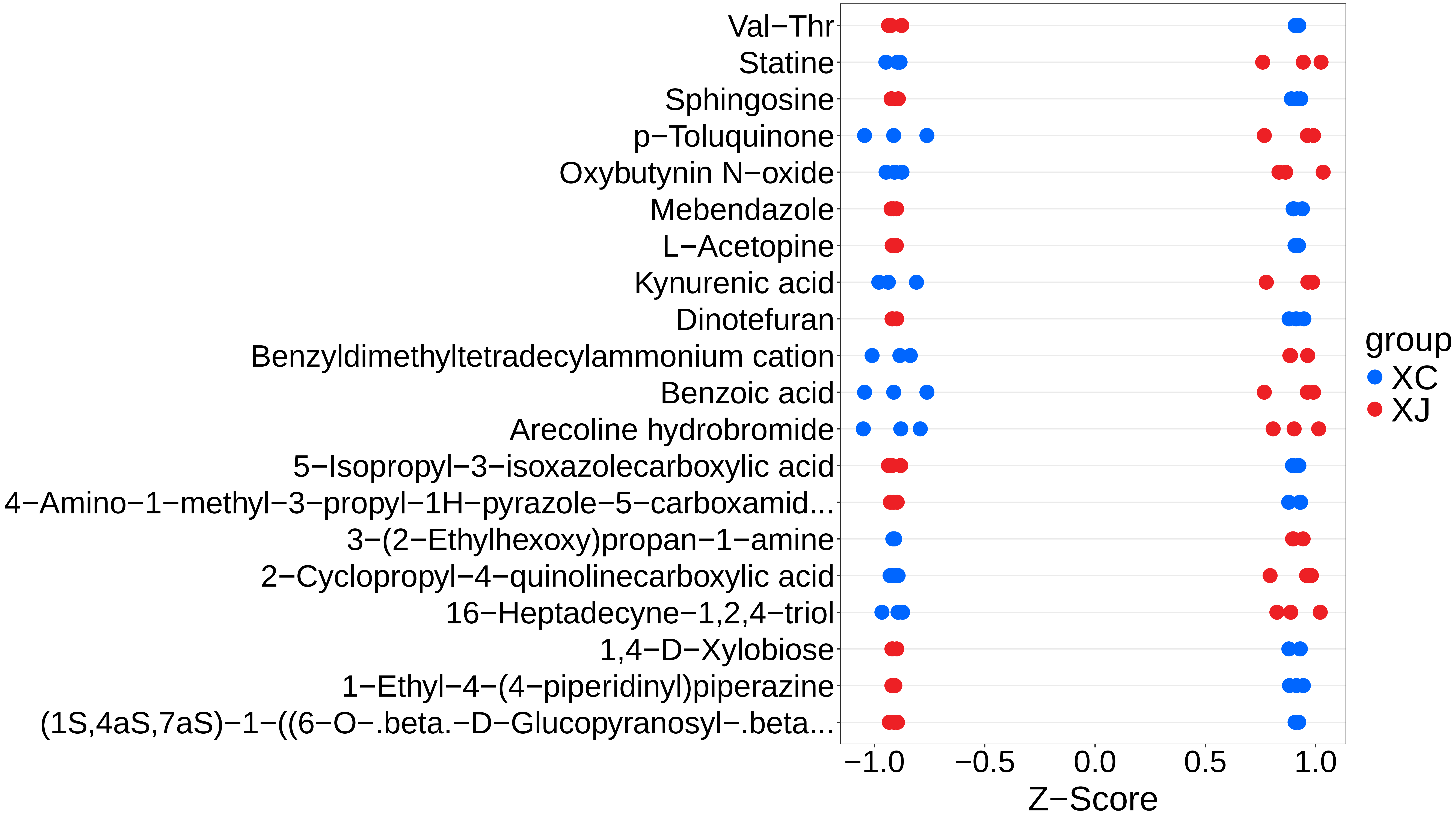

Val−Thr
Statine
Sphingosine
p−Toluquinone
Oxybutynin N−oxide
Mebendazole
L−Acetopine
Kynurenic acid
Dinotefuran
Benzyldimethyltetradecylammonium cation
Benzoic acid
Arecoline hydrobromide
5−Isopropyl−3−isoxazolecarboxylic acid
4−Amino−1−methyl−3−propyl−1H−pyrazole−5−carboxamid...
3−(2−Ethylhexoxy)propan−1−amine
2−Cyclopropyl−4−quinolinecarboxylic acid
16−Heptadecyne−1,2,4−triol
1,4−D−Xylobiose
1−Ethyl−4−(4−piperidinyl)piperazine
(1S,4aS,7aS)−1−((6−O−.beta.−D−Glucopyranosyl−.beta...
−1.0
−0.5
0.0
0.5
1.0
Z−Score
group
XC
XJ
