# Supplementary material for: Comparative Physiological and Metabolomic Responses of Procambarus clarkii and Macrobrachium nipponense to Combined Salinity–pH and NaHCO3 Alkalinity Stress
Source: Animals (Basel). 2026 Sep 3;16(17):2778. doi: 10.3390/ani16172778 (PMC13565080; doi:10.3390/ani16172778)
Supplement: Supplementary file 1 [file animals-16-02778-s001.zip › Enrichment Analysis/Q_CvsQG/KEGG Enrichment bubble.pdf]

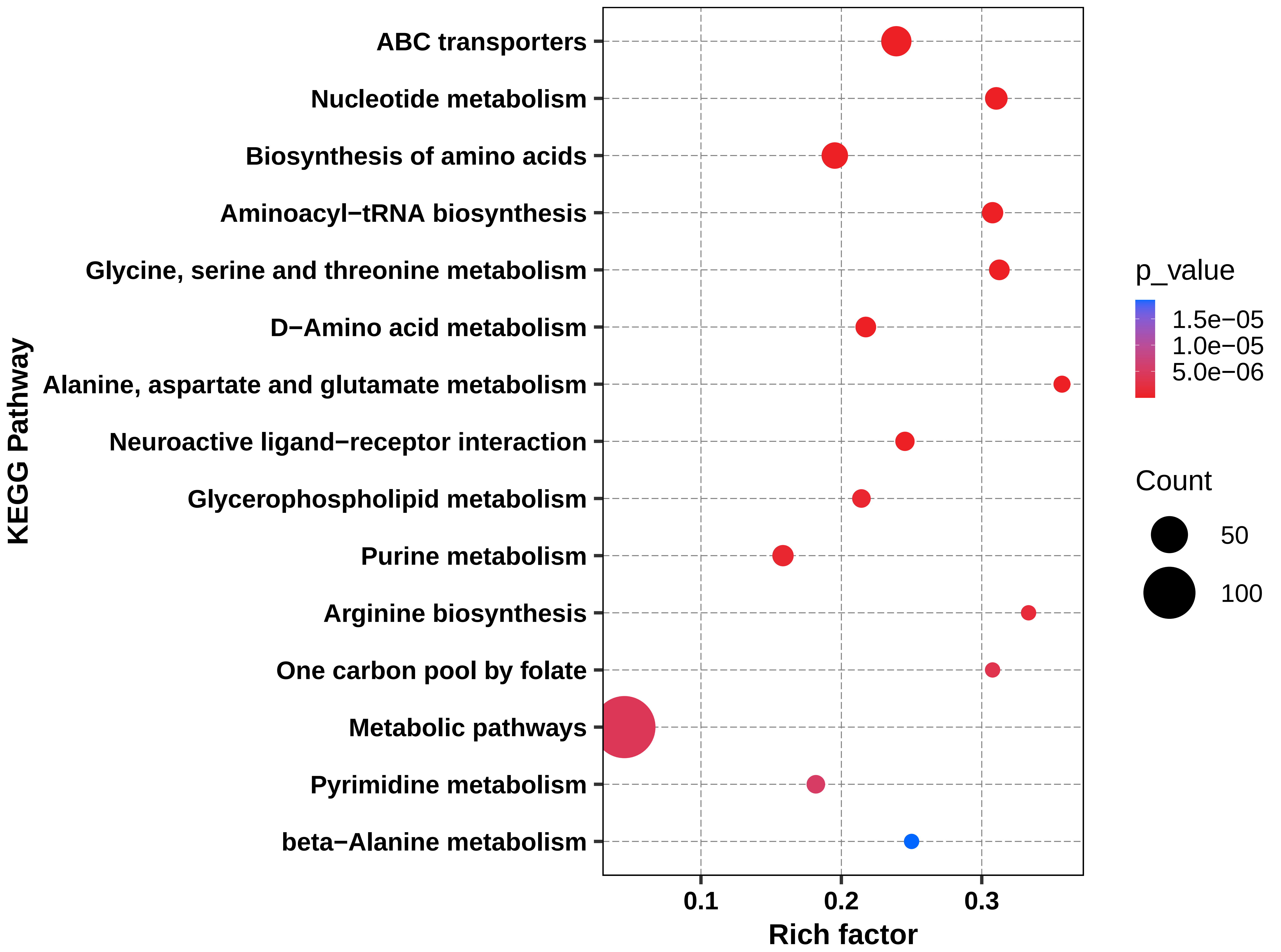
KEGG Pathway
ABC transporters
Nucleotide metabolism
Biosynthesis of amino acids
Aminoacyl−tRNA biosynthesis
Glycine, serine and threonine metabolism
D−Amino acid metabolism
Alanine, aspartate and glutamate metabolism
Neuroactive ligand−receptor interaction
Glycerophospholipid metabolism
Purine metabolism
Arginine biosynthesis
One carbon pool by folate
Metabolic pathways
Pyrimidine metabolism
beta−Alanine metabolism
0.1
0.2
0.3
Rich factor
p_value
1.5e−05
1.0e−05
5.0e−06
Count
50
100
