# Supplementary material for: Comparative Physiological and Metabolomic Responses of Procambarus clarkii and Macrobrachium nipponense to Combined Salinity–pH and NaHCO3 Alkalinity Stress
Source: Animals (Basel). 2026 Sep 3;16(17):2778. doi: 10.3390/ani16172778 (PMC13565080; doi:10.3390/ani16172778)
Supplement: Supplementary file 1 [file animals-16-02778-s001.zip › Enrichment Analysis/Q_CvsQG/KEGG heatmap.pdf]

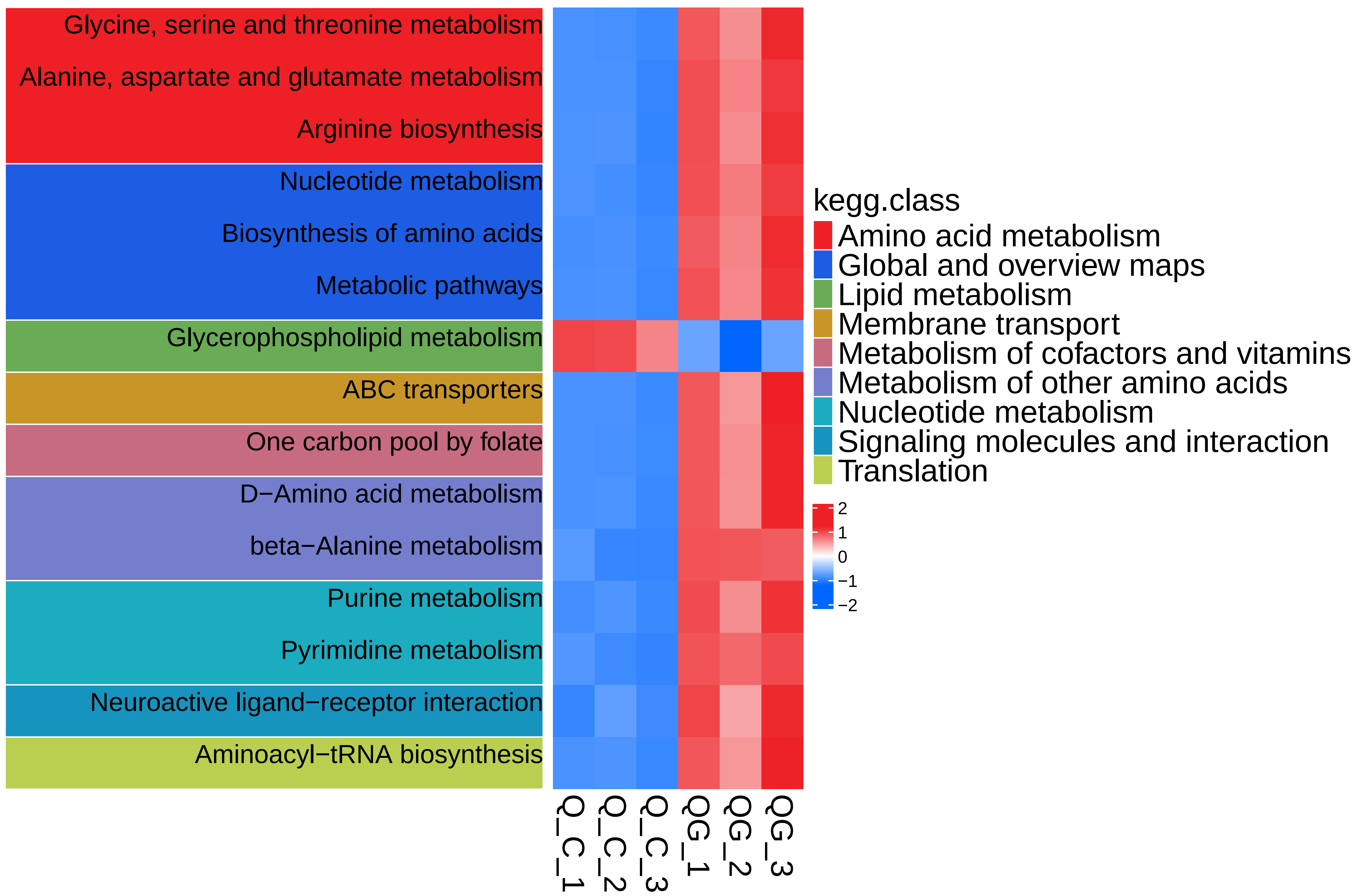

Glycine, serine and threonine metabolism
Alanine, aspartate and glutamate metabolism
Arginine biosynthesis
Nucleotide metabolism
Biosynthesis of amino acids
Metabolic pathways
Glycerophospholipid metabolism
ABC transporters
One carbon pool by folate
D−Amino acid metabolism
beta−Alanine metabolism
Purine metabolism
Pyrimidine metabolism
Neuroactive ligand−receptor interaction
Aminoacyl−tRNA biosynthesis
Q_C_1
Q_C_2
Q_C_3
QG_1
QG_2
QG_3
kegg.class
Amino acid metabolism
Global and overview maps
Lipid metabolism
Membrane transport
Metabolism of cofactors and vitamins
Metabolism of other amino acids
Nucleotide metabolism
Signaling molecules and interaction
Translation
2
1
0
−1
−2
