# Supplementary material for: Comparative Physiological and Metabolomic Responses of Procambarus clarkii and Macrobrachium nipponense to Combined Salinity–pH and NaHCO3 Alkalinity Stress
Source: Animals (Basel). 2026 Sep 3;16(17):2778. doi: 10.3390/ani16172778 (PMC13565080; doi:10.3390/ani16172778)
Supplement: Supplementary file 1 [file animals-16-02778-s001.zip › Enrichment Analysis/Q_CvsQJ/KEGG Classification.pdf]

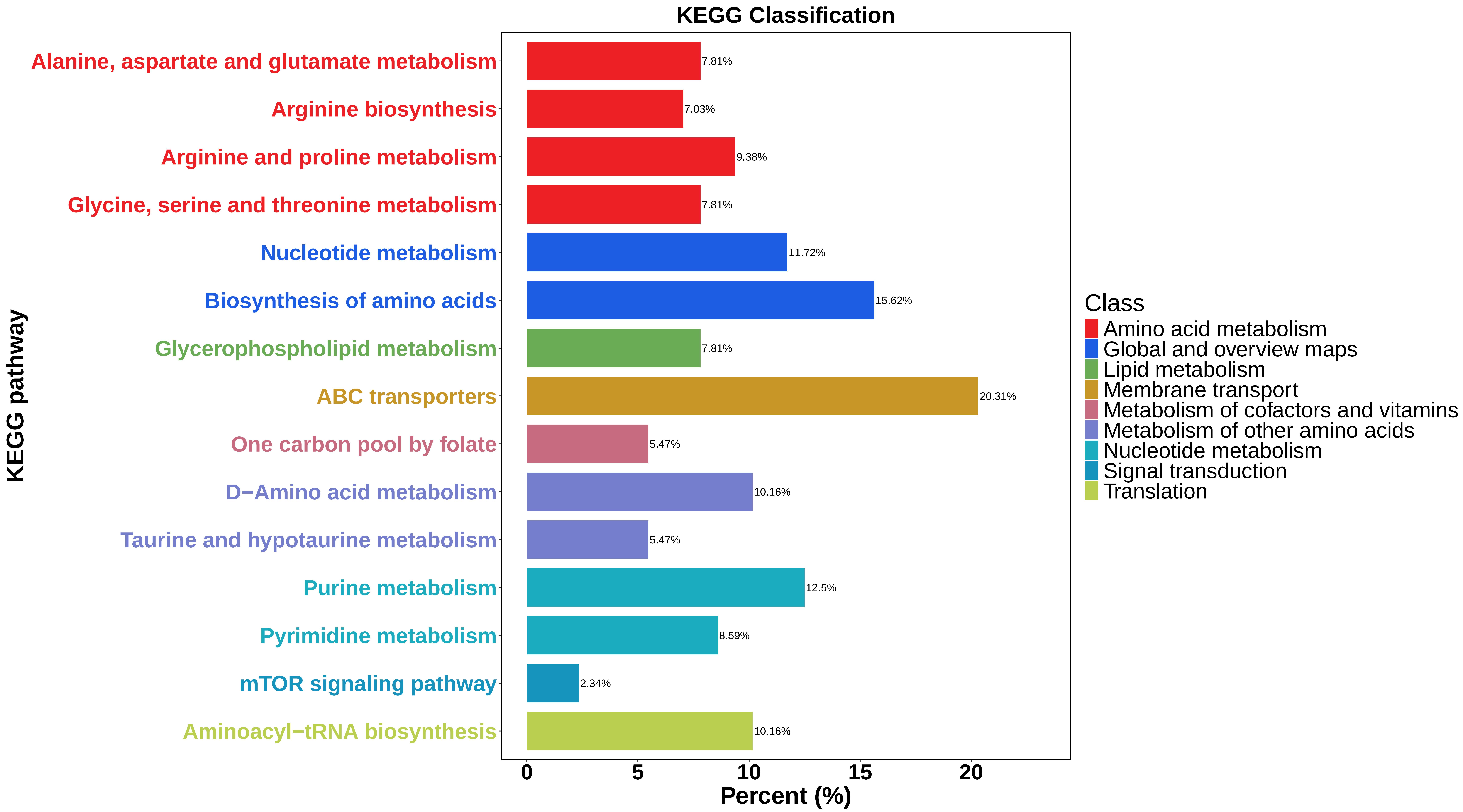
KEGG Classification
KEGG pathway
Alanine, aspartate and glutamate metabolism
Arginine biosynthesis
Arginine and proline metabolism
Glycine, serine and threonine metabolism
Nucleotide metabolism
Biosynthesis of amino acids
Glycerophospholipid metabolism
ABC transporters
One carbon pool by folate
D−Amino acid metabolism
Taurine and hypotaurine metabolism
Purine metabolism
Pyrimidine metabolism
mTOR signaling pathway
Aminoacyl−tRNA biosynthesis
7.81%
7.03%
9.38%
7.81%
11.72%
15.62%
7.81%
20.31%
5.47%
10.16%
5.47%
12.5%
8.59%
2.34%
10.16%
0
5
10
15
20
Percent (%)
Class
Amino acid metabolism
Global and overview maps
Lipid metabolism
Membrane transport
Metabolism of cofactors and vitamins
Metabolism of other amino acids
Nucleotide metabolism
Signal transduction
Translation
