# Supplementary material for: Comparative Physiological and Metabolomic Responses of Procambarus clarkii and Macrobrachium nipponense to Combined Salinity–pH and NaHCO3 Alkalinity Stress
Source: Animals (Basel). 2026 Sep 3;16(17):2778. doi: 10.3390/ani16172778 (PMC13565080; doi:10.3390/ani16172778)
Supplement: Supplementary file 1 [file animals-16-02778-s001.zip › Enrichment Analysis/Q_CvsQJ/KEGG Enrichment bubble.pdf]

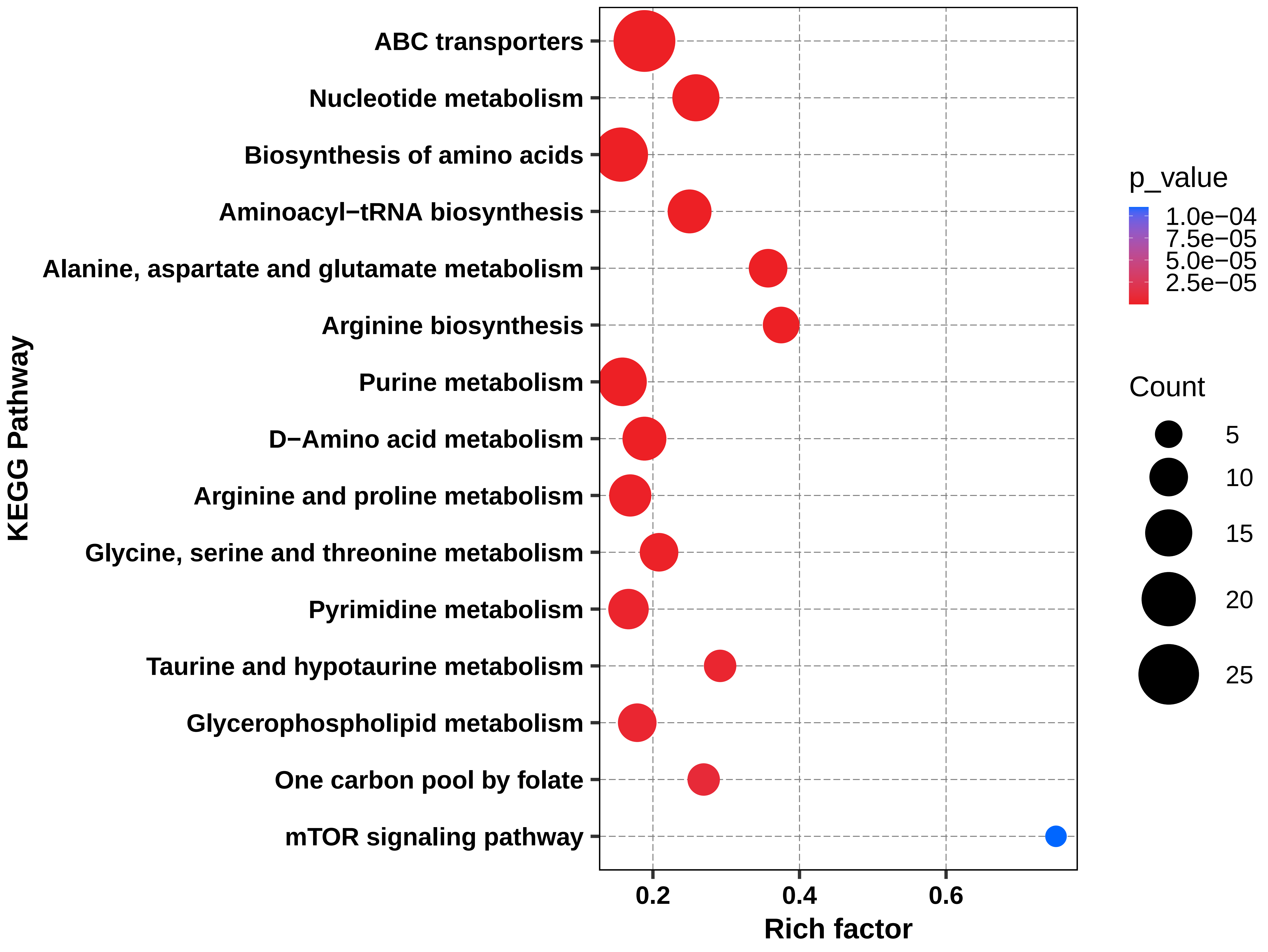
ABC transporters
Nucleotide metabolism
Biosynthesis of amino acids
Aminoacyl−tRNA biosynthesis
Alanine, aspartate and glutamate metabolism
Arginine biosynthesis
Purine metabolism
D−Amino acid metabolism
Arginine and proline metabolism
Glycine, serine and threonine metabolism
Pyrimidine metabolism
Taurine and hypotaurine metabolism
Glycerophospholipid metabolism
One carbon pool by folate
mTOR signaling pathway
KEGG Pathway
0.2
0.4
0.6
Rich factor
p_value
1.0e−04
7.5e−05
5.0e−05
2.5e−05
Count
5
10
15
20
25
