# Supplementary material for: Comparative Physiological and Metabolomic Responses of Procambarus clarkii and Macrobrachium nipponense to Combined Salinity–pH and NaHCO3 Alkalinity Stress
Source: Animals (Basel). 2026 Sep 3;16(17):2778. doi: 10.3390/ani16172778 (PMC13565080; doi:10.3390/ani16172778)
Supplement: Supplementary file 1 [file animals-16-02778-s001.zip › Enrichment Analysis/Q_CvsQJ/KEGG heatmap.pdf]

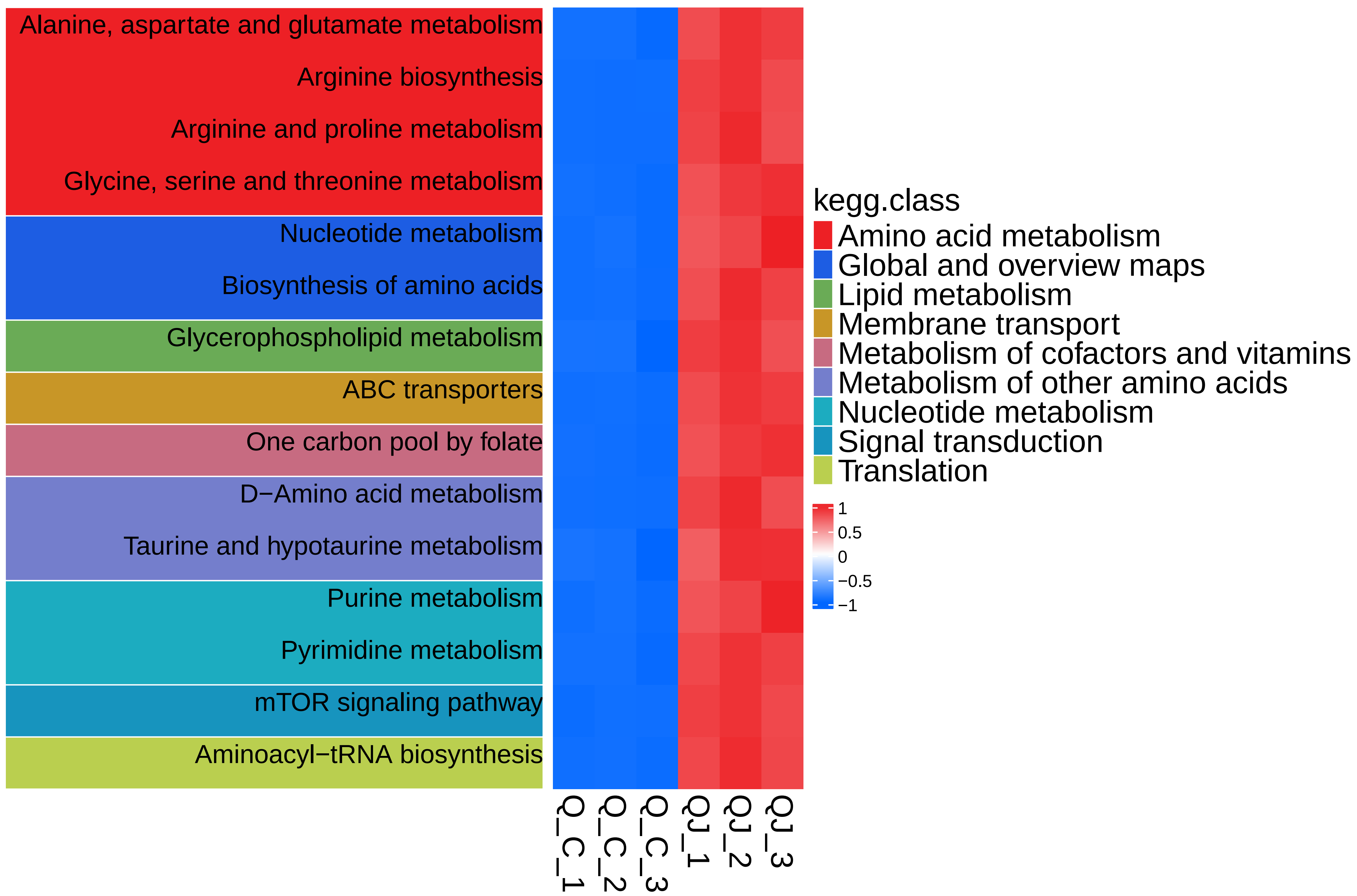

Alanine, aspartate and glutamate metabolism
Arginine biosynthesis
Arginine and proline metabolism
Glycine, serine and threonine metabolism
Nucleotide metabolism
Biosynthesis of amino acids
Glycerophospholipid metabolism
ABC transporters
One carbon pool by folate
D−Amino acid metabolism
Taurine and hypotaurine metabolism
Purine metabolism
Pyrimidine metabolism
mTOR signaling pathway
Aminoacyl−tRNA biosynthesis
Q_C_1
Q_C_2
Q_C_3
QJ_1
QJ_2
QJ_3
kegg.class
Amino acid metabolism
Global and overview maps
Lipid metabolism
Membrane transport
Metabolism of cofactors and vitamins
Metabolism of other amino acids
Nucleotide metabolism
Signal transduction
Translation
1
0.5
0
−0.5
−1
