# Supplementary material for: Comparative Physiological and Metabolomic Responses of Procambarus clarkii and Macrobrachium nipponense to Combined Salinity–pH and NaHCO3 Alkalinity Stress
Source: Animals (Basel). 2026 Sep 3;16(17):2778. doi: 10.3390/ani16172778 (PMC13565080; doi:10.3390/ani16172778)
Supplement: Supplementary file 1 [file animals-16-02778-s001.zip › Enrichment Analysis/XCvsXG/KEGG DA Score plot.pdf]

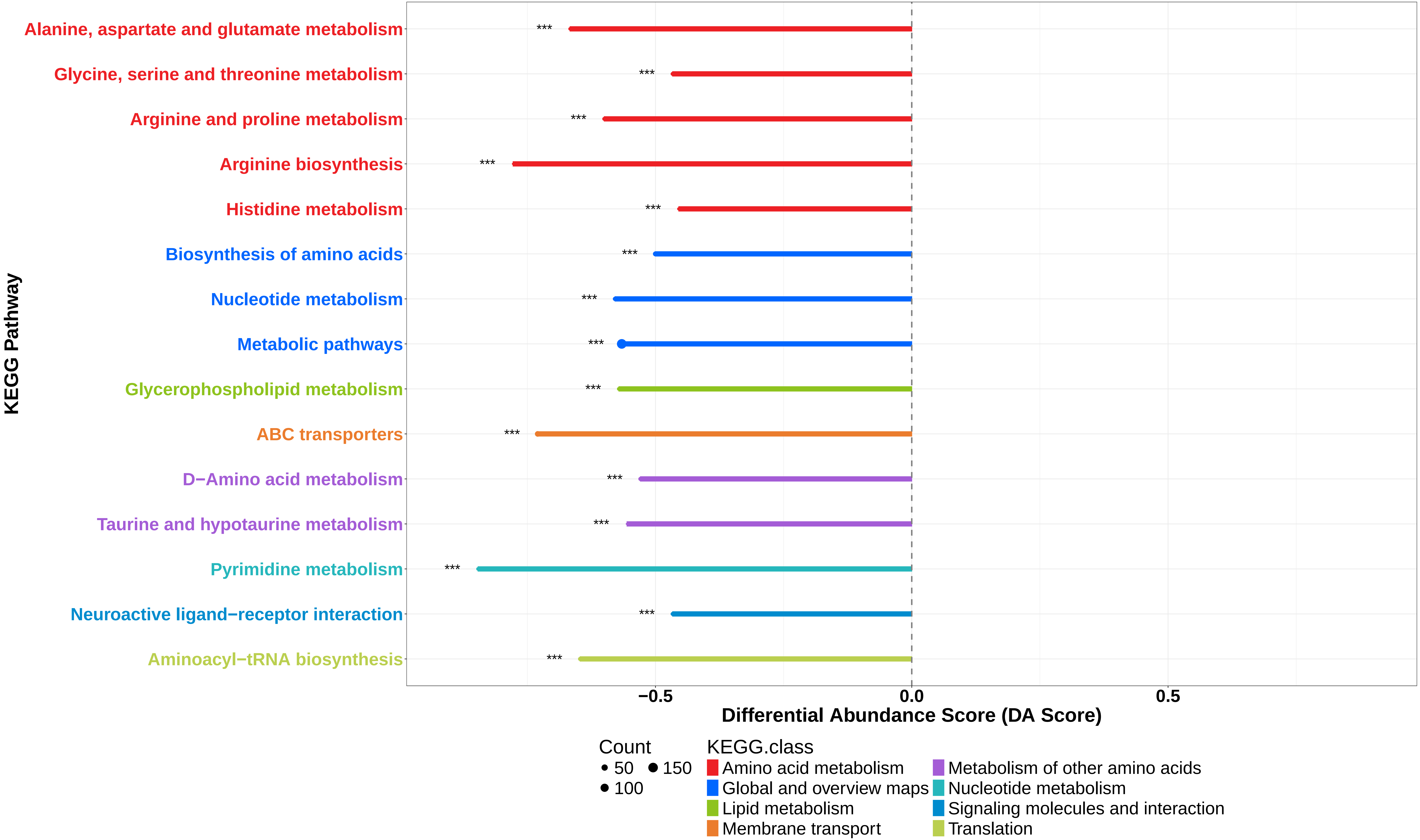
KEGG Pathway
Alanine, aspartate and glutamate metabolism
Glycine, serine and threonine metabolism
Arginine and proline metabolism
Arginine biosynthesis
Histidine metabolism
Biosynthesis of amino acids
Nucleotide metabolism
Metabolic pathways
Glycerophospholipid metabolism
ABC transporters
D−Amino acid metabolism
Taurine and hypotaurine metabolism
Pyrimidine metabolism
Neuroactive ligand−receptor interaction
Aminoacyl−tRNA biosynthesis
***
−0.5
0.0
0.5
Differential Abundance Score (DA Score)
Count
50
100
150
KEGG.class
Amino acid metabolism
Global and overview maps
Lipid metabolism
Membrane transport
Metabolism of other amino acids
Nucleotide metabolism
Signaling molecules and interaction
Translation
