# Supplementary material for: Comparative Physiological and Metabolomic Responses of Procambarus clarkii and Macrobrachium nipponense to Combined Salinity–pH and NaHCO3 Alkalinity Stress
Source: Animals (Basel). 2026 Sep 3;16(17):2778. doi: 10.3390/ani16172778 (PMC13565080; doi:10.3390/ani16172778)
Supplement: Supplementary file 1 [file animals-16-02778-s001.zip › Enrichment Analysis/XCvsXG/KEGG Enrichment bubble.pdf]

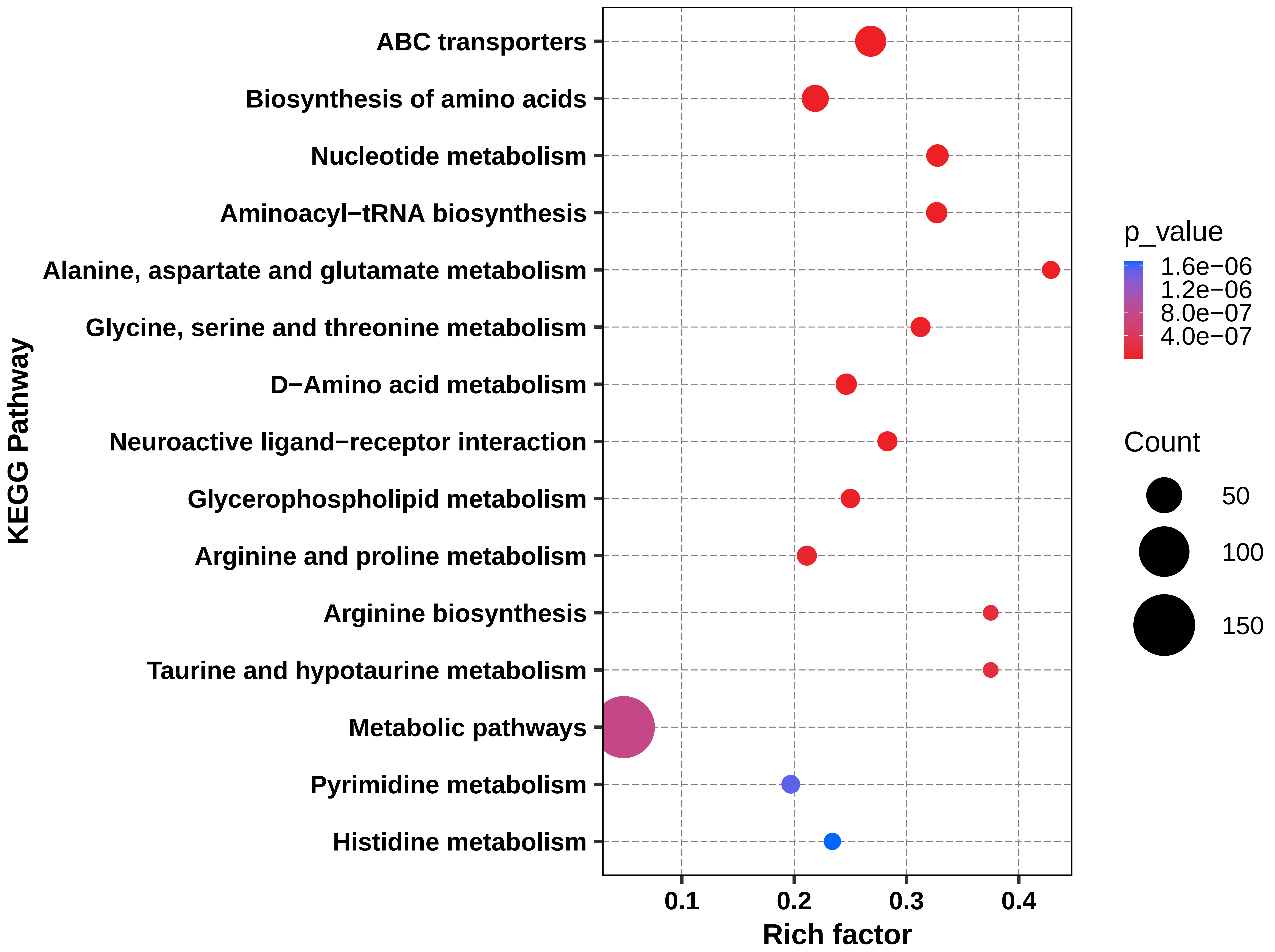

KEGG Pathway
ABC transporters
Biosynthesis of amino acids
Nucleotide metabolism
Aminoacyl−tRNA biosynthesis
Alanine, aspartate and glutamate metabolism
Glycine, serine and threonine metabolism
D−Amino acid metabolism
Neuroactive ligand−receptor interaction
Glycerophospholipid metabolism
Arginine and proline metabolism
Arginine biosynthesis
Taurine and hypotaurine metabolism
Metabolic pathways
Pyrimidine metabolism
Histidine metabolism
0.1
0.2
0.3
0.4
Rich factor
p_value
1.6e−06
1.2e−06
8.0e−07
4.0e−07
Count
50
100
150
