# Supplementary material for: Comparative Physiological and Metabolomic Responses of Procambarus clarkii and Macrobrachium nipponense to Combined Salinity–pH and NaHCO3 Alkalinity Stress
Source: Animals (Basel). 2026 Sep 3;16(17):2778. doi: 10.3390/ani16172778 (PMC13565080; doi:10.3390/ani16172778)
Supplement: Supplementary file 1 [file animals-16-02778-s001.zip › Enrichment Analysis/XCvsXJ/KEGG DA Score plot.pdf]

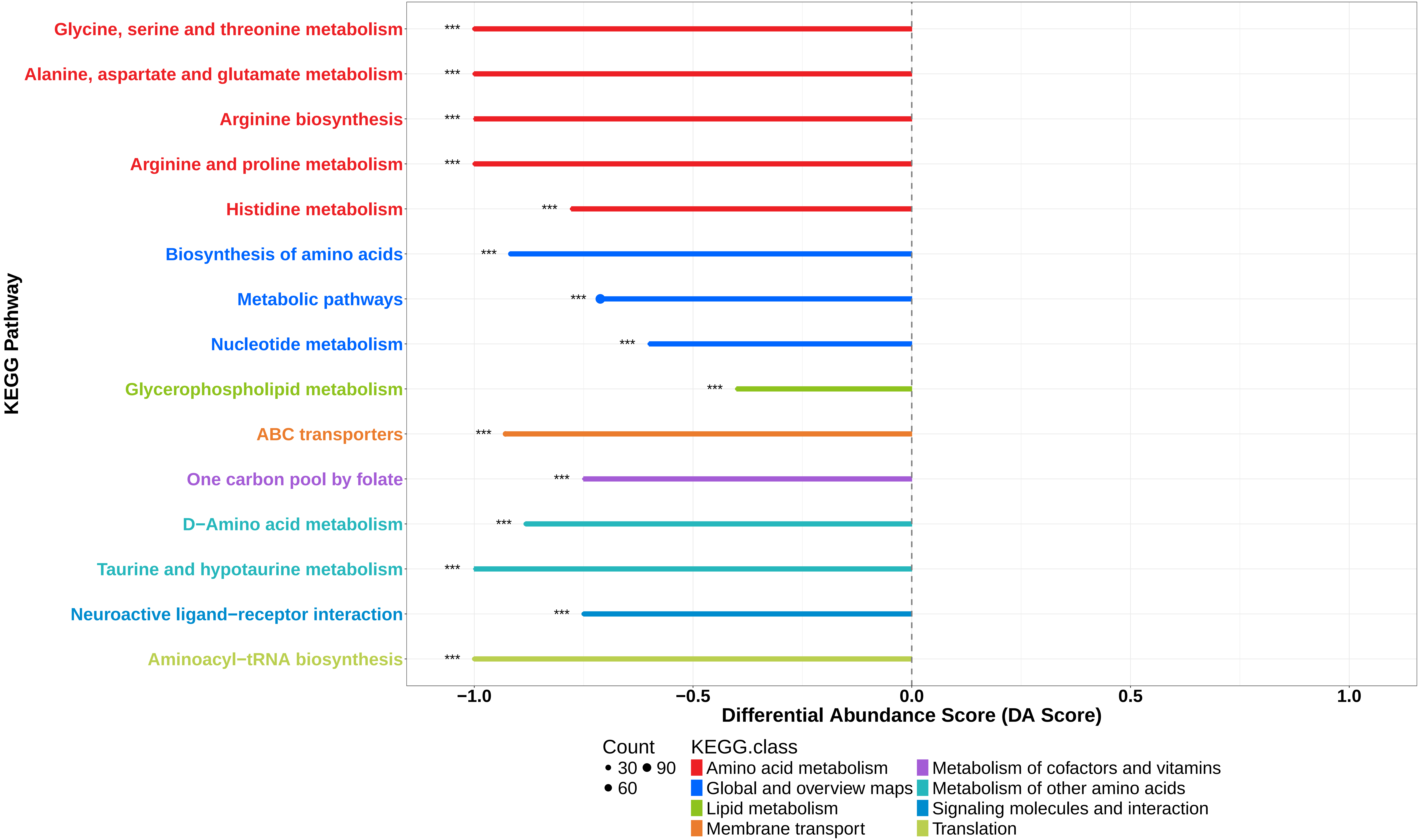
KEGG Pathway
Glycine, serine and threonine metabolism
Alanine, aspartate and glutamate metabolism
Arginine biosynthesis
Arginine and proline metabolism
Histidine metabolism
Biosynthesis of amino acids
Metabolic pathways
Nucleotide metabolism
Glycerophospholipid metabolism
ABC transporters
One carbon pool by folate
D−Amino acid metabolism
Taurine and hypotaurine metabolism
Neuroactive ligand−receptor interaction
Aminoacyl−tRNA biosynthesis
***
−1.0
−0.5
0.0
0.5
1.0
Differential Abundance Score (DA Score)
Count
30
60
90
KEGG.class
Amino acid metabolism
Global and overview maps
Lipid metabolism
Membrane transport
Metabolism of cofactors and vitamins
Metabolism of other amino acids
Signaling molecules and interaction
Translation
