# Supplementary material for: Comparative Physiological and Metabolomic Responses of Procambarus clarkii and Macrobrachium nipponense to Combined Salinity–pH and NaHCO3 Alkalinity Stress
Source: Animals (Basel). 2026 Sep 3;16(17):2778. doi: 10.3390/ani16172778 (PMC13565080; doi:10.3390/ani16172778)
Supplement: Supplementary file 1 [file animals-16-02778-s001.zip › Enrichment Analysis/XCvsXJ/KEGG Enrichment bubble.pdf]

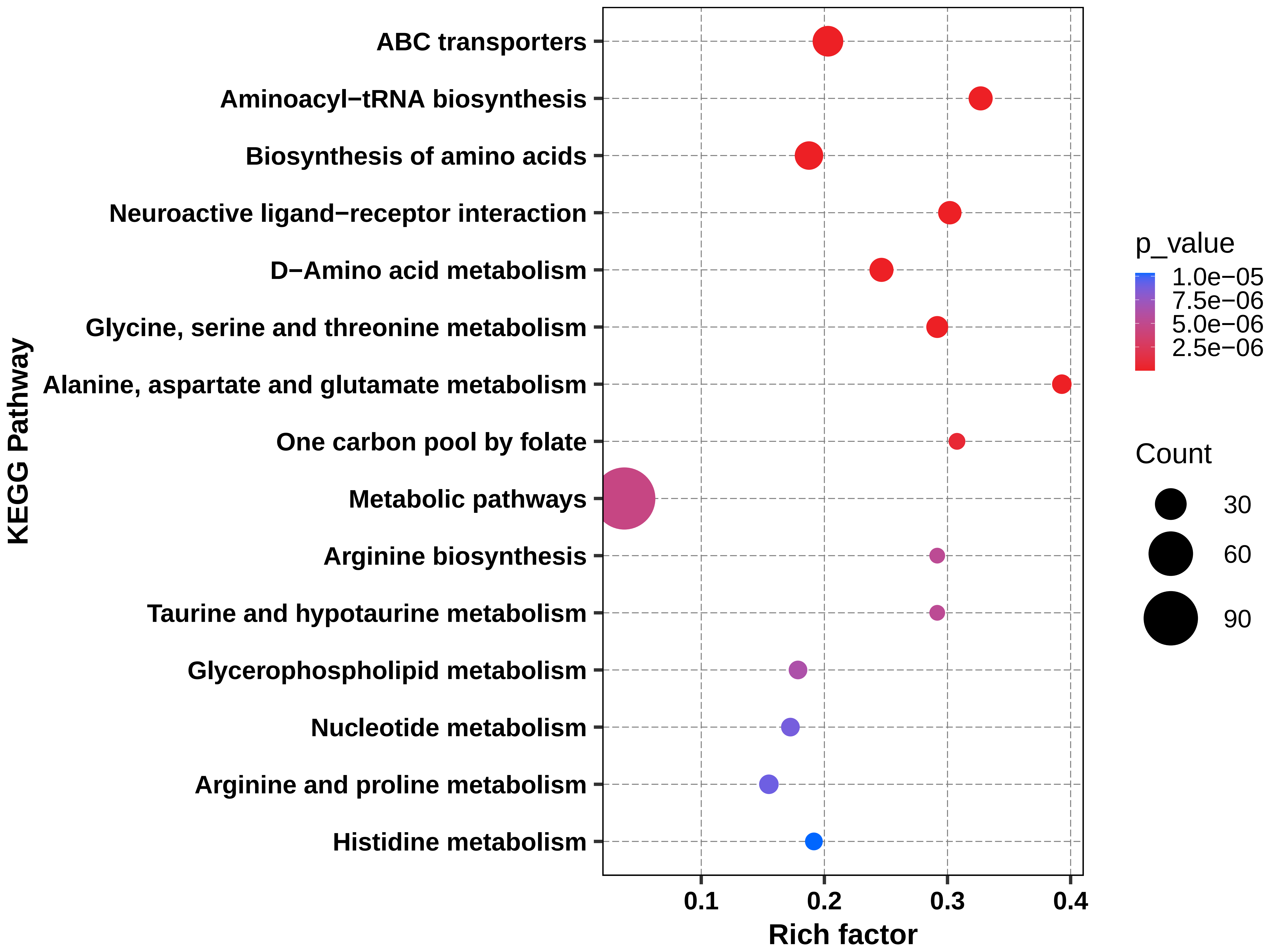

ABC transporters
Aminoacyl−tRNA biosynthesis
Biosynthesis of amino acids
Neuroactive ligand−receptor interaction
D−Amino acid metabolism
Glycine, serine and threonine metabolism
Alanine, aspartate and glutamate metabolism
One carbon pool by folate
Metabolic pathways
Arginine biosynthesis
Taurine and hypotaurine metabolism
Glycerophospholipid metabolism
Nucleotide metabolism
Arginine and proline metabolism
Histidine metabolism
KEGG Pathway
0.1
0.2
0.3
0.4
Rich factor
p_value
1.0e−05
7.5e−06
5.0e−06
2.5e−06
Count
30
60
90
