# Supplementary material for: Comparative Physiological and Metabolomic Responses of Procambarus clarkii and Macrobrachium nipponense to Combined Salinity–pH and NaHCO3 Alkalinity Stress
Source: Animals (Basel). 2026 Sep 3;16(17):2778. doi: 10.3390/ani16172778 (PMC13565080; doi:10.3390/ani16172778)
Supplement: Supplementary file 1 [file animals-16-02778-s001.zip › Experiment Analysis/PCA1 score plot.pdf]

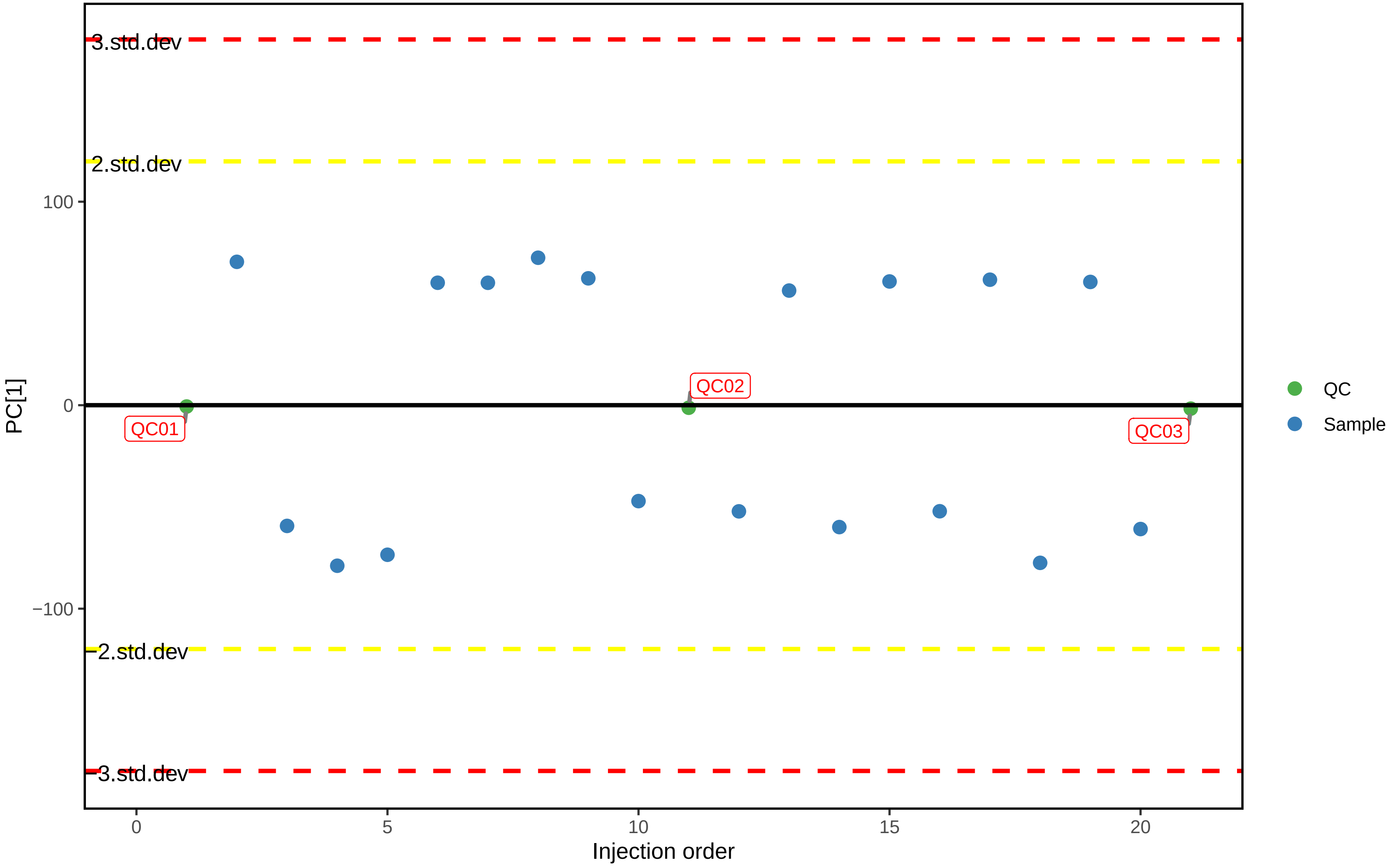

3.std.dev
2.std.dev
-2.std.dev
-3.std.dev
QC01
QC02
QC03
100
0
−100
PC[1]
0
5
10
15
20
Injection order
QC
Sample
