# Supplementary material for: Comparative Physiological and Metabolomic Responses of Procambarus clarkii and Macrobrachium nipponense to Combined Salinity–pH and NaHCO3 Alkalinity Stress
Source: Animals (Basel). 2026 Sep 3;16(17):2778. doi: 10.3390/ani16172778 (PMC13565080; doi:10.3390/ani16172778)
Supplement: Supplementary file 1 [file animals-16-02778-s001.zip › Experiment Analysis/PCA2 score plot.pdf]

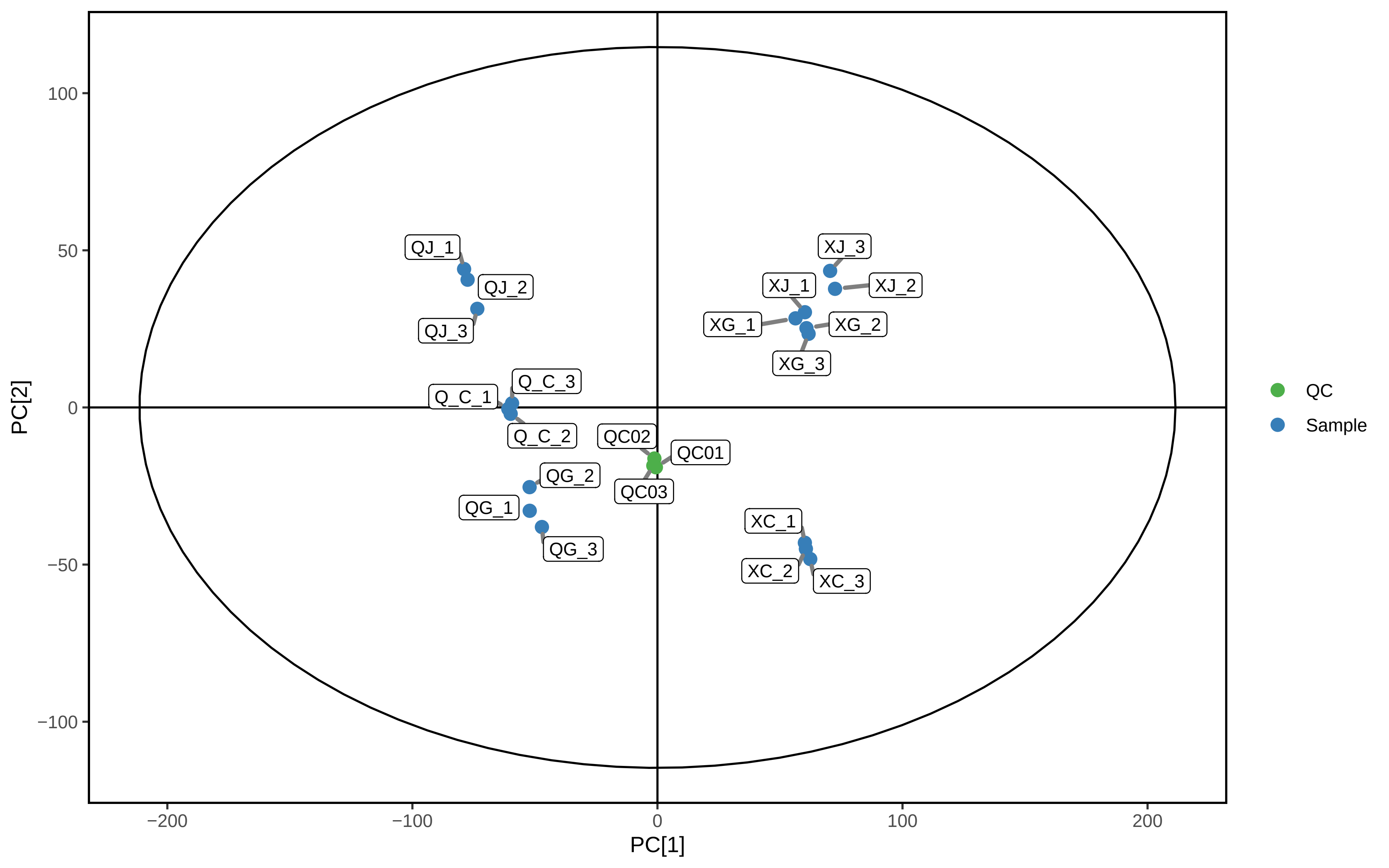
100
50
0
−50
−100
PC[2]
−200
−100
0
100
200
PC[1]
QJ_1
QJ_2
QJ_3
XJ_3
XJ_1
XJ_2
XG_1
XG_2
XG_3
Q_C_3
Q_C_1
Q_C_2
QC02
QC01
QC03
QG_2
QG_1
QG_3
XC_1
XC_2
XC_3
QC
Sample
